# A physiologically constrained calibration framework for cardiovascular models applied to a paediatric synthetic sepsis population

**DOI:** 10.1371/journal.pcbi.1014847

**Published:** 2026-10-07

**Authors:** Manuel T. Cabeleira, Samiran Ray, Nicholas C. Ovenden, Vanessa Díaz-Zuccarini

**Affiliations:** 1 Department of Mechanical Engineering, University College London, London, United Kingdom; 2 Hawkes Institute, University College London, London, United Kingdom; 3 Paediatric Intensive Care Unit, Great Ormond Street Hospital for Children NHS Foundation Trust, London, United Kingdom; 4 Department of Mathematics, University College London, London, United Kingdom; University of Notre Dame, UNITED STATES OF AMERICA

## Abstract

Calibration of mechanistic cardiovascular models is a central barrier to their use in population analysis and patient-specific simulation, particularly in settings where key physiological variables are unobservable and multiple parameter combinations can reproduce the same haemodynamic targets. In this work, we present Embedded Feedback Controller (EFC), a calibration framework for ODE-based lumped-parameter cardiovascular models in which selected physiological parameters are promoted to dynamic states and driven toward prescribed targets through embedded controller equations. By exploiting the qualitative structure of the governing equations, EFC enforces physiologically consistent parameter-variable relationships and converges to the single solution admitted by an over-determined set of targets, reproducibly and independently of the initial conditions, at a cost that scales efficiently with model complexity. The framework is demonstrated in silico, using a mechanistic cardiovascular model to generate virtual paediatric populations spanning normal physiology and two septic shock phenotypes (warm and cold shock) from literature-derived target ranges, achieving <1% residual error across pressures, flows, and compartmental volumes. The resulting parameter distributions are consistent, by construction with theoretical haemodynamic adaptations in paediatric sepsis, including alterations in vascular resistance, compliance, cardiac elastance, and effective blood volume. Importantly, persistent calibration residuals arise when target combinations are structurally incompatible with the model and the parameter carrying the residual is held at a bound, providing an explicit and interpretable diagnostic of feasibility limits. These results establish EFC as a general, scalable calibration strategy for mechanistic cardiovascular models and a practical foundation for virtual population generation and future patient-specific digital twin applications in critical care.

## Author summary

We present a new way to calibrate mechanistic cardiovascular models by updating selected model parameters during simulation while preserving physiological relationships. This makes calibration robust to initial conditions and supports scalable generation of mechanistically interpretable virtual populations. We demonstrate the method in a closed-loop paediatric cardiovascular model and reproduce normal circulation and warm and cold septic shock phenotypes with low residual error and plausible parameter shifts. Residual mismatches remain diagnostically meaningful, flagging infeasible target combinations.

## 1. Introduction

Lumped parameter models of the cardiovascular system (CVS) have been proven to be able to generate outputs that can mimic physiological behaviour with a high degree of precision [1,2]. In this context, the CVS is usually conceptualised as an interconnected network of compartments, representing different parts of the CVS, mathematically formulated as systems of ordinary differential equations (ODEs) that exploit the fluid-electrical analogy, where pressures and flows of blood vessels are represented by their electrical analogues [3]. This modelling approach has found many applications in medical research over decades, being used in combination with more complex blood vessel models [4] or in CircAdapt case where a complex heart model is informed by lumped parameter model of the CVS [5]. As stand-alone models, these can be used for the exploration of non-linear ventricular interactions [6,7], orthostatic stress [8,9] modelling or cardiopulmonary interactions [10–13], where the crosstalk between the respiratory system and the CVS is explored. The addition of autonomic control to these models allows the study of the response to hypercapnia [14–16], obstructive sleep apnoea [17], cardiogenic shock [18] and other types of shock [19] and the response to fluid resuscitation technique [20].

Despite the proven versatility of these models in the medical field, efficient and fast parametrisation is still a challenge, with the majority of the literature reusing parameters found in previous publications without providing much insight into the behaviour of the model under different parameter regimes or under different physiological states [18,21]. With the advent of digital patient twins and personalized medicine, the difficulty in parameterising these models is hindering their translation to clinical practice in benefit of black box approaches. The examples in the literature that do provide solutions to the parametrisation of ODE models work on models of reduced size, for example, eight parameters in a coupled cardio-pulmonary model [22] and nine in a single ventricle model of the systemic circulation [23]. Some approaches use MCMC [24] and neural ODEs [25] to calibrate a model for acute circulatory failure, gradient-based optimisation with a multiple-shooting formulation to estimate parameters of hybrid neural ODE models [26], or Levenberg-Marquardt multiple shooting to identify respiratory and perfusion parameters from capnography [27]. When the models are of standard complexity, the literature provides insights into the parameter space and the most influential parameters via sensitivity analysis [17]. While these methods can be effective in specific settings, recent methodological guidelines for mechanistic cardiovascular modelling emphasise that calibration strategies should preserve physiological structure, maintain parameter interpretability, and explicitly expose identifiability and feasibility limits rather than relying on black-box optimisation approaches [28].

The problem in hand here is two-fold. Firstly, the most problematic aspect is the number of hidden variables (variables that are not measured) present in the standard-sized models, leading to the emergence of many redundant solutions when traditional optimisation techniques are used. One possible approach here is to simplify the model and thus reduce the number of hidden variables or, if the number of observations is large enough, remove ambiguity completely. A good example is the work of Tannenbaum et al. [19] where the CVS was simplified into a 3 compartment model. This simplification is also tempting when traditional optimisation techniques are used since these usually do not scale well with the number of parameters due to increasing computational requirements and dimensionality of the problem. Simplification is, however, not always possible as sometimes some hidden variables are fundamental for the model to accurately reflect human physiology. A good example is the work of Albanese et al. [15] and Lu et al. [11] where capillary compartments are fundamental for modelling gas exchange between the lungs and the blood, despite capillary blood volume and pressure being impossible to measure.

Secondly, an aspect that is commonly overlooked in the literature is the effect total blood volume (TBV) has in the system, with most works focusing on the ‘traditional’ 75 Kg adult male, or eliminating the need of volume variables altogether by using ODE systems where pressure is the main primitive [19]. This is done because the TBV and its distribution across the system cannot be directly measured. The use of volume based ODE systems in scenarios where the TBV is not fixed generates another source of ambiguity to the optimisation process and will also increase the number of parameters being estimated.

Septic shock provides a clinically relevant setting in which haemodynamic instability emerges from interacting changes in vascular tone, cardiac function, and TBV [29]. It is defined as a dysregulated host response to infection leading to life-threatening organ dysfunction [30], with septic shock representing a high-mortality subset characterised by severe circulatory, cellular, and metabolic abnormalities [31]. In paediatrics, recent international consensus criteria operationalise sepsis as suspected infection with a Phoenix Sepsis Score ≥2 and septic shock as sepsis with cardiovascular dysfunction (Phoenix cardiovascular subscore ≥1) [29], an approach that has been shown to improve the identification and prognostic stratification of paediatric sepsis compared with earlier IPSCC definitions [32]. In paediatric populations, sepsis remains a major cause of morbidity and mortality, accounting for more than 8% of paediatric intensive care unit admissions [33] and contributing substantially to global childhood mortality [34].

Clinically, septic shock is commonly classified into warm shock (WS) and cold shock (CS), reflecting distinct haemodynamic phenotypes [35], although clinical classification based on bedside signs alone has limited accuracy in identifying underlying haemodynamic states [33]. In Fig 1, the physiological mechanisms that lead to the WS and CS phenotypes are described. Warm shock is characterised by reduced systemic vascular resistance and preserved or elevated cardiac output, whereas cold shock is associated with increased vascular resistance and reduced cardiac output. Historically, early fluid resuscitation has been a central component of paediatric sepsis management [36]. However, subsequent evidence has demonstrated potential harm from aggressive fluid bolus therapy in specific settings [37], leading contemporary guidelines to recommend more cautious, context-dependent fluid strategies with early consideration of vasoactive support when shock persists [33].

**Fig 1:**
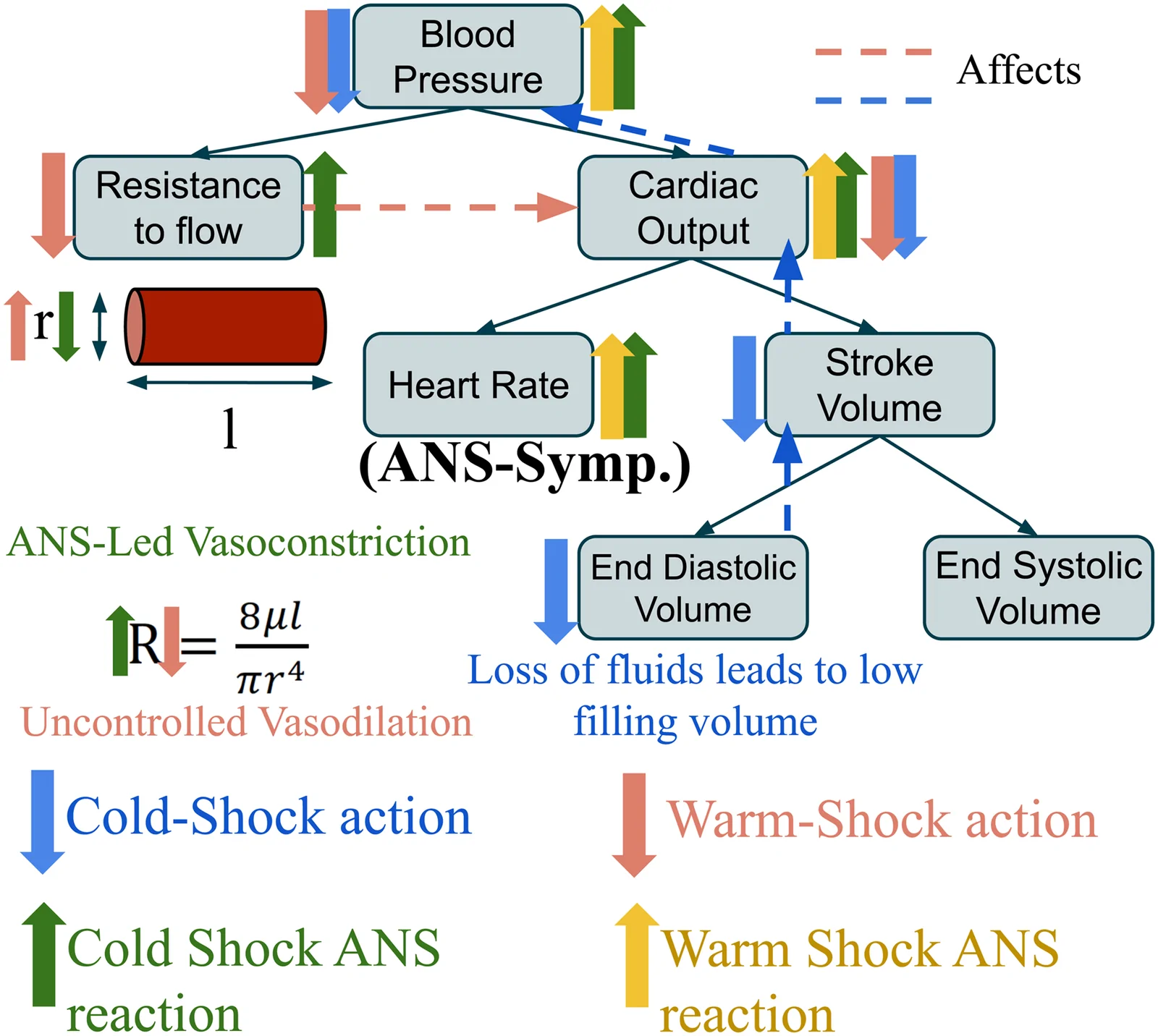
Schematic overview of the dominant physiological processes underlying warm and cold septic shock, used to explain the shock phenotypes. Septic shock phenotypes are characterised by inflammatory-driven vascular permeability and blood volume redistribution, leading to reduced cardiac filling and hypotension, with compensatory autonomic responses acting on heart rate, contractility, and vascular resistance. In warm shock, peripheral vasodilation limits effective vasoconstrictive compensation, whereas cold shock is associated with elevated systemic vascular resistance. The ANS reaction arrows explain how the observed heart rate, pressures and flows arise. No baroreflex loop is implemented in the model (See model schematic in Fig 2.).

Across both phenotypes, inflammatory-mediated increases in vascular permeability promote intravascular volume loss and redistribution into the interstitial space, reducing cardiac filling and arterial pressure. Compensatory autonomic responses attempt to restore perfusion through tachycardia, increased myocardial contractility, and vasoconstriction. In warm shock, however, these compensatory mechanisms may be ineffective due to uncontrolled peripheral vasodilation. Distinct underlying mechanisms like volume redistribution, vascular tone dysregulation, or impaired myocardial contractility can give rise to overlapping haemodynamic signatures. Because TBV and its distribution cannot be measured directly, these similarities complicate diagnosis and treatment selection. This ambiguity motivates the use of mechanistic modelling approaches capable of explicitly representing blood volume, cardiac function and vascular resistance within a unified physiological framework.

The marked heterogeneity of sepsis, the coexistence of distinct haemodynamic shock phenotypes, and the need to account for variations in total blood volume make paediatric sepsis a particularly compelling setting for the development and testing of calibration techniques, as disease manifestations are more dynamic, age-dependent, and less well characterised than in adult populations, with current diagnostic and modelling approaches remaining limited [38].

In this work, we present a detailed explanation of our calibration approach entitled ‘Embedded Feedback Controller’ EFC, which was initially introduced in our previous publication [10]. We then use this approach to generate a set of initial conditions that would be representative of a synthetic virtual population spanning the haemodynamic scenarios reported for paediatric sepsis, with admissible target ranges taken from the clinical literature. Specifically, we explain how first principles can be harnessed to identify the key model parameters and reveal their relationships with the observed variables and how to extend the number of observable variables for the optimisation problem. We will also demonstrate its independence of initial conditions and ability to generate unambiguous model solutions.

## 2. Materials and methods

Here, we first describe the clinical data routinely available in a paediatric intensive care setting and identify the subset of observables relevant to the proposed model. We then introduce the cardiovascular model formulation and its governing equations. Finally, we present the Embedded Feedback Controller calibration strategy and describe how physiological constraints are leveraged to generate patient-specific targets and virtual populations. All symbols, auxiliary operators, and time variables used in the paper are summarised in the nomenclature in Appendix A.1, Table 7.

### 2.1. Data available

A paediatric patient in a standard PICU is usually monitored by a combination of medical devices, depending on the condition and its severity. While continuous waveform recordings are technically feasible and available in some centres, their routine clinical use is limited by data volume, storage, and downstream usability constraints. As a result, many clinical data integration systems prioritise the recording of lower-frequency, trend-level physiological data, with sampling intervals on the order of seconds. The variables routinely recorded from these patients that are relevant for our model are:

- Systemic Arterial Blood Pressure (ABP) - 3 available pressures: systolic, diastolic and mean systemic blood pressures.
- Heart Rate (HR)
- Central Venous Pressure (CVP) - value of the mean blood pressure in the systemic venous system.
- Cardiac Output (CO) - A measure of the total amount of blood that flows through the heart over a minute. Commonly measured indirectly, including via echocardiography or less commonly, via tracer dilutional techniques based on the Fick principle.
- Pulmonary arterial Pressure (PAP) - usually extrapolated via echo-cardiography or directly measured using a Swan-Ganz catheter, although the use of this is very rare. In addition, the pulmonary capillary wedge pressure (PCWP), can also be obtained during balloon occlusion.

Patient demographics, including; age, height, weight, sex, are also collected and used to contextualise the values of the other variables collected or to estimate other parameters like TBV.

#### 2.1.1. Physiological priors derived from clinical practice.

Clinical monitoring in the intensive care setting provides access to only a limited subset of cardiovascular variables, whereas many physiologically relevant quantities, including blood volume distribution, regional pressure levels, and microcirculatory pressures, are not directly observable in routine practice. To address this gap, we introduce a set of physiological priors derived from established clinical knowledge and population-level studies. These priors represent plausible reference values and relationships that constrain otherwise unobserved quantities. Their role is to provide physiologically consistent bounds and relationships that will later be used to contextualise the available data and to support the construction of individualized cardiovascular representations.

**Capillary Pressures** While capillary pressure is not directly measurable in routine clinical settings, physiologically plausible target values can be inferred by considering the expected pressure gradients along the vascular tree as blood flows from the arterial to the venous circulation. This allows a pressure drop between the arterial and capillary beds to be prescribed, from which a corresponding range of capillary pressures can be derived.

**Total Blood Volume (TBV)** The cardiovascular system is commonly treated as conserving TBV over short time scales under stable conditions. However, the absolute blood volume of an individual is not directly measurable, nor is its distribution across the circulation. In clinical practice, the partial solution found for this is the estimation of TBV involving the use of different formulae for different patient populations using the patient’s sex, height and weight. For example using the Nadler’s formulae where height, weight are used to estimate TBV. Other methods use body weight alone as in Edelbi et al. [39] or in Raes et al. [40] where a known amount of albumin marked with iodine is injected and allowed to homogenise with the blood, the TBV is then estimated by calculating the diffused albumin concentration.

To estimate a possible value for the average blood volume within different regions of the circulation, the values of Table 1 were used, together with the estimation of the TBV. These assumptions provided a plausible value for every volume variable in the equation system, with the downside of considering every patient to be ‘normal’ in terms of the distribution of volume.

**Table 1:** Average blood volumes on different parts of the circulatory system, assuming a TBV of 700mL. Extracted from [41].

Cardiovascular	Volumes	
**Region**	mL	%
Left Heart	24.5	3.5
Right Heart	24.5	3.5
Systemic arteries (Aorta)	91	13
Systemic arterioles & capillaries	49	7
Systemic Veins	448	64
Pulmonary Arteries	26.6	3.8
Pulmonary Capillaries	9.8	1.4
Pulmonary Veins	26.6	3.8

**Flow Constraint** Over sufficiently long time scales, the average blood flow through the cardiovascular system is governed by the CO, which represents the volume of blood pumped by the heart per unit time. As a result, the mean flow supplying and draining the major regions of the circulation can be assumed to be equal to CO.

#### 2.1.2. Physiological target ranges for paediatric sepsis.

To characterise physiologically plausible ranges for the sepsis scenarios considered in this study, the clinical literature was surveyed and the resulting target values are summarised in Table 2.

**Table 2:** Target ranges used for calibration under warm shock, cold shock, and normal physiology.

Variable	Warm	Cold	Normal
TBV [41] (mL)	400–800	400–800	700–800
CO [42] (L/min)	2.75–3.5	1–2	1.75–3
HR ¶ [42] (bpm)	160–200	160–200	120–140
$SysP_{As}$ (mmHg)	60–80 [43]	60–80 [43]	75–90 [42]
$PP_{As}$†(mmHg)	$\frac{SysP_{As}}{2}$- $\frac{SysP_{As}}{1.75}$	$\frac{SysP_{As}}{4}$- $\frac{SysP_{As}}{3}$	$\frac{SysP_{As}}{3}$- $\frac{SysP_{As}}{2}$
CVP ($P_{Vs}$) [44] (mmHg)	4–10	4–10	8–12
$P_{Cs}^{*}$(mmHg)	0.3–0.7	0.3–0.7	0.3–0.7
$SysP_{Ap}$[45](mmHg)	20–25	20–25	20–25
$DiaP_{Ap}$(mmHg)	12–15	12–15	12–15
$P_{Cp}$[45](mmHg)	8–14	8–14	8–14

*As the capillary pressure of the systemic system is impossible to measure, we have imposed a % drop in pressure from the diastolic value of the Systemic arterial pressure and the value of CVP. ^†^ The targets for the Pulse Pressure of the systemic arterial blood pressure is calculated according to the equation extracted from Ranjit et al. [43] where these ratios were found to be descriptive of sepsis cases. ^¶^ HR is used as an input to the model.

**TBV** The reference subject was a 12 month old child with a body surface area of 0.5 *m*^2^ and therefore would have, in a healthy scenario 700–800 mL of TBV. As shock can happen in normovolemia and hypovolemia, the TBV of the shock cases was set between 400–800 *mL*.

**Cardiac output & HR** The cardiac index that is considered normal by Brierley et al. [46] is between 3.5 and 6.0, thus if WS is characterised by increased CO, it would have CI between 5.5 and 7 and CS having low CO would have a CI of 2–4. These limits were chosen so that there would be some overlap with the ‘normal’ class whilst allowing for the exploration of more extreme cases as well. According to sepsis guidelines, a heart rate above 160 is considered clinically concerning, while values exceeding 190 are regarded as an indicator of sepsis (in the context of suspected or proven infection) [42].

**Systemic blood pressures** The systolic blood pressure $SysP_{As}$ is considered clinically concerning when below 70 [42] to 75 mmHg [47] and the normal values were found to be between 75–90 mmHg [42]. As both shocks can have normal to low systolic blood pressure [43], the limits for these were set to 60–80 mmHg. Systemic pulse pressure ($PP_{As}$), defined as the difference between systolic and diastolic pressures in the systemic arteries, is considered clinically concerning when $PP_{As}>SysP_{As}/2$. In this context, warm shock is typically associated with normal to elevated $PP_{As}$, whereas cold shock is characterised by a narrowed $PP_{As}$[43]. For this we set the limits for PP as fractions of $SysP_{As}$ with the WS always being $>SysP_{As}/2$ and the CS $<SysP_{As}/3$. These pulse-pressure targets are inherently heuristic and may break down at elevated heart rates, where pressure-based indices no longer reliably reflect cardiac performance, as discussed by Tibby et al. [48].

After fluid resuscitation therapy, the values for CVP that lead to the best outcomes are observed to be between 8–12mmHg [44] and thus these were the limits set for ‘normal’. The shock cases having normal to low CVP, were set to 4–10 mmHg. As the pressure at the systemic capillaries is difficult to measure, the targets for these were set as a % pressure drop between the diastolic pressure and the CVP. The range for this % pressure drop was set to 0.3 to 0.7 in all cases to simulate both high and low capillary pressures.

**Pulmonary pressures** On the pulmonary system, sepsis specific studies where the pulmonary parameters are mentioned were not found, with the literature focusing more on pulmonary hypertension, with the guidelines stating that systolic Pulmonary Arterial Blood Pressure sPABP > 35mmHg [49,50] and mean pulmonary pressure > 20 mmHg [45] and Pulmonary Capillary Wedge Pressure PCWP target between 12–14 mmHg [45]. With this information we set the sPABP to be ‘normal’ in all subjects (20–25 mmHg) and the diastolic pressure to be 12–15 mmHg to allow some pressure drop before the capillaries. To these we set a target capillary pressure to be 1–4 mmHg lower than the diastolic pressure, generating capillary pressures with a range between 8–14 mmHg.

### 2.2. Cardiovascular system model

The physiological target ranges and population constraints defined above specify the haemodynamic conditions that the model must reproduce. We now introduce the cardiovascular system model and its governing equations, which provide the mechanistic basis for subsequent calibration.

The cardiovascular system model used in this work is illustrated in Fig 2. It represents the heart and circulation as a lumped-parameter network composed of interconnected compartments corresponding to the major functional regions of the cardiovascular system. Each compartment represents a spatially aggregated vascular or cardiac region and is characterised by a pressure, a volume, and associated inflow and outflow rates. The compartments are connected through resistive elements that govern blood transport between regions, while capacitive elements account for vascular and cardiac compliance.

**Fig 2:**
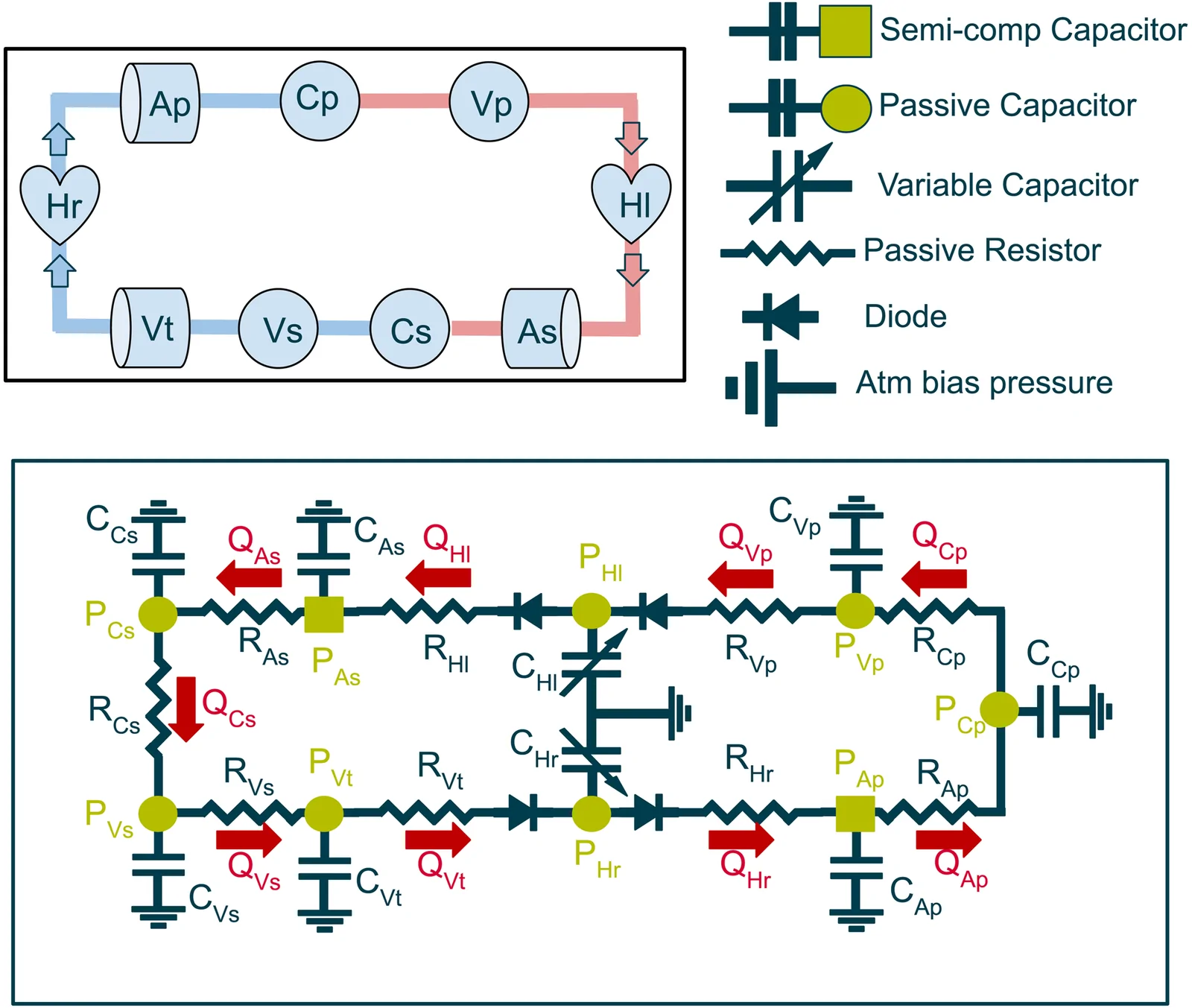
Schematic representation of the cardiovascular model. **Top:** High-level block diagram showing the major compartments of the systemic and pulmonary circulations, including the left and right heart chambers (Hl, Hr), arteries (As, Ap), capillaries (Cs, Cp), veins (Vs, Vp), and thoracic veins (Vt). **Bottom:** Full circuit representation of the model, where each compartment is represented by a compliance element and connected to neighbouring compartments through resistances that govern inflow and outflow.

This formulation results in a closed-loop circulation comprising nine compartments, and therefore nine state pressures and volumes, together with the flows that couple them. The model structure follows the same modelling strategy presented in [10], where the approach is described in greater detail. In the present work only the heart and circulation are represented, as these are the primary focus of the analysis.

The governing equations that define the pressure, volume, and flow dynamics of each compartment are introduced in Section 2.2.1. The heart chambers require additional treatment to capture their cyclic contraction and relaxation, which is addressed through time-varying elastance models and a dedicated heart-rate formulation described in Section 2.2.2. Finally, a set of auxiliary equations is introduced to compute cycle-based quantities such as extrema, averages, and integrals of physiological variables, which are required for analysis and calibration and are described in Section 2.2.3.

#### 2.2.1. Model equations.

The model shown in Fig 2 consists of nine compartments, each of which is described using the same generic compartment formulation illustrated in Fig 3.

**Fig 3:**
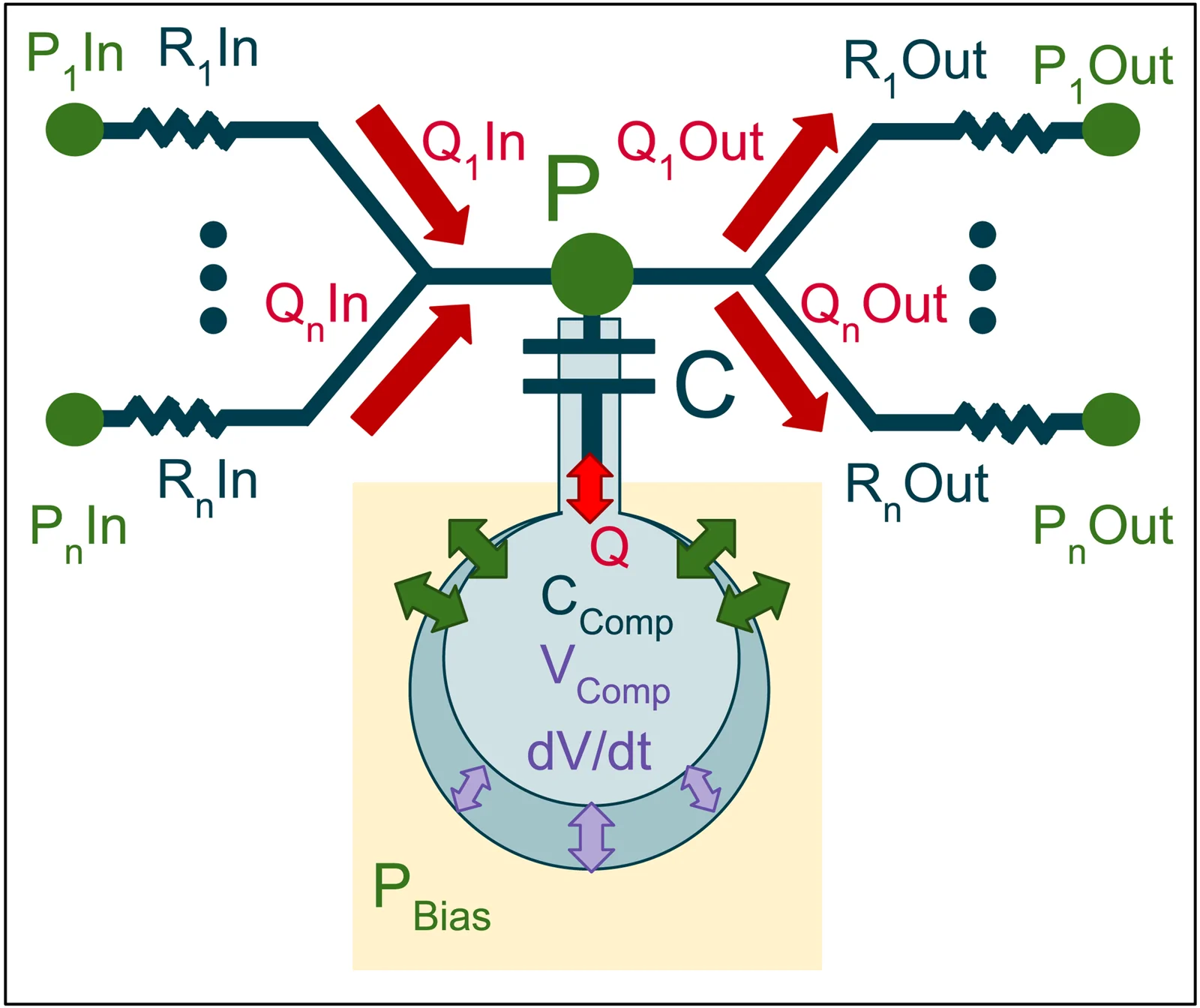
Generic representation of a compartment in the cardiovascular model. Each compartment is connected to its neighbours through a set of resistors, characterised by inlet and outlet resistances $R_{in}$ and $R_{out}$, which govern the inflow $Q_{in}$ and outflow $Q_{out}$ according to the pressure differences with neighbouring compartments ($P_{in}$, $P_{out}$). The central capacitor represents the compartment’s compliance *C*, which stores a volume of fluid and determines the local pressure ***P*.** Together, these elements define the fundamental pressure/volume/flow relationships used throughout the full model.

The equation system that describes the generic compartment is:


$$\begin{array}{c} P=\frac{\left(V-V_{0}\right)}{C}+P_{Bias}, \end{array}$$ (1)

$$\begin{array}{c} Q_{i}^{\text{in }}=\frac{P_{i}^{\text{in }}-P}{R_{i}^{\text{in }}},\;Q_{i}^{\text{out }}=\frac{P-P_{i}^{\text{out }}}{R_{i}^{\text{out }}},\;\text{for }i=1,2...n, \end{array}$$ (2)

$$\begin{array}{c} \frac{dV}{dt}=\sum_{i=1}^{n}Q_{i}^{\text{in }}-\sum_{i=1}^{n}Q_{i}^{\text{out }} \end{array}$$ (3)

In Equation 1, *P* denotes the pressure within the compartment, *V* the compartmental blood volume, *V*_0_ the unstressed volume, *C* the compliance, and $P_{Bias}$an additive pressure offset accounting for external reference pressures. Equation 2 defines the inflow and outflow rates $Q_{i}^{\text{in}}$ and $Q_{i}^{\text{out}}$ between the compartment and its *n* neighbouring compartments, driven by the pressure differences $\left(P_{i}^{\text{in}}-P\right)$ and $\left(P-P_{i}^{\text{out}}\right)$across the corresponding inlet and outlet resistances $R_{i}^{\text{in}}$ and $R_{i}^{\text{out}}$. The temporal evolution of the compartmental volume is then given by Equation 3, which enforces mass conservation by equating the rate of change of volume to the difference between total inflow and total outflow.

The resistors at the inlet and outlet of the heart compartments are modelled as diodes to prevent backflow of blood, ensuring unidirectional flow during the cardiac cycle.


$$\begin{array}{c} Q=\left\{ \begin{array}{cc} \frac{P_{1}-P}{R} & P_{1}-P>0,\\ 0 & P_{1}-P\leq 0, \end{array}\right. \end{array}$$ (4)

#### 2.2.2. Cycles: Heart rate modelling.

The heart functions as a muscle that operates in a periodic cycle of contraction (systole) and relaxation (diastole). Consequently, the compliances representing the heart chambers also have to be specifically tailored to account for the cyclic nature of cardiac function. In literature, the most common approach is to modulate the value of compliance using elastance models (ℰ=1/C). We use the variable-elastance model adapted from Heldt et al. [8] written as:


$$\begin{array}{cc} E_{rise}(\tau ) & =\frac{E-e}{2}\cdot \left[1-\cos(\frac{\pi \cdot \tau }{T_{rise}})\right]; \end{array}$$ (5)

$$\begin{array}{cc} E_{fall}(\tau ) & =\frac{E-e}{2}\cdot \left[1+\cos(\frac{\pi \cdot (\tau -T_{rise})}{T_{fall}})\right]; \end{array}$$ (6)

$$\begin{array}{cc} ℰ(\tau ) & =\left\{ \begin{array}{cc} e+E_{rise}(\tau ) & 0<\tau <T_{rise},\\ e+E_{fall}(\tau ) & T_{rise}\leq \tau \leq T_{rise}+T_{fall},\\ e & T_{rise}+T_{fall}\leq \tau . \end{array}\right. \end{array}$$ (7)

where *E* and *e* represent the maximum (systolic) and minimum (diastolic) elastances of the chamber, respectively, $T_{rise}$ and $T_{fall}$ define the durations of the systolic contraction and relaxation phases, ℰ(τ) represents the resulting time-varying elastance over the cardiac cycle. *HR* is the heart rate, *HC* the duration of a single heart cycle.

The heart elastance equations (7) require a time variable τ that represents the elapsed time within the current cardiac cycle rather than absolute simulation time. The duration of each cycle is determined from the heart rate according to


$$\begin{array}{c} HC=\frac{60}{HR}, \end{array}$$ (8)

where *HR* denotes the heart rate in beats per minute and *HC* the resulting duration of a single cardiac cycle.

To ensure that changes in heart rate affect only subsequent cardiac cycles and do not perturb the ongoing one, the model employs an event-triggered cycle mechanism. As illustrated in Fig 4, the trigger equation (9) schedules the onset time of the next cardiac cycle, stored in the variable $T_{trig}$, based on the current cycle duration. When the trigger condition is met, the timer equation (10) resets the intra-cycle time τ to zero, after which it advances linearly until the next trigger event. Reset operations are implemented as fast relaxations over an infinitesimal reset time ε, taken in the limit $\varepsilon \to 0^{+}$ and realised in the implementation as an exact reset applied at the cardiac boundary; ε is a property of the model and not the time step of the solver. This choice avoids explicit hybrid events while ensuring that cycle resets occur instantaneously at cardiac boundaries, without tying the model equations to the discretisation. Throughout, *t* denotes absolute simulation time, while τ represents the elapsed time within the current cardiac cycle and provides the phase variable used in the elastance model.


$$\begin{array}{cc} \frac{d\;T_{trig}}{dt} & =\left\{ \begin{array}{cc} \frac{HC}{\varepsilon } & t-T_{trig}(t)\geq 0\\ 0 & t-T_{trig}(t)<0 \end{array}\right. \end{array}$$ (9)

$$\begin{array}{cc} \frac{d\;\tau }{dt} & =\left\{ \begin{array}{cc} -\frac{\tau }{\varepsilon } & t-T_{trig}(t)\geq 0\\ 1 & t-T_{trig}(t)<0 \end{array}\right. \end{array}$$ (10)

**Fig 4:**
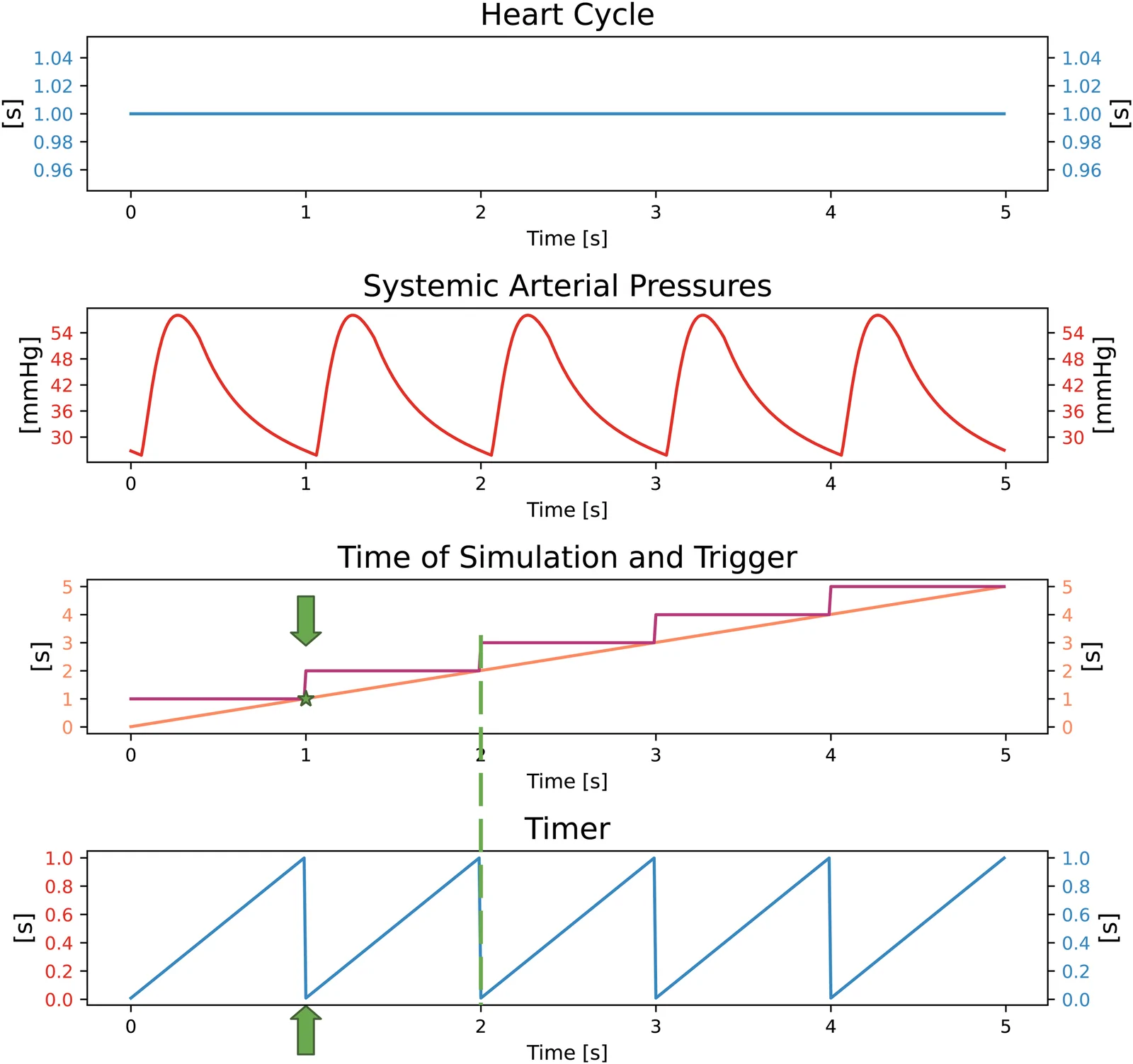
Schematic representation of the mechanism used to enable variable heart rates within the simulation framework. Top panel: prescribed heart-cycle duration. Second panel: resulting systemic arterial pressure waveform over consecutive cardiac cycles. Third panel: absolute simulation time (orange) and discrete trigger time (purple); when the trigger value coincides with the absolute time, it is advanced to the next scheduled trigger instant. Bottom panel: cycle timer, which resets to zero upon each trigger update and evolves linearly until the next cardiac event.

#### 2.2.3. Calculation of max, min, average and integration over cycle.

The trigger mechanism described earlier is also used to compute cycle-based physiological quantities required during simulation and calibration (Section 2.3). The trigger resets the evaluation window for maxima, minima, integrals, and other beat-to-beat metrics. The maximum and minimum of a variable *Y* are obtained using Eqs. 11, 12, which reset their values at each trigger and track the extremes until the next cycle.


$$\begin{array}{c} \frac{d\;minY}{dt}=\left\{ \begin{array}{cc} \frac{Y(t)-minY(t)}{\varepsilon }, & \begin{array}{cc} t-T_{trig}(tge0\;\vee \;Y(t)<minY(t)\\ 0, & Y(t)\geq minY(t) \end{array} \end{array}\right. \end{array}$$ (11)

$$\begin{array}{c} \frac{d\;maxY}{dt}=\left\{ \begin{array}{cc} \frac{Y(t)-maxY(t)}{\varepsilon }, & \begin{array}{cc} t-T_{trig}(tge0\;\vee \;Y(t)>maxY(t)\\ 0, & Y(t)\leq maxY(t) \end{array} \end{array}\right. \end{array}$$ (12)

Stroke Volume is computed via the integral operator in Eq. 13, which accumulates flow until the next trigger event and resets thereafter.


$$\begin{array}{c} \frac{d\;intY}{dt}=\left\{ \begin{array}{cc} -\frac{intY(t)}{\varepsilon } & t-T_{trig}(t)\geq 0\\ Y(t) & t-T_{trig}(t)<0 \end{array}\right. \end{array}$$ (13)

Because the quantity of interest corresponds to the value accumulated over the previous cardiac cycle, a supporting keeper equation (Eq. 14) is used to store the pre-reset value at each trigger event and hold it constant throughout the subsequent cycle


$$\begin{array}{c} \frac{d\;keepY}{dt}=\left\{ \begin{array}{cc} \frac{Y(t)-keepY}{\varepsilon } & t-T_{trig}(t)\geq 0\\ 0 & t-T_{trig}(t)<0 \end{array}\right. \end{array}$$ (14)

Finally, moving averages are computed continuously using Eq. 15, where the averaging period ($T_{avg}$) defines the temporal window of interest. Together, these equations enable online computation of cycle-based quantities required for real-time calibration and analysis.


$$\begin{array}{c} \frac{d\;avgY}{dt}=\frac{Y(t)}{T_{avg}}-\frac{avgY(t)}{T_{avg}} \end{array}$$ (15)

Here, *Y*(*t*) denotes an arbitrary scalar physiological variable of interest (e.g., pressure, flow, or volume), and *MinY*, *MaxY*, *IntY*, *AvgY*, and *KeepY* denote auxiliary state variables used to compute cycle-based statistics.

These operators allow cycle-resolved quantities to be computed online during integration, enabling direct comparison between simulated outputs and clinically defined target metrics used during model calibration.

#### 2.2.4. Final system of equations.

The complete system of ordinary differential equations is obtained by instantiating the generic model equations defined above for each cardiovascular compartment using the unified specification provided in Table 3. This table encodes, the compliance and elastance elements associated with each compartment, the resistive connections governing inter-compartmental flows, and the corresponding volume balance relationships, fully defining the network topology illustrated in Fig 2.

**Table 3:** Unified specification of the cardiovascular model. The table defines all compartmental compliance and elastance elements, inter-compartmental resistive connections, and volume balance relationships required to instantiate the full closed-loop system shown in Fig 2.

Capacitor	Eq.	Pressure	Volume	PBias
$C_{As}$	1	$P_{As}$	$V_{As}$	$P_{Atm}$
$C_{Cs}$	1	$P_{Cs}$	$V_{Cs}$	$P_{Atm}$
$C_{Vs}$	1	$P_{Vs}$	$V_{Vs}$	$P_{Atm}$
$C_{Vt}$	1	$P_{Vt}$	$V_{Vt}$	$P_{Atm}$
$C_{Ap}$	1	$P_{Ap}$	$V_{Ap}$	$P_{Atm}$
$C_{Cp}$	1	$P_{Cp}$	$V_{Cp}$	$P_{Atm}$
$C_{Vp}$	1	$P_{Vp}$	$V_{Vp}$	$P_{Atm}$
$C_{Hr}(1/{ℰ}_{Hr})$	1	$P_{Hr}$	$V_{Hr}$	$P_{Atm}$
$C_{Hl}(1/{ℰ}_{Hl})$	1	$P_{Hl}$	$V_{Hl}$	$P_{Atm}$
**Elastance**	**Eq.**	**Systole**	**Diastole**	** $T_{sys}/t_{tran}$ **
${ℰ}_{Hr}$	7, 10	$E_{Hr}$	$e_{Hr}$	HC·0.45/ HC·0.05
${ℰ}_{Hl}$	7, 10	$E_{Hl}$	$e_{Hl}$	
**Resistor**	**Eq.**	**Flow**	**Pin**	**Pout**
$R_{Vt\_Hr}$	4	$Q_{Vt\_Hr}$	$P_{Vt}$	$P_{Hr}$
$R_{Hr\_Ap}$	4	$Q_{Hr\_Ap}$	$P_{Hr}$	$P_{Ap}$
$R_{Ap\_Cp}$	2	$Q_{Ap\_Cp}$	$P_{Ap}$	$P_{Cp}$
$R_{Cp\_Vp}$	2	$Q_{Cp\_Vp}$	$P_{Cp}$	$P_{Vp}$
$R_{Vp\_Hl}$	4	$Q_{Vp\_Hl}$	$P_{Vp}$	$P_{Hl}$
$R_{Hl\_As}$	4	$Q_{Hl\_As}$	$P_{Hl}$	$P_{As}$
$R_{As\_Cs}$	2	$Q_{As\_Cs}$	$P_{As}$	$P_{Cs}$
$R_{Cs\_Vs}$	2	$Q_{Cs\_Vs}$	$P_{Cs}$	$P_{Vs}$
$R_{Vs\_Vt}$	2	$Q_{Vs\_Vt}$	$P_{Vs}$	$P_{Vt}$
**Compart.**	**Eq.**	**Qin**	**Qout**	**dV /dt**
$V_{As}$	3	$Q_{Hl\_As}$	$Q_{As\_Cs}$	$dV_{As}/dt$
$V_{Cs}$	3	$Q_{As\_Cs}$	$Q_{Cs\_Vs}$	$dV_{Cs}/dt$
$V_{Vs}$	3	$Q_{Cs\_Vs}$	$Q_{Vs\_Vt}$	$dV_{Vs}/dt$
$V_{Vt}$	3	$Q_{Vs\_Vt}$	$Q_{Vt\_Hr}$	$dV_{Vt}/dt$
$V_{Hr}$	3	$Q_{Vt\_Hr}$	$Q_{Hr\_Ap}$	$dV_{Hr}/dt$
$V_{Ap}$	3	$Q_{Hr\_Ap}$	$Q_{Ap\_Cp}$	$dV_{Ap}/dt$
$V_{Cp}$	3	$Q_{Ap\_Cp}$	$Q_{Cp\_Vp}$	$dV_{Cp}/dt$
$V_{Vp}$	3	$Q_{Cp\_Vp}$	$Q_{Vp\_Hl}$	$dV_{Vp}/dt$
$V_{Hl}$	3	$Q_{Vp\_Hl}$	$Q_{Hl\_As}$	$dV_{Hl}/dt$

For each compartment, the pressure/volume relationship is constructed from Eq. 1, with heart chambers additionally governed by the time-varying elastance formulation in Eq. 7. Inter-compartmental flows are generated from the specified resistive links using either the linear resistance formulation (Eq. 2) or the diode model (Eq. 4), and compartmental volumes evolve according to mass conservation as expressed in Eq. 3. Together, these instantiated equations yield a closed-loop cardiovascular system with coupled pressure, volume, and flow dynamics.

This formulation results in a system with 27 primary state variables, comprising 9 compartmental pressures, 9 compartmental volumes, and 9 inter-compartmental flow variables. The model includes 29 physiological parameters, consisting of resistances, compliances (or elastances), and unstressed volumes (*R*, *C*/*E*, *V*_0_). Two additional parameters arise from the heart compartments, which are characterised by maximum and minimum elastances rather than fixed compliances.

### 2.3. Model calibration

Calibrating the cardiovascular model to the population requires identifying parameter values that reproduce the characteristic pressures, volumes and flows of that population while respecting the intrinsic relationships encoded in the governing equations. Because each variable is influenced simultaneously by all compliances, resistances and volumes, changes in any parameter propagate throughout the entire system. This coupling makes direct parameter assignment difficult and undermines traditional optimisation methods, which adjust parameters independently and ignore the monotonic interactions built into the model. By operating outside the model’s dynamical structure, these approaches attempt to match target pressures or flows without using the natural correlations encoded in the equations. Because several parameters can influence the same variable in similar ways, they cannot reliably determine which adjustments are appropriate, often require extensive computational search to find acceptable solutions, and frequently return multiple distinct parameter sets that satisfy the same targets. As the number of variables increases, these methods scale poorly, becoming increasingly resource intensive.

To address these issues, we introduce an embedded calibration strategy entitled Embedded Feedback Controller (EFC) described in Section 2.3.1. Here, rather than keeping the calibration parameters as fixed constants, we add dedicated differential equations that govern their evolution, each constructed according to the qualitative correlations between parameters and variables. These new equations operate alongside the physiological ODEs and drive the parameters in the direction required to reduce their respective errors, continuously ‘nudging’ the system toward the prescribed targets.

Because the EFC framework introduces parameter dynamics directly into the model, the corresponding correction equations must be tailored to the physiology and cannot be assigned arbitrarily. Section 2.3.2 explains how these equations were selected by leveraging the structure of the governing ODEs, the qualitative correlations between parameters and variables, and established physiological principles. This stands in contrast to traditional plug-and-play optimisation methods, which adjust parameters without regard for how those adjustments align with the underlying system behaviour.

An overview of the EFC calibration framework is shown in Fig 5. The diagram summarises how clinical observables are mapped onto model variables within the cardiovascular network, how the governing physiological equations and embedded controllers interact and how parameter adaptation is driven by target errors to steer the system toward a physiologically consistent steady state. The formal definition of the calibration dynamics is introduced in the following subsection.

**Fig 5:**
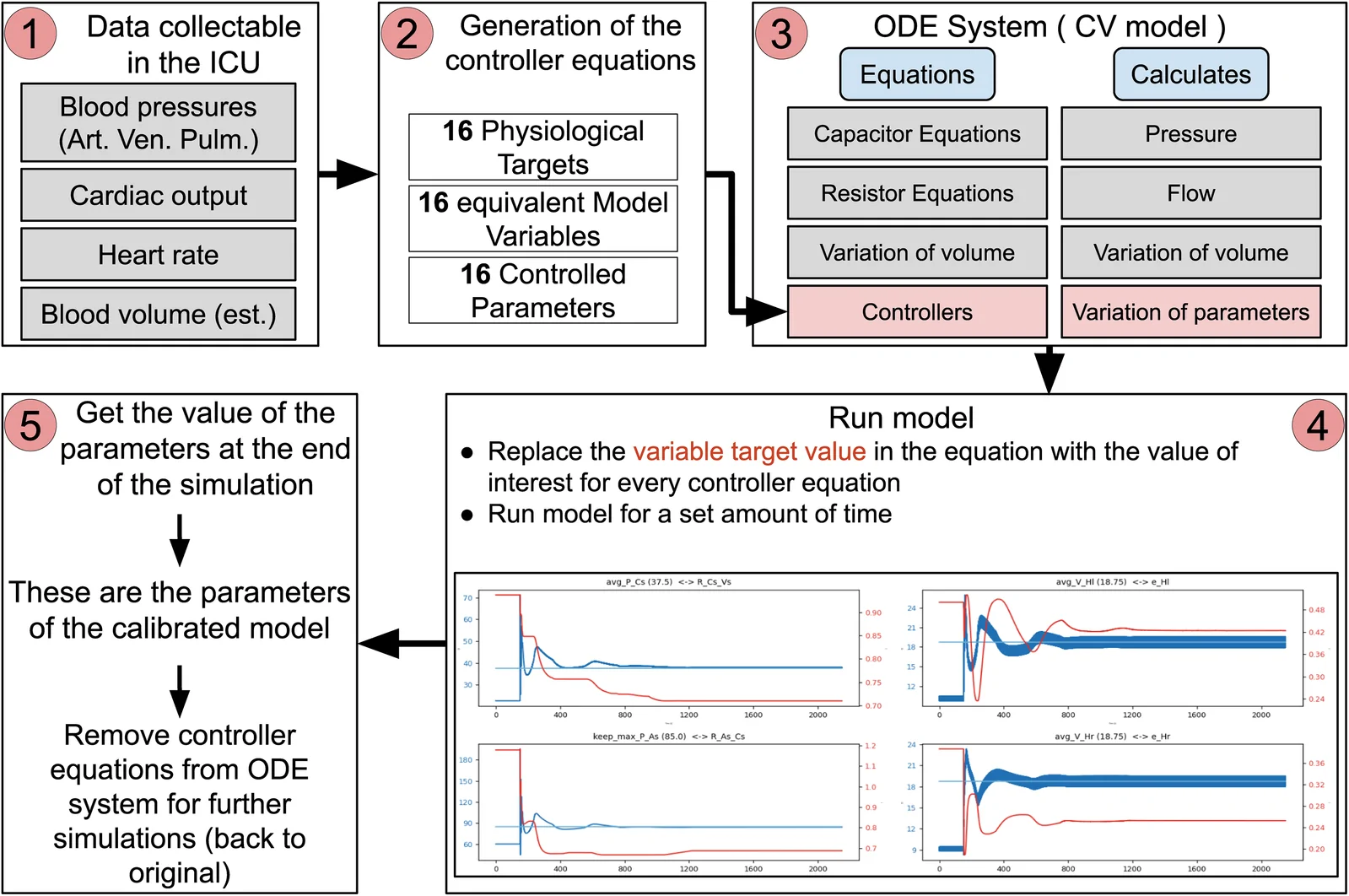
Workflow of the calibration methodology using Embedded Feedback Controller (EFC). (1) Physiological quantities that can be measured in the ICU are the calibration targets. (2) These clinical targets are mapped onto the corresponding model variables and the parameters that influence them, establishing the link between observable data and the internal states of the cardiovascular model. (3) The full model is then assembled by combining the governing physiological equations (capacitor, resistor, and volume-balance relations) with the EFC controller equations that prescribe how selected parameters evolve in response to their relative errors. (4) The model is run with all controllers active, allowing pressures, flows, volumes, and parameters to co-evolve until the system converges toward the prescribed target state. (5) Once equilibrium is reached, the resulting parameter set is extracted, providing a calibrated, physiologically consistent representation of the patient or target population.

#### 2.3.1. Embedded feedback controller.

Careful observation of eq. 1–3 allows us to extract the correlation between all the parameters and variables, as shown in Table 4. These correlations describe monotonic qualitative effects and do not imply linearity or exclusivity, but they are sufficient to define the sign of each calibration controller.

**Table 4:** Qualitative Correlation Matrix for a Generic Compartment where the model variables of Pressure, Volume and Flow are compared to the parameters for Compliance, Resistance and unstressed volume (*V*_0_).

	*C*	*R*_in_	*R*_out_	*V*_0_
*P*	–	–	+	–
*V*	+	–	+	+
*Q*	0	–	–	0

This table tells us that the task of increasing pressure in a compartment can be done either via increasing the outlet resistances ($R_{out}$) or decreasing the inlet resistances ($R_{in}$), compliance (*C*) or unstressed volumes (*V*_0_). Volume can be increased by increasing (*C*), $R_{out}$ and *V*_0_ or decreasing $R_{in}$. Reducing all resistances will increase the flow through the system and decreasing the compliance will also indirectly increase flow through as less fluid is retained in the compartment.

Knowledge of these relations between the parameters and the variables can be leveraged to create an extra set of differential equations that will drive the model to settle into physiologically relevant zones where the values for pressure, volume and flow generated by the model can be actively targeted. This can be done by reinterpreting the model parameters from constants, i.e., ‘literals’, to model states with derivative $\frac{dY}{dt}=0$. Any parameter that needs calibration so that the model displays a certain value for a variable (target value) has its derivative function ($\frac{dY}{dt}=0$) replaced with another monotonic function that would respect the correlations of Table 4. In this work, parameter adaptation is implemented using a saturated polynomial law combining a cubic and a linear term,


$$\begin{array}{c} \frac{dY}{dt}=(a_{C}\;\sigma_{rel}^{3}+a_{L}\;\sigma_{rel},Y_{\max},\;\sigma_{rel}=\frac{X^{⋆}-X(t)}{\left|X(t)\right|}, \end{array}$$ (16)

where *X*(*t*) is the current value of the model output being calibrated, $X^{⋆}$ its target, *Y* the parameter assigned to that target, and $Y_{\max}$ its upper bound, which rescales the dimensionless correction into parameter units, with $\sigma_{rel}$ saturated at ±1. The gains $a_{C}$ and $a_{L}$are signed: their common sign encodes the qualitative parameter/variable correlation of Table 4, so that a positive error drives *Y* in the direction that reduces it. Setting the gain of either side to zero recovers a pure law (cubic or linear). In Section 3.1 we compare the effects of the cubic law, $a_{L}=0$, and the linear law, $a_{C}=0$. The populations reported in the remainder of this work were calibrated with the linear law. Even powers such as $\sigma_{rel}^{2}$are excluded because they are not monotonic in the error: the correction keeps the same sign on both sides of the target. Each parameter is held inside its operational range $\left[Y_{\min},Y_{\max}\right]$: whenever *Y* leaves the interval, its derivative is replaced by a constant term directed back into it.

Two properties follow directly from Eq. 16 and characterise the dynamics of a single controller. First, *dY*/*dt* is zero only when the error is zero. When a target is infeasible the parameter is instead held against a bound. Second, the rate at which the target is approached depends on which term is active: under the linear law the Jacobian is non-zero, $\dot{\sigma }rel\propto -\sigma rel$, and the residual decays exponentially at a rate set by $a_{L}$. Under the cubic law the Jacobian vanishes, linearisation carries no information about the approach, and $\dot{\sigma }rel\propto -\sigma {rel}^{3}$ decays as *t*^–1/2^. Both laws are run with a staged schedule of increasing gains, raised once under the linear law and four times under the cubic (Appendix A.6).

#### 2.3.2. Construction of the full calibration system.

We now extend the EFC strategy from the Generic Compartment to the full cardiopulmonary circulation. Using the model of Fig 2 as a reference, our goal is to construct a complete set of controller equations that can calibrate the system to any admissible set of measured variables. The selection of these controllers is guided by three elements: the structure of the governing equations, the qualitative parameter/variable correlations summarised in Table 4, and basic physiological principles. The resulting mapping between targets and parameters is shown in Fig 6, organised into ten calibration groups.

**Fig 6:**
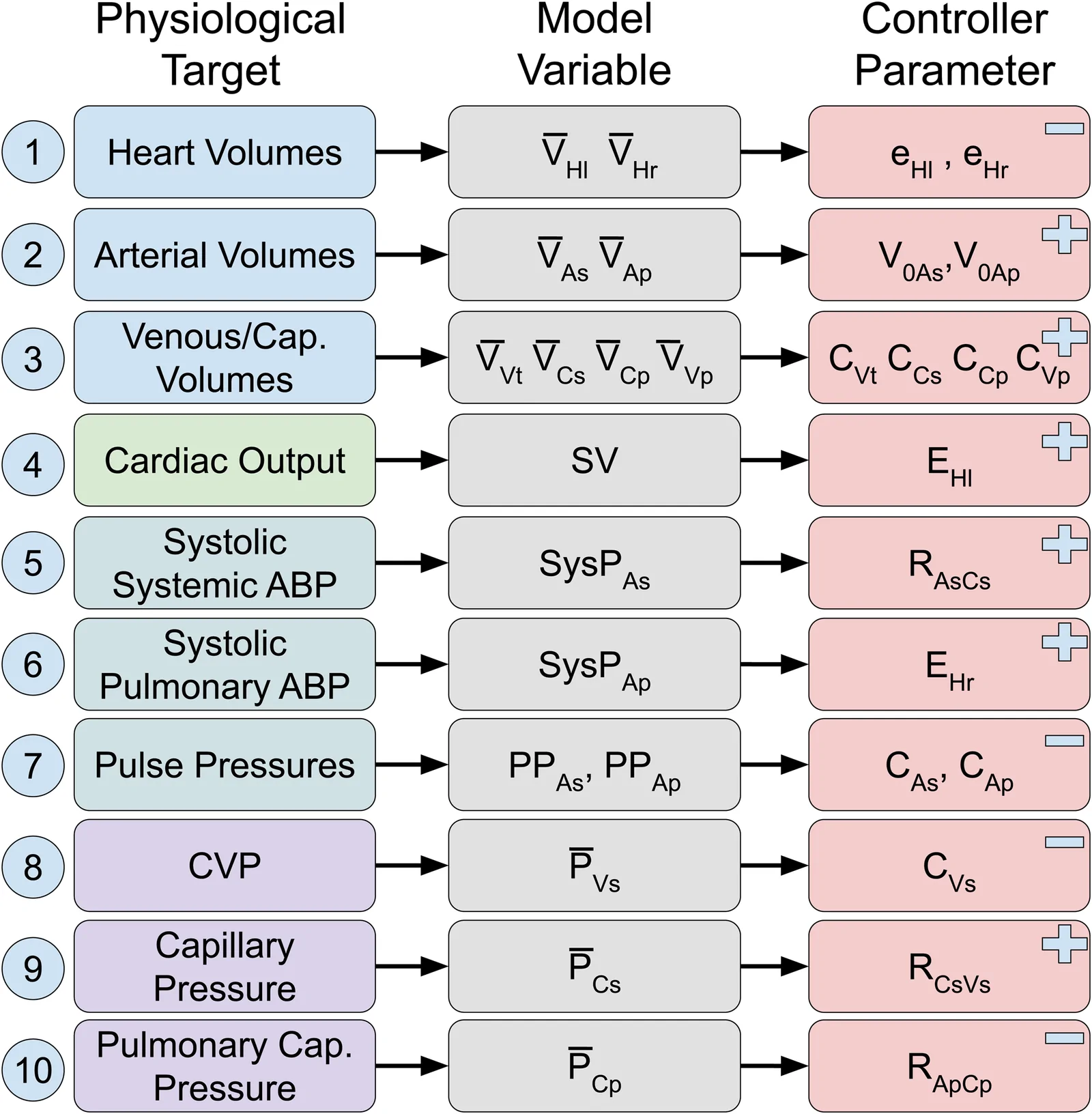
Overview of the parameter/target assignments used in the Embedded Feedback Controller calibration, 16 calibrated parameters against 16 imposed physiological targets, one controller per pair. Each row corresponds to a physiological quantity targeted during calibration (left), the associated model variable (centre) and the parameter adjusted by the controller (right). Symbols ‘+’ and ‘-’ denote positive and negative qualitative correlations between parameters and target variables, respectively, as defined in Table 4.

**Volume equations (groups 1–3)** Because TBV is fixed, only eight of the nine compartmental volumes listed in Table 1 must be actively targeted. Therefore, the systemic venous compartment is left uncontrolled so that it absorbs the residual volume required to satisfy conservation. For the heart chambers (group 1), the average diastolic volumes are controlled using the relaxed elastances $e_{Hl}$ and $e_{Hr}$: among the cardiac parameters, these exert the strongest and most selective influence on diastolic filling, with a clear negative correlation between elastance and volume. The arterial volumes (group 2) are regulated through their unstressed volumes *V*_0,*As*_ and *V*_0,*Ap*_, the only compartments for which $V_{0}\text{≠}0$. Because *V*_0_ shifts the pressure–volume relation in Eq. 1 without altering compliance, it provides a direct and isolated mechanism for adjusting the mean arterial volume. Venous and capillary volumes (group 3) are controlled through their compliances ($C_{Vt}$, $C_{Cs}$, $C_{Cp}$, $C_{Vp}$), which appear in Eq. 1 and exert a positive, dominant influence on stored volume in these highly compliant regions of the circulation. These parameter choices ensure that each targeted volume is adjusted through the variable with the most physiologically direct and least confounded effect on that compartment.

**Cardiac output equation (group 4)** Stroke volume, and thus cardiac output (CO=SV·HR with HR provided as a model input), is controlled through the left ventricular contraction elastance $E_{Hl}$. Because this parameter determines the amplitude of ventricular emptying, it provides a direct way to reach the target stroke volume while remaining consistent with the overall assumption of uniform average flow throughout the system.

**Arterial pressures and pulse pressures (groups 5–7)** Systemic systolic pressure (group 5) is regulated with the systemic outlet resistance $R_{AsCs}$, as the inlet resistance is assumed small and fixed. Once systolic pressure is set, the corresponding pulse pressure (group 7) is shaped by the systemic arterial compliance $C_{As}$, whose negative correlation with pressure amplitude makes it well suited for this role. The same logic is applied symmetrically in the pulmonary circulation: the right ventricular contraction elastance $E_{Hr}$ determines pulmonary systolic pressure (group 6), while pulmonary arterial compliance $C_{Ap}$ is used to adjust the pulmonary pulse pressure (group 7).

**Venous and capillary pressures (groups 8–10)** Central venous pressure (group 8) is controlled through the systemic venous compliance $C_{Vs}$, which modulates the pressure/volume relation in this compartment. Systemic capillary pressure (group 9) is influenced most strongly by the downstream resistance $R_{CsVs}$ and is therefore calibrated using this parameter. In the pulmonary capillaries (group 10), the inlet resistance $R_{ApCp}$ typically dominates the local pressure drop, making it the appropriate choice for controlling $P_{Cp}$.

**Combined effect of all controllers.** This assignment yields a set of 16 controller equations corresponding to the 16 extended targets in Fig 6. Each target is paired with the parameter that most directly and selectively influences it, following the qualitative correlations of Table 4. Two regions of the circulation, the thoracic veins ($P_{Vt}$) and pulmonary veins ($P_{Vp}$), are intentionally left uncontrolled. Although resistances connect these compartments to their neighbours, these venous resistors, together with all cardiac resistors, are assumed to be very small, producing negligible pressure drops and modifying them would therefore have minimal physiological effect.

Every parameter left uncontrolled is a resistance: the two venous resistors $R_{Cp\_Vp}$ and $R_{Vs\_Vt}$ and the four valve resistances. Every compliance, elastance and unstressed volume of the model is calibrated. The only effect of a resistance is the pressure drop ΔP=QR, so the pressure of each of these two compartments follows from the flow through the resistor and the pressure on its other side, both of which are targeted. Either compartment can be brought into the calibration by adding its resistor as a controller and its pressure drop as a target.

When the full set of controllers is active, all 16 equations operate simultaneously, with each correction altering pressures and flows throughout the network. The resulting interactions cause the controllers to continually adjust one another, guiding the system toward the configuration in which all relative errors are minimised. The problem is nevertheless over-determined, because each parameter influences several compartments and the calibrated state must additionally satisfy the intrinsic conservation and closure relations of the closed-loop ODE system. The cardiac cycle period is likewise excluded from the targeted set, being an input prescribed by the heart rate, and the remaining model parameters, including the venous and valve resistances, are held at fixed literature values.

#### 2.3.3. Construction of calibration targets from model outputs.

Clinical observables and derived haemodynamic targets (e.g., systolic pressure, pulse pressure, stroke volume, and mean compartmental volumes) are defined over cardiac cycles rather than at instantaneous time points. To enable direct comparison between continuous model states and these cycle-resolved targets, the auxiliary operators introduced in Section 2.2.3 are designed to extract clinically meaningful quantities online during numerical integration.

Stroke volume is computed as the value of the cycle-integrated aortic flow retained from the previous heartbeat,


$$\begin{array}{c} SV=keep\;\left(int(Q_{HlAs})\right), \end{array}$$ (17)

and cardiac output is then obtained from *SV* and heart rate as CO=SV·HR (with consistent unit conversion where required). Systolic arterial pressure is extracted as the retained cycle maximum,


$$\begin{array}{c} SysP_{*}=keep\;\left(max(P_{*})\right), \end{array}$$ (18)

while pulse pressure is computed from the retained cycle extrema,


$$\begin{array}{c} PP_{*}=keep\;\left(max(P_{*})\right)-keep\;\left(min(P_{*})\right). \end{array}$$ (19)

Compartmental volume targets are formulated using cycle-averaged values,


$$\begin{array}{c} \overset{―}{V_{*}}=avg(V_{*}). \end{array}$$ (20)

The complete set of operator compositions used to construct the calibration targets, together with the corresponding model variables and the parameters adapted by each controller, is summarised in Fig 6. This mapping defines the interface between clinical targets and the internal model dynamics within the Embedded Feedback Controller framework.

### 2.4. Numerical implementation details

All differential equations defining the cardiovascular model, cycle-based operators, and calibration dynamics were integrated using an explicit Euler scheme with a fixed time step Δt. This formulation was selected to allow precise control over event-like behaviour, such as cardiac cycle triggering and operator resets, which are naturally expressed within a time-stepped framework.

Discrete events, including cardiac cycle triggers and the reinitialisation of cycle-based operators (e.g., maxima, minima, and integrals), were implemented as fast relaxations over an infinitesimal reset time ε rather than as explicit hybrid events. This approach avoids discontinuities in the state variables while preserving the intended cycle-level behaviour within a purely ODE-based formulation.

The integration time step was set to Δt=0.0005s, which was sufficient to resolve the fastest dynamics in the system, including elastance transitions and calibration updates, while maintaining computational efficiency and numerical stability.

Appendix A.3 reports the same staged calibration repeated with a fixed-step fourth-order Runge-Kutta method and with the adaptive Dopri5 (an RK4(5) pair with adaptive time stepping). All three schemes reach the same calibrated parameters and the same residual error, and Euler arrives at the solution 4.4 times faster than RK4 and 87 times faster than Dopri5. One calibration is one simulation, so a single virtual subject is calibrated in approximately 50 s of wall-clock time on a single workstation CPU.

Parameter bounds were introduced in the controller equations solely as numerical safeguards to prevent divergence. These bounds were intentionally chosen to be substantially wider than physiologically plausible ranges and therefore do not constrain the solution space in a meaningful way. A calibration whose targets the model can meet comes to rest with every parameter inside the controller bounds. A parameter that reaches a bound has run past every physiologically plausible value without meeting its target, so that combination of targets is infeasible for the model.

### 2.5. Simulation setup

To assess whether the calibration framework converges to the same solution independently of initial conditions, 1024 simulations were performed in which model parameters were initialised randomly over the prior box by Latin hypercube sampling and TBV was distributed randomly across compartments. Target values corresponded to the average normal-physiology subject defined in Table 6 and were held fixed across the population, so that the spread over the runs measures the reproducibility of the calibration alone. Each simulation opens with a settling stage in which the controllers are inactive and continues through a staged schedule of increasing gains. Both schedules are given in Appendix A.6. The population was calibrated twice, once under each of the two control laws of Eq. 16, with every other setting held identical. One calibration is one simulation, so its cost is the length of that simulation, expressed throughout as a count of 10s forward solves and timed in Appendix A.3.

The same task was also given to two independent and widely used methods, affine-invariant ensemble Markov chain Monte Carlo (MCMC, Appendix A.4) and multi-start gradient descent (GD, Appendix A.5), each described there together with the measures its implementation required on this model. All four calibrations drive the same 16 parameters to the same 16 targets over the same prior box, under the same explicit Euler integration at Δt=0.0005s. For MCMC and GD the controllers are disabled, with both update gains set to zero, so each objective evaluation reduces to a plain forward solve of the uncontrolled model and the sampled parameters enter only through the initial state. Both then minimise, or sample from, the same weighted residual over the 16 targets,


$$\begin{array}{c} J(\theta )=\sum_{i=1}^{16}{\left(\frac{o_{i}(\theta )-t_{i}}{\sigma_{i}}\right)}^{\;2},\;\sigma_{i}=\varepsilon \;|t_{i}|, \end{array}$$ (21)

under a uniform prior over the box and a Gaussian likelihood proportional to exp(−J/2). The two runs used different residual scales ε, so their values of *J* are not comparable with one another, and only relative error and computational cost are compared anywhere in this work.

To evaluate the ability of the framework to generate heterogeneous yet physiologically consistent populations, three cohorts were constructed corresponding to normal physiology, warm shock (WS), and cold shock (CS). For each cohort, admissible target ranges were defined according to Table 2. A Latin hypercube sampling strategy was used to generate 200 independent target sets per cohort. Each target set was then used to calibrate the full cardiovascular model using the Embedded Feedback Controller procedure, yielding one virtual subject per sample. The same simulation protocol and timing configuration were used for all cohorts.

## 3. Results

### 3.1. Convergence and method benchmarking

**Cost.** The same calibration task was solved under both control laws as well as MCMC and GD, configured as described in Section 2.5. Table 5 reports the cost and the accuracy of each method. Here one solve/iteration is considered 10s model simulation taking 0.134s of wall-clock time (370*0.134=49.6s for a full linear model calibration procedure).

**Table 5:** Cost and accuracy of the four calibrations on the identical task of Section 2.5, every entry counted in one unit, a single ten-second forward solve timed in Table 8. *Sample size* is each method’s starts, walkers or restarts; the rows below *Best* describe the spread over them.

	EFC (linear)	EFC (cubic)	MCMC	GD
Sample size	1024	997	128	64
Solves / step	1	1	1	33
Steps	370	670	1 500	200
Solves/cal	370	670	192 000	6 601
Total solves	378 880	686 080	193 418	424 297
Best $|\varepsilon_{r}{|}_{\max}$%	0.92	0.90	0.37	0.04
Median $|\varepsilon_{r}{|}_{\max}$%	0.95	1.09	0.92	11.54
Samples <0.5 %	0	0	2	30
Samples <1 %	100	44	61	38
Samples <2 %	100	100	100	45
Samples <5 %	100	100	100	48

**Ensemble sizes:** The sample size is the number of members each method carries, all four seeded by Latin hypercube over the same prior box: 1024 and 997 starts for the linear and cubic laws, 128 walkers for MCMC, and 64 restarts for GD screened from a 1024-point pool. The cubic law was run with 1024 starts, of which 27 diverged during integration. One step of one member costs a single forward solve under both control laws and for MCMC, since each advances its state by integrating the model once. GD costs 33 solves per step, one for the objective and 32 for the central finite-difference stencil over the 16 parameters, so 32 of every 33 solves are spent measuring a gradient.

**Solves per calibration:** The steps row gives the number of iterations each method takes, with total solves being the amount of 10s blocks needed to run all the samples through all the algorithm’s steps. Because in the EFC the controller equations are responsible for the calibration, we only need a single forward pass per sample. On the GD each iteration uses 33 solves, so a full calibration for one sample needs (33*200 + 1 = 6601 iterations). On the MCMC we always needed a population of samples to get a posterior distribution. This is why the MCMC entry sits close to its total of 193 418, the difference being the 1 418 evaluations performed outside the chain: the 512-point warm-up cloud that defines the whitening map, the 128 initial ensemble states and the 778 burn-in rescues of walkers stranded far from the ensemble.

**Accuracy:** GD has the lowest best error of the four at 0.04% and the linear law the highest at 0.92%. However the median GD restart misses by 11.54%, because 35 of its 64 restarts settle in a local minimum at a systemic arterial resistance of 0.375 against 0.744 for the restarts that reach the targets, and come to rest there with the worst target missed by up to 18% (Appendix A.5, Fig 17). The two control laws show no such gap. The linear law returns 0.92% at its best and 0.95% at its median across 1024 starts, and the cubic law 0.90% and 1.09%. Every start under the linear law reaches an error below 1%, against 44% of the cubic starts and 61% of the MCMC samples. GD holds the largest share at the tightest threshold, 30% of its restarts below 0.5% against 2% of the MCMC samples and none under either control law.

**Convergence behaviour:** Fig 7 compares the median error across all samples of each method against the normalised step numbers. Here we can see that both the linear EFC and the MCMC converged to the final solution before the end of the simulation, with the linear EFC converging at roughly 60% of the step length (222 steps) and the MCMC at 50% (750 steps). Under both control laws the error oscillates around the target, spanning 0.47% over the final fifth of the run under the linear law and 0.26% under the cubic. The cubic law is slower to arrive and settles further from the target, falling below 1% at step 512 of its 670 against step 193 of 370 under the linear law. This is because the Jacobian of the pure cubic law is proportional to $3a_{C}\sigma_{rel}^{2}$ and vanishes at the target, so its correction weakens as the residual shrinks.

**Fig 7:**
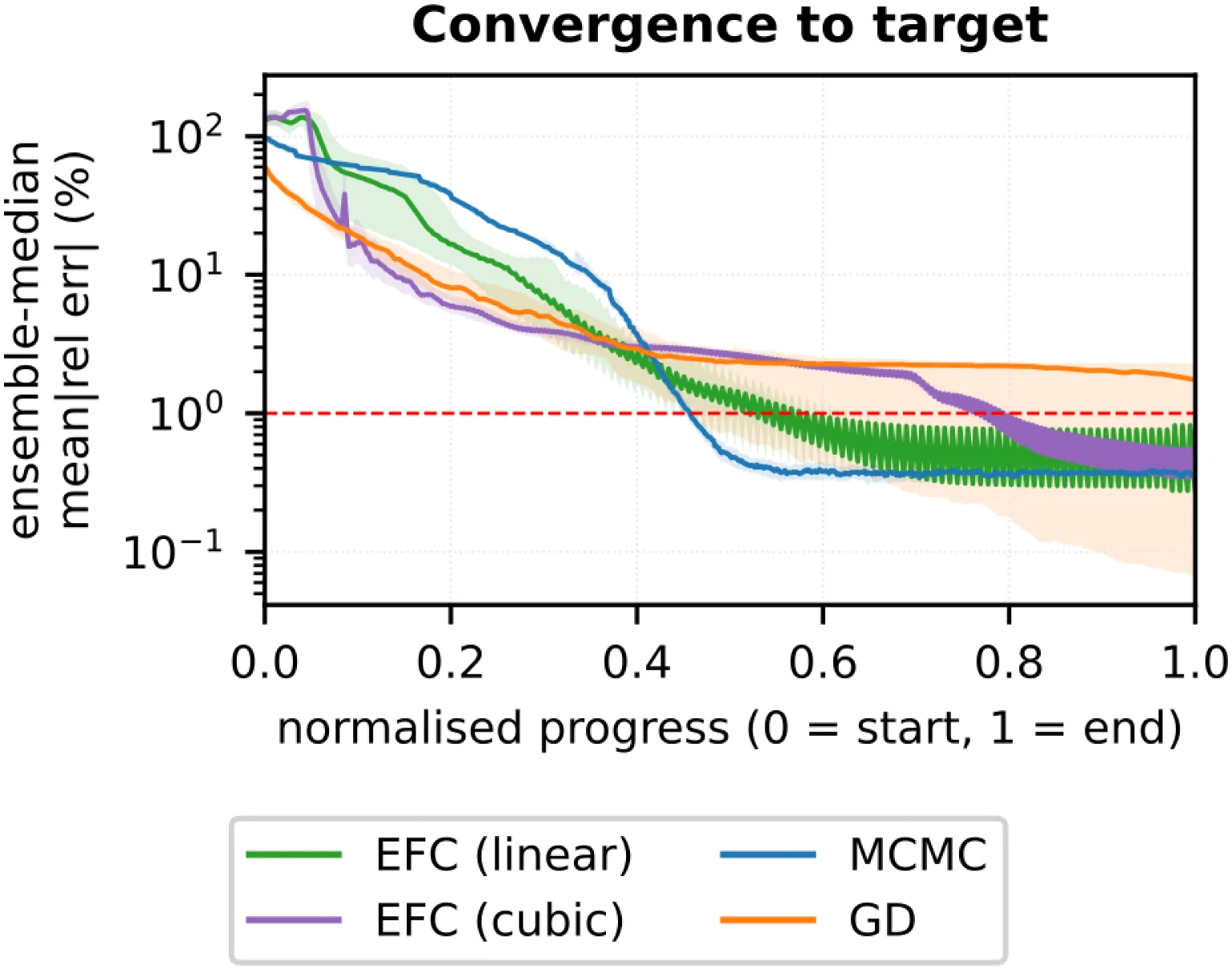
Ensemble median of the mean relative error over the 16 targets, against each method’s own progress normalised to the unit interval, with a band spanning the interquartile range over the attempts. The dashed red line is the 1% tolerance.

**Target agreement:** Fig 8 resolves the four populations target by target in panel (a) and parameter by parameter in panel (b). The median attempt of every method lands within 1% of every target, the largest miss being the 0.95% of the linear EFC on ${\overset{―}{V}}_{Cs}$. The spread around those medians separates the methods: the per-target interquartile width is 0.009% under the linear law, 0.14% under the cubic, 0.65% for MCMC and 0.49% for GD. GD also carries the tail, with 33 of its 64 restarts missing at least one target by more than 10% and the worst by 18%.

**Fig 8:**
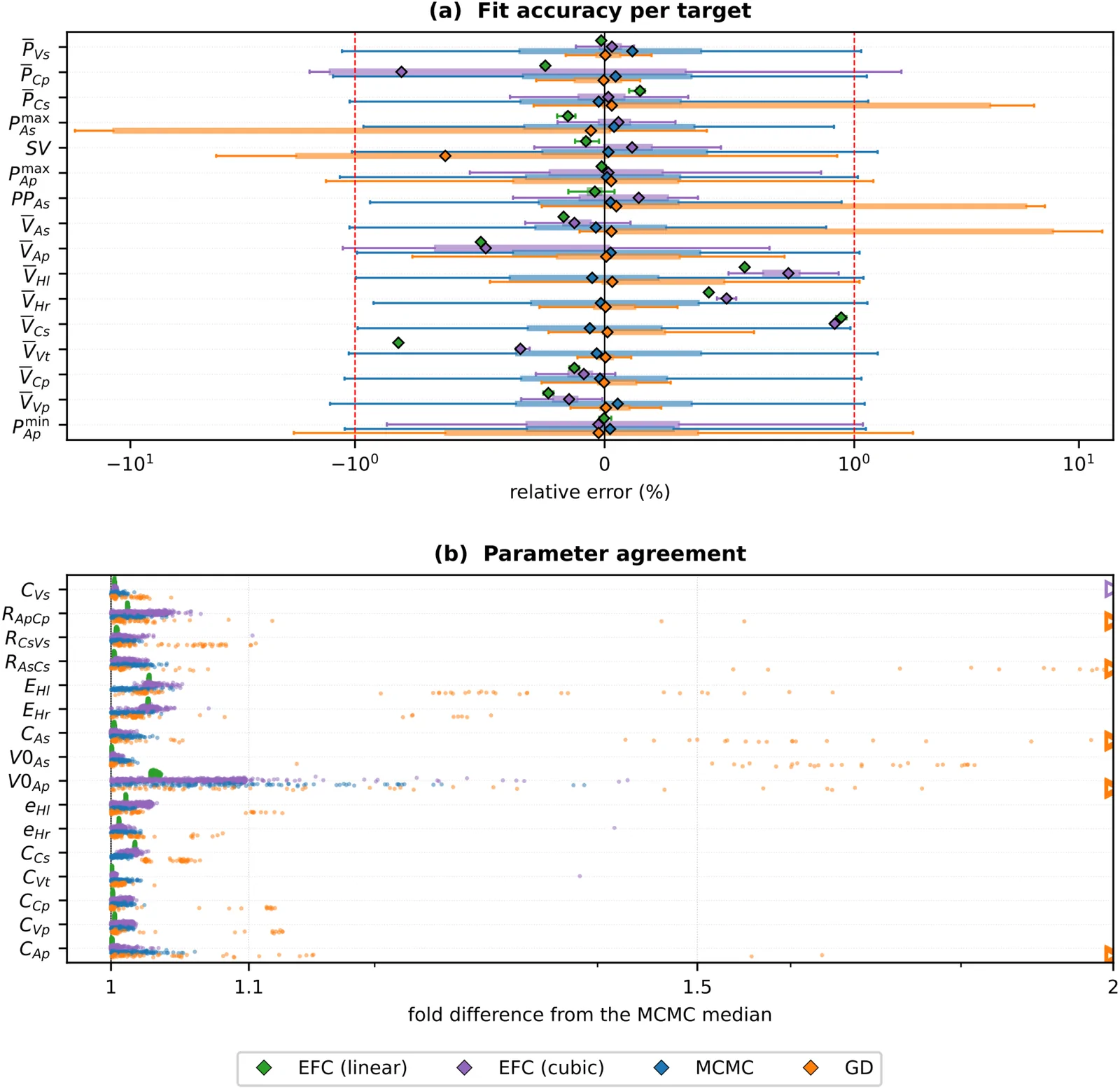
(a) Signed relative error of each of the 16 targets, one box per method, dashed red lines at ±1%. (b) Calibrated parameters, one point per attempt, as the ratio to the MCMC posterior median with values below one inverted, so 1 is exact agreement and 2 is a factor of two out in either direction. Points past the right of the axis are drawn as open markers on its edge.The diamond on each box is that method’s median. EFC (linear) has a per-target interquartile width of 0.009% against 0.65% for MCMC.

**Parameter agreement:** Panel (b) measures every attempt against the MCMC posterior median. All 16 parameters of all 1024 linear-law starts sit within 1.1 times that reference, as do 99.8% of the cubic values, 98.2% of the MCMC walkers and 80.5% of GD’s, whose misses are the restarts stalled in local minima. Half of the linear population’s parameter values lie within a fold difference of 1.003 of the reference, and none exceeds 1.035 times the MCMC median value for the parameter. The largest is the unstressed pulmonary arterial volume *V*_0,*Ap*_, whose 1024 values sit at 1.03. Half of the cubic values lie within 1.007 of the reference, and all fall below 1.43. The largest is again *V*_0,*Ap*_, whose values sit at 1.065.

**Reference method (linear):** The populations in the remainder of this work are calibrated with the linear law, at 370 solves per calibration. Table 6 summarises the results of the calibration procedure for the test using the linear controllers. Across all simulations, all targeted variables converged to their prescribed values with negligible signed relative error (below 1%, computed as $100\times (Y_{value}-Y_{target})/Y_{target}$). The agreement extends beyond the targets to the calibrated parameters themselves: taking the parameter standard deviations of Table 6 relative to their means, the coefficient of variation across the 1024 randomly initialised runs has a median of 0.016% and a maximum of 0.16%. The runs therefore converge to the same target values, and to the same point in parameter space. The largest spread occurs for *V*_0,*As*_ and *V*_0,*Ap*_.

**Table 6:** Summary of the convergence test across 1024 simulations with random initial parameter values and volume distributions. For each calibration target, the table reports the prescribed target value, the mean signed relative error and its standard deviation at convergence, together with the corresponding calibrated parameter values and their variability across runs.

Vars.	Targets	Relative Error %	std	Param.	Value	std
${\overset{―}{P}}_{Vs}$	8	-0.014	1e-3	$C_{Vs}$	59.997	3e-4
${\overset{―}{P}}_{Cp}$	11.5	-0.239	6e-3	$R_{ApCp}$	0.165	3e-5
${\overset{―}{P}}_{Cs}$	37.5	0.137	1e-2	$R_{CsVs}$	0.708	2e-4
$P_{As}^{\max}$	85	-0.148	1e-2	$R_{AsCs}$	0.740	1e-4
*SV*	19.23	-0.073	2e-2	$E_{Hl}$	16.965	4e-3
$P_{Ap}^{\max}$	22.5	-0.010	6e-3	$E_{Hr}$	3.917	9e-4
$PP_{As}$	35	-0.041	4e-2	$C_{As}$	0.311	8e-5
$PP_{Ap}$	9	-0.026	1e-3	$C_{Ap}$	1.181	3e-4
${\overset{―}{V}}_{As}$	97.5	-0.164	3e-3	$V0_{As}$	76.244	7e-3
${\overset{―}{V}}_{Ap}$	26.25	-0.497	4e-3	$V0_{Ap}$	4.522	7e-3
${\overset{―}{V}}_{Hl}$	15	0.561	3e-3	$e_{Hl}$	0.439	6e-5
${\overset{―}{V}}_{Hr}$	15	0.417	1e-3	$e_{Hr}$	0.257	3e-5
${\overset{―}{V}}_{Cs}$	37.5	0.948	8e-3	$C_{Cs}$	1.003	2e-4
${\overset{―}{V}}_{Vt}$	30	-0.826	1e-3	$C_{Vt}$	4.448	1e-4
${\overset{―}{V}}_{Cp}$	22.5	-0.121	8e-3	$C_{Cp}$	1.959	2e-4
${\overset{―}{V}}_{Vp}$	26.25	-0.226	8e-3	$C_{Vp}$	2.372	3e-4

### 3.2. Sepsis population

To evaluate the ability of the calibration framework to generate heterogeneous yet physiologically consistent populations, three cohorts were constructed corresponding to normal physiology, warm shock (WS), and cold shock (CS). For each cohort, admissible target ranges were defined according to Table 2. A Latin hypercube sampling strategy was used to generate 200 independent target sets per cohort. Each target set was then used to calibrate the full cardiovascular model using the EFC procedure, yielding one virtual subject per sample.

Fig 9 summarises the distribution of relative calibration errors across all targeted pressures, volumes, and cardiac output for the three cohorts. For the majority of variables, errors are tightly centred around zero and symmetrically distributed, showing that the calibration procedure reliably achieves the prescribed targets across a wide range of physiological conditions.

**Fig 9:**
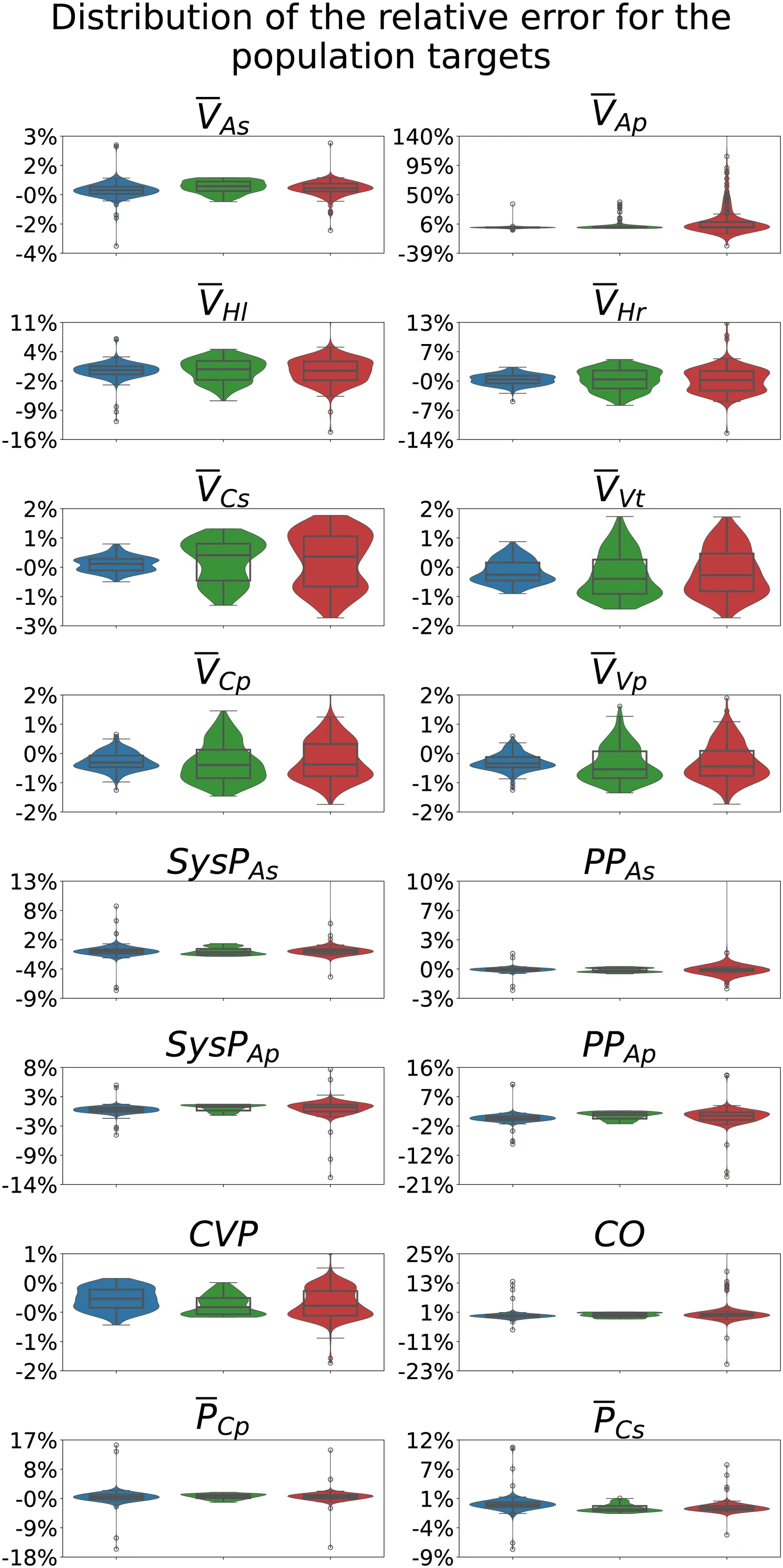
Distribution of relative calibration errors across all targeted physiological variables for the normal (green), WS (red), and CS (blue) populations. The same colour code is used for the cohorts throughout the paper. Errors are computed as relative deviations from the prescribed targets after convergence. Most variables exhibit errors tightly centred around zero, indicating robust calibration across cohorts.

The first pair of plots in Fig 10 focuses on the pulmonary arterial volume ${\overset{―}{V}}_{Ap}$, which exhibits the largest residual errors across the population. These errors are observed predominantly in the warm shock cohort. Importantly, the error is consistently positive, indicating a systematic tendency towards increased pulmonary arterial volume relative to the prescribed target. As shown in the corresponding parameter space, this behaviour coincides with saturation of the unstressed pulmonary arterial volume parameter *V*_0,*Ap*_ at its imposed lower bound. The second pair of plots highlights a similar saturation phenomenon affecting cardiac controllers. In this case, large residual errors are again associated with parameter saturation, but now lead predominantly to negative errors. In contrast, the third pair of plots shows a regime in which no controller saturation is observed and the error distribution is smoothly spread across the population.

**Fig 10:**
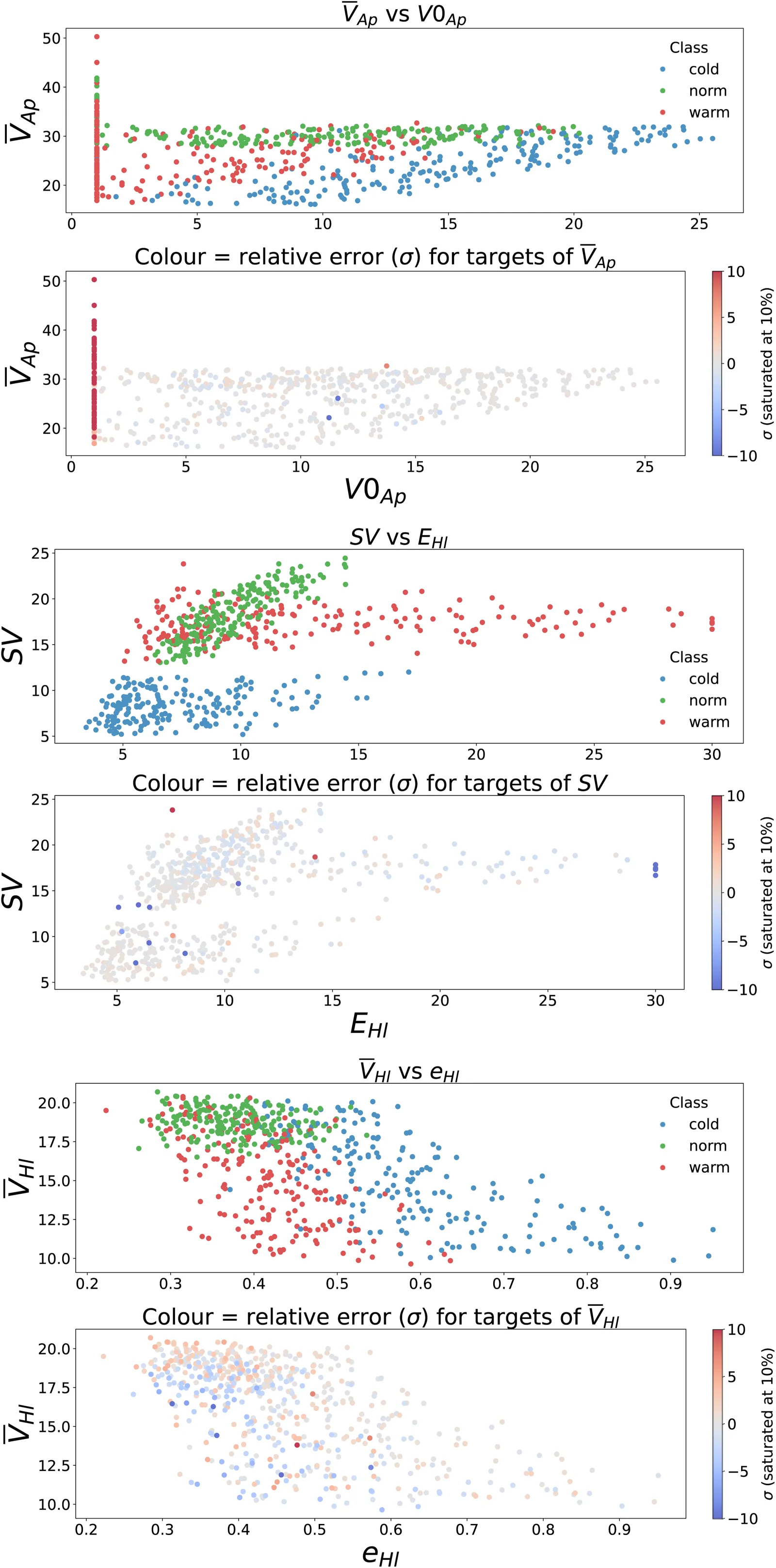
Relative calibration errors for representative population parameters across normal, warm shock, and cold shock cohorts. Upper panels show parameter values coloured by class; lower panels show the same samples coloured by relative error (saturated at 10%). Large residual errors coincide with controller saturation in specific regimes, while non-saturating controllers exhibit smoothly distributed errors driven by the cubic control law.

### 3.3. Population parameters

Fig 11 shows how global vascular and cardiac parameters organise across the three cohorts as functions of TBV and cardiac output. The top two panels show the distribution of total systemic resistance as a function of TBV and cardiac output. Across all cohorts, $R_{Total}$ exhibits a strong negative correlation with cardiac output and only a weak dependence on total blood volume.

**Fig 11:**
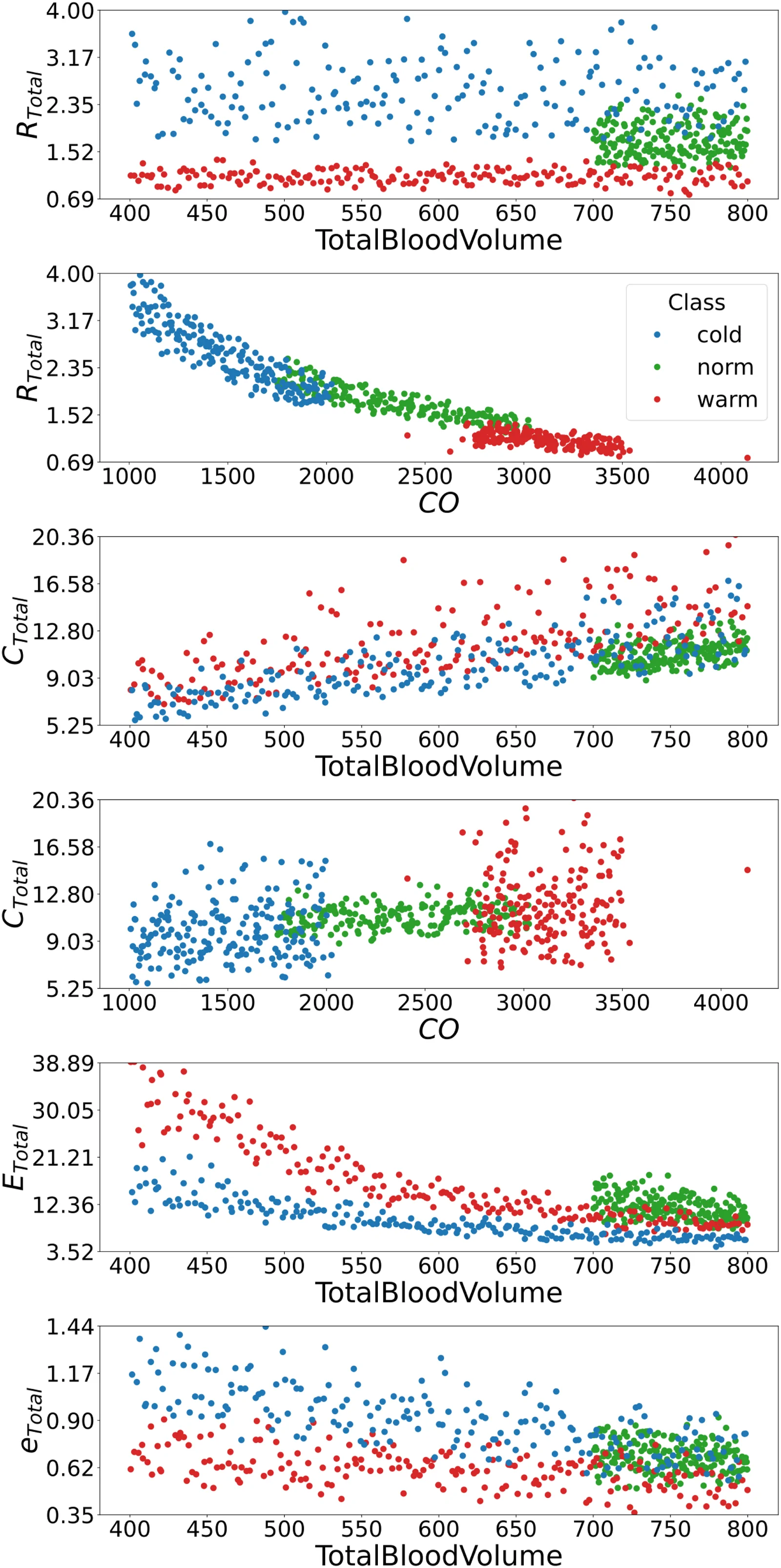
Distribution of total systemic resistance ($R_{Total}$), compliance ($C_{Total}$), and cardiac elastance ($E_{Total}$ and $e_{Total}$) across normal, warm shock, and cold shock populations as a function of total blood volume (*TBV*) and cardiac output (*CO*).

Despite this shared flow dependence, the cold shock population consistently occupies higher resistance regimes than normal physiology, while the warm shock population exhibits systematically lower resistance at comparable cardiac output and blood volume.

The third and fourth panels illustrate the total system compliance also as a function of TBV and cardiac output. Across all cohorts, $C_{Total}$ increases with total blood volume, whereas no clear dependence between $C_{Total}$ and cardiac output is observed. On average, the warm shock population occupies higher compliance regimes than the cold shock population, and for configurations with normal blood volumes, both septic populations show higher compliance than the normal cohort. Within the cold shock population at normal blood volumes, the total systolic elastance $E_{Total}$(5th panel) is markedly reduced, while the total diastolic elastance $e_{Total}$(6th panel) remains comparable to that of the normal population. Across the full volume range, $e_{Total}$ in cold shock is consistently higher than in warm shock, and in the low-volume regime both septic populations exhibit elevated diastolic elastance relative to normal physiology. Conversely, in the low-volume regime, the warm shock population exhibits substantially elevated systolic elastance relative to normal physiology.

Fig 12 illustrates the relationship between the arterial/capillary resistance ratio,


$$\begin{array}{c} \frac{R_{AsCs}}{R_{AsCs}+R_{CsVs}}, \end{array}$$ (22)

**Fig 12:**
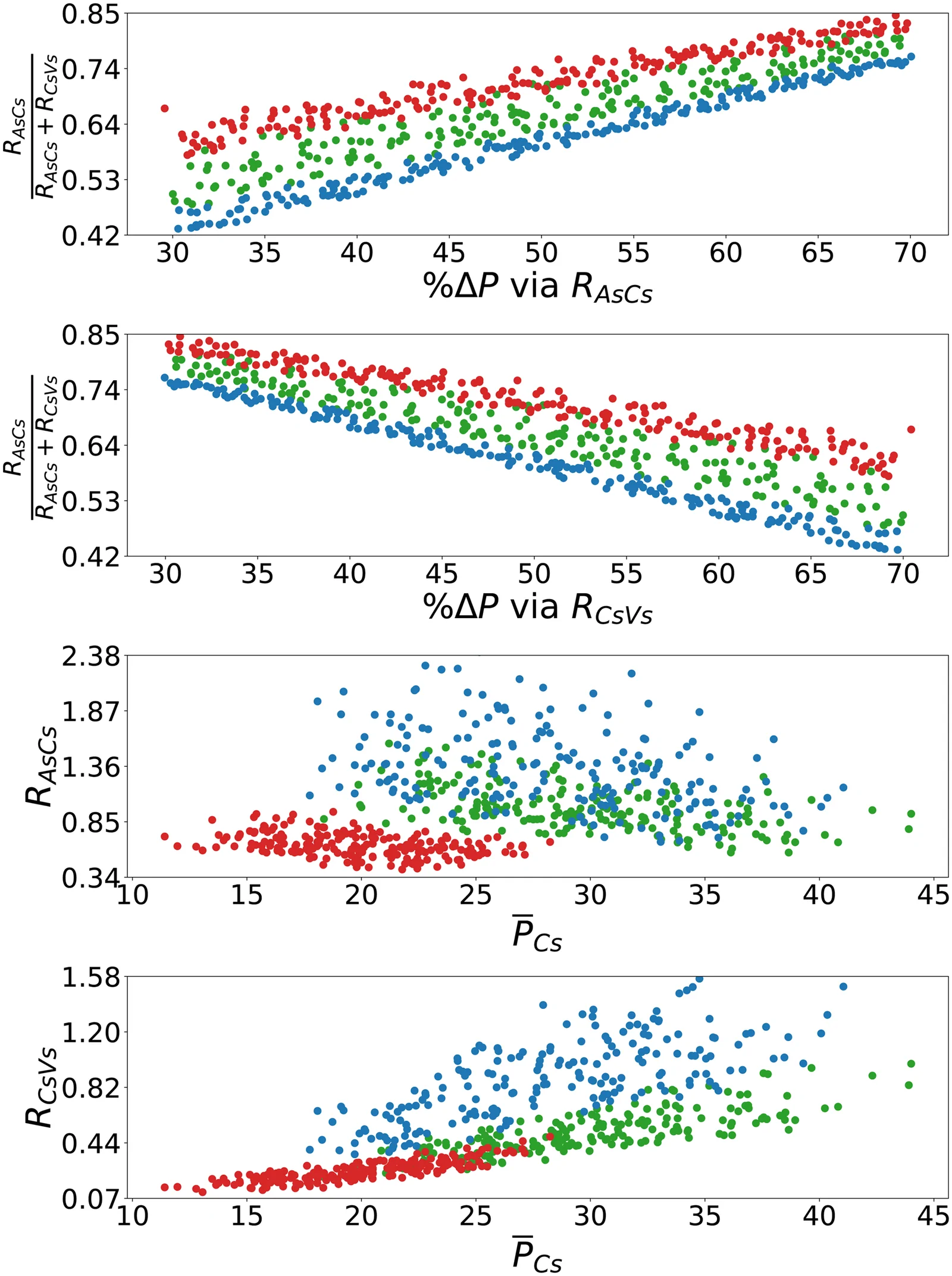
Top: Relationship between the arterial–capillary resistance ratio and the fractional pressure drop across arterial and venous compartments for normal, warm shock, and cold shock populations. Bottom: Scatter plots between the arterial resistance ($R_{AsCs}$) and the venous resistance ($R_{CsVs}$) and the average pressure of the capillaries (${\overset{―}{P}}_{Cs}$).

and the fractional pressure drop occurring across the arterial segment,


$$\begin{array}{c} \%\Delta P_{AsCs}=100\frac{DiaP_{As}-P_{Cs}}{DiaP_{As}-P_{Vs}}, \end{array}$$ (23)

as well as across the venous segment,


$$\begin{array}{c} \%\Delta P_{CsVs}=100\frac{P_{Cs}-P_{Vs}}{P_{As}-P_{Vs}}. \end{array}$$ (24)

The top panel shows that higher values of the resistance ratio correspond to a greater fraction of the pressure drop occurring between the arteries and the capillaries. Conversely, the second panel demonstrates complementary behaviour on the venous side, where increasing resistance ratios lead to a reduced fraction of the total pressure drop occurring between the capillaries and the veins. In the present results, the resistance ratio for the warm shock population is systematically higher than that of the cold shock population for comparable fractional pressure drops. The third and fourth panels relate the individual inlet and outlet resistances to the mean capillary pressure. In the third panel, $R_{AsCs}$ shows only a weak association with ${\overset{―}{P}}_{Cs}$: the warm shock population occupies a narrow, low- $R_{AsCs}$ band at lower ${\overset{―}{P}}_{Cs}$, whereas cold shock spans a much wider resistance range with a tendency for higher ${\overset{―}{P}}_{Cs}$ to coincide with lower $R_{AsCs}$.

In contrast, the fourth panel shows a clear monotonic increase of venous resistance $R_{CsVs}$ with ${\overset{―}{P}}_{Cs}$across all cohorts, with the largest values occurring in cold shock. Together, these panels indicate that the effective resistance partitioning (and therefore the resistance ratio) is driven primarily by variations in $R_{CsVs}$, while $R_{AsCs}$ remains comparatively constrained and does not provide a consistent monotonic signature with capillary pressure.

In addition to reproducing the targeted haemodynamic states, the generated populations were assessed for consistency with the qualitative correlation structure assumed during model formulation (Table 4). In Fig 11, the total systemic resistance shows no clear dependence on TBV and exhibits a strong negative correlation with cardiac output. The same figure shows that total compliance increases with blood volume, whereas total elastance decreases.

Fig 12 shows that the ratio $R_{AsCs}/(R_{AsCs}+R_{CsVs})$ increases with the fraction of pressure dropped across the arterial segment and decreases with the fraction dropped across the venous segment. The lower panels show the relationships between $R_{AsCs}$, $R_{CsVs}$ and ${\overset{―}{P}}_{Cs}$ in the calibrated populations.

Finally, Fig 13 shows that capillary compliance ($C_{Cs}$) decreases with increasing mean capillary pressure (${\overset{―}{P}}_{Cs}$), arterial compliance (*CAs*) decreases with pulse pressure ($PP_{As}$), and unstressed arterial volume (*V*_0,*As*_) increases with mean arterial volume (${\overset{―}{V}}_{As}$). The relationship between stroke volume and left-heart systolic elastance ($E_{Hl}$) is also shown.

**Fig 13:**
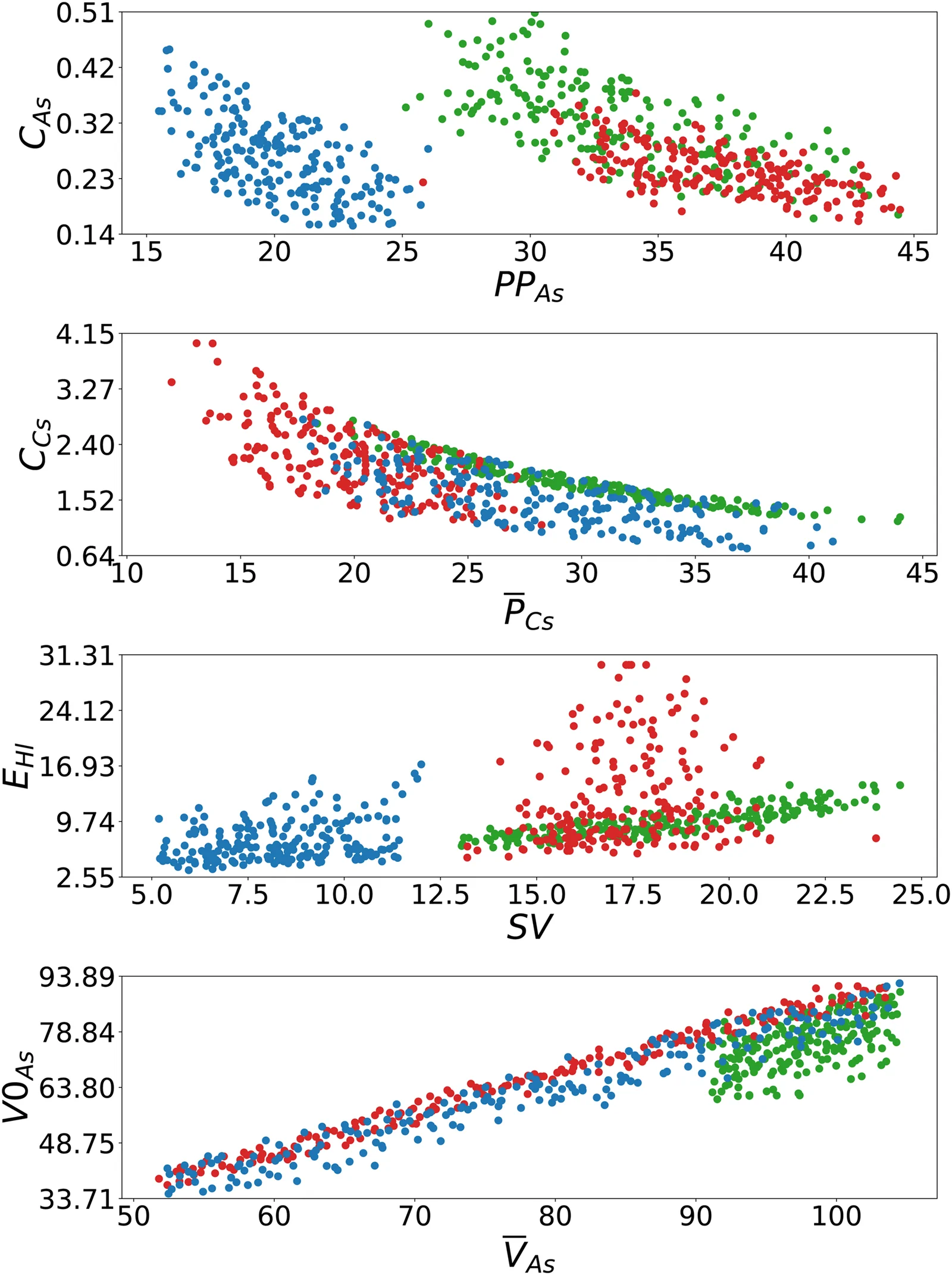
Scatter plots between arterial systemic compliance ($C_{As}$) and arterial pulse pressure ($PP_{As}$), systemic capillary compliance ($C_{Cs}$) and average capillary pressure (${\overset{―}{P}}_{Cs}$) systolic elastance of the left ventricle ($E_{Hl}$), and Stroke Volume (*SV*) and unstressed volume of the Systemic artery (*V*_0*As*_) and average volume of the systemic arteries (${\overset{―}{V}}_{As}$).

## 4. Discussion

**Convergence analysis** Despite all models having converged to solutions with very small residual errors and with coherent posterior parameter distributions when presented with the same calibration problem, they have done so in different ways. EFC calibration is performed on a fully dynamic, cycle-resolved system so targets such as systolic pressure, pulse pressure, stroke volume, and cycle-averaged volumes are computed using the max/min/average operators of Section 2.2.3 over the cardiac cycle, so each controller acts on a target error rebuilt once per cardiac cycle. Controlled variables and their associated parameters therefore never come to rest, and exhibit small, bounded oscillations around the target equilibrium. Unlike the cubic EFC, the Jacobian of the linear EFC does not change as error approaches 0, which makes the linear version of the technique approach the targets slower than the cubic version at high error situations and faster as the error approaches 0. This however makes the system overshoot the targets more as can be seen on the error spread of both methods at the end of calibration (see Fig 7). The cubic EFC is also more unstable as 27 of the 1024 samples failed to converge even when a staged gain approach was used to smoothen the initial response of the model to the error. The MCMC and the GD methods managed to find best solutions at lower errors but these are prone to get stuck at local minima as can be seen in the 35 samples of the GD with high error and the 778 rescues the MCMC algorithm performed.

All four methods can obtain solutions with errors below the resolution of the quantities a monitor reports. So the choice of method to use in a calibration procedure, like the one presented in this paper, will fall back to computational cost and reproducibility. Here EFC and its linear version in particular, requiring the least amount of model run-time (<50 s) and obtaining the least spread in the solution. For a given set of targets we always obtain the same solution within a very narrow range, from any admissible starting point. MCMC is still the go to option if a posterior distribution is needed in models that generate ambiguous solutions.

**Solution uniqueness** The convergence behaviour demonstrated in the previous section is a consequence of both the way the calibration problem was formulated, that sets up a unique solution scenario, and the inherent properties of the EFC that avoids local minima.

For an admissible set of targets, the calibration converges to a single physiological solution, and that solution is fixed by the target set imposed. Sixteen parameters are calibrated against sixteen targets, each parameter tied to a specific target through the correlations of Table 4, with each controller acting as a monotonic attractor that pulls its assigned variable toward the desired value. Those sixteen targets do not act as sixteen independent conditions on a sixteen-dimensional space. Eight of them are compartmental volumes imposed under a fixed total blood volume (Section 2.3.2), and one pressure waveform carries more than one condition: the systolic maximum and the pulse pressure of the systemic artery constrain the same variable at two points of the cardiac cycle, and both are produced by the same ventricular ejection. Every parameter influences multiple compartments, so each controller imposes a global constraint on the circulation. The resulting problem is strongly over-determined: most parameter combinations violate one or more of these conditions, and the only configuration satisfying all of them at once is the one at which every relative error $\sigma_{rel}$ vanishes. The assignment of Fig 6 determines which parameter carries which error, and with it the path the system takes to that configuration.

With all attractors active at once, any movement of one parameter immediately alters the conditions seen by the others, so the system cannot come to rest with a subset of the errors satisfied. The controller law admits no such equilibrium (Section 2.3.1), and the calibration forms no objective function whose slope could vanish away from the targets, so a partial solution is not a state the extended system can hold.

MCMC and GD both work on the objective of Eq. 21. On the majority of its restarts GD comes to rest away from the targets which leaves them unmet (Section 3.1), and a state that does not satisfy the targets is not a second solution. Independent support for the uniqueness of the calibrated state comes from MCMC, which carries no controllers, no parameter/target assignment and no monotonic correlations, and which concentrates on the region the 1024 controller calibrations occupy across all 16 parameters (Fig 8).

Fig 14 provides an illustrative low-dimensional example of the constraint structure induced by the EFC calibration. In this representation, pressure is plotted as a function of arterial compliance and volume according to Eq. 1. When compliance is used to regulate pressure in a given compartment, the corresponding controller defines a one-dimensional set of admissible parameter states for which the pressure target is satisfied ($\sigma_{rel}=0$, black curve), independent of the remainder of the system. Simultaneously, constraints imposed by the other controllers restrict the compartment to evolve along an independent trajectory (yellow curve). The calibrated solution corresponds to the unique state satisfying both constraints, indicated by their intersection (red marker).

**Fig 14:**
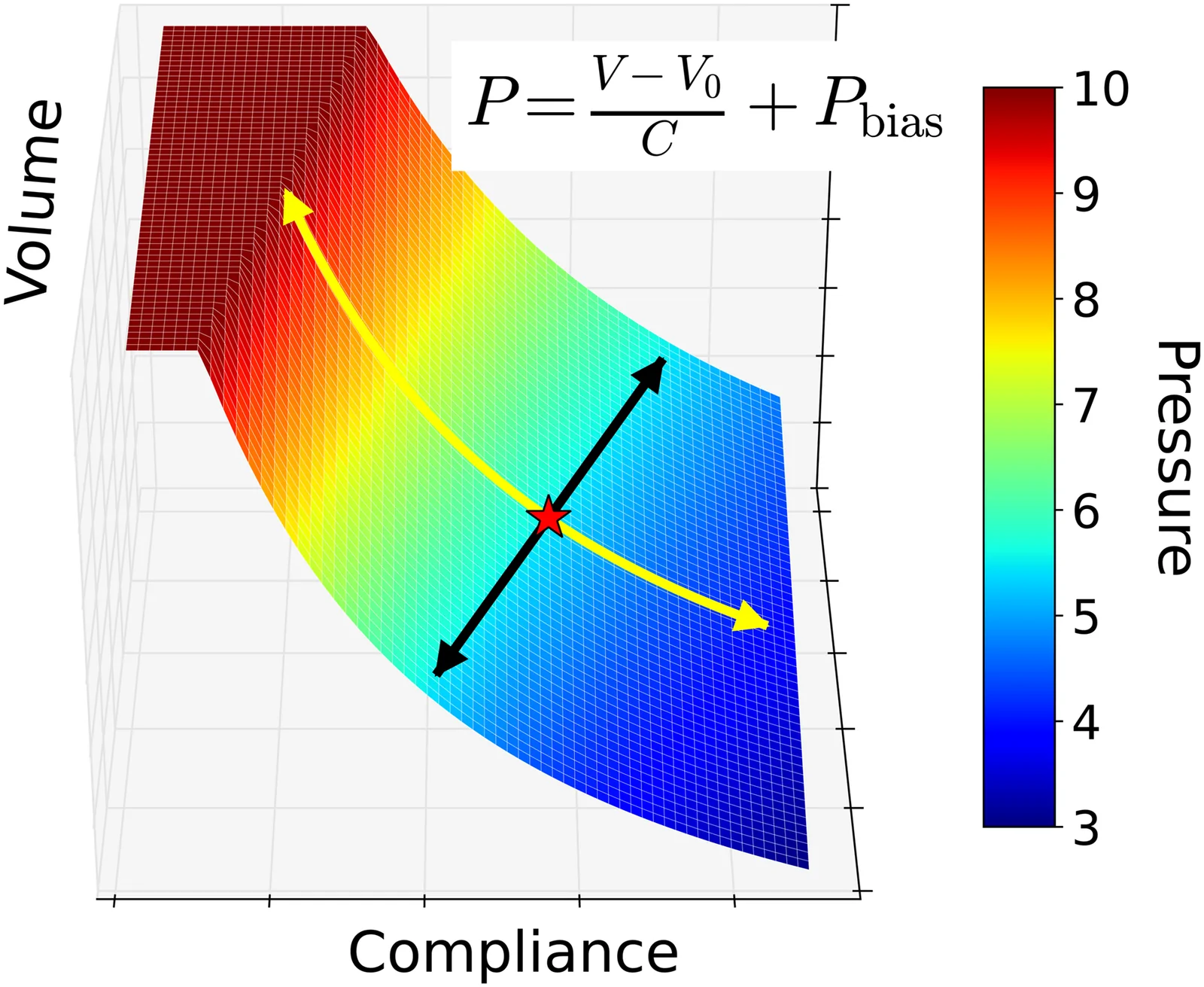
Conceptual illustration of solution uniqueness in the EFC calibration framework. Pressure is shown as a function of compartmental volume and compliance according to Eq. 1. One controller enforces the pressure target, defining a set of admissible parameter combinations (black curve), while constraints imposed by the remaining controllers restrict the system to an independent trajectory (yellow curve). The calibrated solution corresponds to the unique state satisfying both constraints simultaneously (red marker).

In the full cardiovascular model, the same principle applies in a higher-dimensional parameter space: each controller restricts the system to a subset of admissible states, and the simultaneous action of all controllers enforces convergence to their unique common intersection.

If a particular target set is incompatible with the model, one or more controllers drive their parameters to their bounds $y_{\min}$ or $y_{\max}$ while the corresponding $\sigma_{rel}$ remains non-zero. A saturated controller behaves as a constant parameter, effectively removing one attractor from the system. The remaining controllers still minimise their errors subject to this constraint, yielding a best-compromise equilibrium in which feasible targets are satisfied and infeasible ones are highlighted by persistent residual errors and parameters pinned at their limits.

**Sepsis Population** The error patterns observed in Fig 10 provide insight into how the calibration framework responds when prescribed targets become incompatible with the governing equation system. In the first pair of plots, saturation of the unstressed pulmonary arterial volume parameter *V*_0,*Ap*_ prevents further reduction once its lower bound is reached. At this point, the corresponding controller loses authority, rendering the target for ${\overset{―}{V}}_{Ap}$ unattainable in this regime. The calibration therefore proceeds with one fewer active constraint, while the remaining controllers continue to minimise their respective errors. The resulting persistent residual error is not indicative of algorithmic failure, but instead serves as a direct diagnostic of structural incompatibility between the imposed target combination and the model formulation.

A comparable mechanism is evident in the second pair of plots, where large negative residuals are associated with saturation of parameters governing cardiac contractility. In this case, the reduced control authority limits the model’s ability to increase stroke volume under the prescribed targets. Importantly, these saturation effects remain confined to specific regions of the parameter space, while the majority of the population exhibits small, unbiased errors. This indicates that the observed residuals reflect local feasibility limits rather than a loss of global calibration performance.

In contrast, the third pair of plots in Fig 10 illustrates a regime in which no controller saturation occurs and residual errors are smoothly distributed across the population. Here, convergence behaviour is dominated by the cubic control law, which produces progressively weaker corrective action as the error magnitude decreases. This leads to slow convergence near the target and the persistence of small residual errors of either sign. The direction of the final residual depends on the initial conditions, with trajectories approaching the target from either side.

Taken together, these observations show that large residual errors arise only when target specifications exceed the structural limits of the model, which are made explicit through parameter saturation. Outside these regimes, the calibration framework yields well-behaved error distributions while transparently exposing the boundaries of physiological feasibility imposed by the model structure.

**Population Parameters** The WS cohort exhibits low resistance across the explored volume range (Fig 11), consistent with near maximal systemic vasodilation, whereas the cold shock cohort spans higher and more variable resistance states, consistent with vasoconstrictive responses (Fig 1). Although warm shock also exhibits, on average, higher total compliance than cold shock, both septic cohorts show elevated compliance relative to normal physiology in the normal volume range, which is expected for WS but not for CS. The elastance panels suggest an additional cardiac contribution: at normal blood volumes, cold shock shows a marked reduction in total systolic elastance $E_{Total}$ relative to normal, while diastolic elastance $e_{Total}$ remains broadly comparable, a pattern consistent with impaired contractile strength with preserved diastolic properties and therefore compatible with a cardiogenic component. Conversely, in the low-volume regime the warm shock cohort exhibits substantially elevated $E_{Total}$, implying a configuration that would require disproportionately high systolic stiffness to sustain flow under combined hypovolaemia and vasodilation, and is therefore unlikely to represent a stable haemodynamic state over time.

From a physiological perspective, warm shock is characterised by a state of generalised vasodilation, which primarily affects the arterial compartment, while venous resistance is typically less responsive. Under such conditions, one would expect a lower arterial/capillary resistance ratio and a larger fraction of the total pressure drop to occur downstream, at the venous level. In contrast, cold shock is associated with arterial vasoconstriction, leading to higher resistance ratios and a larger pressure drop upstream. However, inspection of the parameter distributions of Fig 12 indicates that this behaviour arises primarily from variations in venous resistance, while arterial resistance in WS remains comparatively constrained across populations. As a result, changes in the resistance ratio are driven predominantly by the venous compartment rather than by arterial vasodilation. This parameter distribution reflects the way population targets were specified. Identical fractional pressure-drop ranges (30–70%) were imposed for both warm and cold shock cohorts, while systolic arterial pressure targets were also shared across populations. At the same time, higher pulse pressure targets in warm shock imply lower diastolic arterial pressures, which, combined with percentage-based capillary pressure targets relative to diastolic pressure and CVP, systematically bias the warm shock population towards lower capillary pressure targets. A more physiologically faithful separation between warm and cold shock could be achieved by assigning lower arterial pressure-drop ranges to the warm shock population and higher ranges to the cold shock population, thereby explicitly biasing the resistance ratio towards arterial vasodilation or vasoconstriction, respectively.

Beyond matching the target haemodynamic ranges, the calibrated populations broadly preserve the qualitative correlation structure assumed during model formulation (Table 4), which provides an internal consistency check on the generated parameter space. In Fig 11, the inverse coupling between $R_{Total}$ and cardiac output is maintained across cohorts, while the increase of $C_{Total}$ with TBV and the concomitant reduction in effective elastance with filling reflect the expected behaviour of compliant compartments under volume loading. Fig 12 further demonstrates that the resistance partitioning remains mechanically coherent: the ratio $R_{AsCs}/(R_{AsCs}+R_{CsVs})$ varies monotonically with the prescribed fractional pressure-drop allocations, consistent with resistors arranged in series. Also in agreement with the correlation assumptions, Fig 12 shows that the arterial resistance $R_{AsCs}$(inlet resistor) is positively correlated with the mean capillary pressure $\overset{―}{P}Cs$, while the venous resistance *RCsVs* (outlet resistor) is negatively correlated with the mean capillary pressure ${\overset{―}{P}}_{Cs}$. Finally, Fig 13 shows that the imposed local compliance/pressure and compliance/pulse-pressure relationships are retained ($C_{Cs}$ decreasing with ${\overset{―}{P}}_{Cs}$ and $C_{As}$ decreasing with $PP_{As}$), and that the scaling between *V*_0,*As*_ and ${\overset{―}{V}}_{As}$ remains approximately linear across the sampled space. Taken together, these patterns support that the calibrated populations are not arbitrary collections of parameters but occupy a physiologically structured manifold consistent with the model’s embedded assumptions.

**Autonomic regulation** The model deliberately omits an explicit autonomic or baroreflex loop. The purpose of EFC is to establish the operating point of the model, that is, the parameter set and the associated initial conditions from which any subsequent simulation departs, and this operating point is inferred from a steady-state clinical snapshot in which the autonomic contribution is a state that has already influenced physiology. The effect of the autonomic nervous system on HR is observed on the monitor and can be input into the model directly, whereas its effect on the peripheral resistances and cardiac contractility affects the observed pressures and flows that we are calibrating against. The cohort parameter distributions discussed above illustrate this: the low systemic resistances of the warm shock population and the reduced systolic elastance of the cold shock population are the haemodynamic signatures of the compensatory responses depicted in Fig 1. A baroreflex formulation would moreover act on the same quantities that the calibration controllers of Section 2.3 already actuate, so that the two feedback systems would compete for control authority over the set of parameters. Reflex equations become informative only once dynamic simulations are involved, for example when estimating the effect of a fluid bolus on the system. In this situation the proposed EFC technique could also approach the problem but in a different direction. By calibrating the model independently to every point of the observed trend, we would obtain a trend of model parameters that could be used to inform the creation of the ANS equations.

**Limitations** Despite the strengths of the proposed approach, several limitations should be acknowledged.

The study is entirely computational: the virtual cohorts were built from literature-derived target ranges and were never compared against patient recordings. Those ranges come from separate clinical studies, each reporting its own cohort, so an individual virtual subject may combine values that are each plausible in isolation yet rarely co-occur in a single child. Target combinations that are structurally incompatible with the model are rejected by the calibration itself as persistent residuals, but mechanical feasibility is not the same as clinical co-occurrence, and establishing the latter would require a real cohort in which the relevant quantities are measured concurrently.

In addition, calibration was performed for steady-state snapshots. Each controller consumes one cycle-based statistic per target, so the calibration is driven by the trend-level quantities a monitor records. The shape of the pressure or flow signal within the cardiac cycle cannot be used as a calibration target.

The population generation assumed a common reference distribution of total blood volume across compartments. While this choice supports direct comparability of inferred parameter sets across individuals and physiological states, the results indicate that it does not eliminate the need to explore controlled deviations from the normal distribution. In particular, persistent residual errors in arterial volume–related targets, such as ${\overset{―}{V}}_{Ap}$, highlight regimes in which the imposed volume partitioning is not fully compatible with the governing equation system. Under pathological conditions such as hypovolaemia and septic shock, physiological adaptation is expected to involve redistribution of blood volume between vascular compartments, making systematic variation of volume partitioning a necessary extension of the present framework.

The present model does not include inertial (inductive) effects of blood flow; while this simplification has no impact on steady-state calibration, other than reparameterisation, such effects become relevant under dynamic conditions, where frequency-dependent responses and resonance phenomena may influence transient haemodynamics and should be considered in future extensions.

The model is also restricted to haemodynamic variables. Sepsis is characterised by circulatory dysfunction together with metabolic derangements, such as elevated lactate, and impaired gas exchange, for which the capillary network plays a central physiological role. Incorporating blood gas fractions, lactate kinetics, and membrane transport and equilibrium dynamics would provide additional constraints on capillary behaviour and help inform the structural and functional characteristics required to represent microcirculatory function more faithfully.

Finally, every target used in this work is exact and internally consistent, drawn from a single admissible configuration of the model. Monitor targets carry measurement uncertainty, are recorded at different times and sampling intervals, and can be mutually incompatible for reasons unconnected to the model structure. The two baselines carry a residual scale $\sigma_{i}$ in Eq. 21 that sets how much mismatch their objective tolerates; the controller law of Eq. 16 carries no equivalent and drives every target error toward zero. Calibration against measured targets would need that residual read as a tolerance.

## 5. Conclusion

In this work, we developed and validated a mechanistic cardiovascular model coupled to an Embedded Feedback Controller (EFC) calibration framework capable of generating physiologically consistent solutions across multiple haemodynamic states, including normal physiology as well as warm and cold septic shock. The proposed calibration strategy consistently drives the model towards the prescribed physiological targets and yields calibrated solutions with low residual error across a broad range of physiological conditions. The set of targets imposed here over-determines the model and admits a single solution, reached by any calibration at any admissible starting point. The use of a common blood volume distribution across all populations was a deliberate design choice, ensuring that calibrated parameter sets remain directly comparable between individuals and across physiological states. This uniformity is essential for population-level analyses and for attributing observed differences to underlying haemodynamic adaptations.

A further important outcome of this work is that residual calibration errors provide interpretable information about the internal consistency of the model/target configuration. In particular, persistent residuals occur when specific target combinations are incompatible with the governing constraints, and are then directly associated with controller saturation, which exposes those feasibility limits in an explicit and interpretable manner. Interpreting residual error as a diagnostic of structural feasibility, rather than as optimisation failure, directly reflects the methodological principles advocated in recent guidelines for mechanistic cardiovascular modelling, which emphasise physiological interpretability, explicit handling of identifiability limits, and the integration of calibration within the governing model structure rather than as an external black-box procedure [28].

An additional advantage of the EFC-based calibration approach is its scalability. Unlike traditional optimisation-based calibration methods, whose computational cost typically grows rapidly with the number of calibrated parameters, the controller-based formulation introduces one additional constraint equation per parameter–target pair. As a result, expanding the calibrated parameter set does not fundamentally alter the stability of the optimisation landscape and incurs only marginal computational overhead.

In the present work, calibration was performed for individual steady-state snapshots. However, the proposed framework naturally extends to the calibration of longitudinal data, where successive time points are expected to yield closely related solutions. In this setting, the calibrated parameter set at a given time provides a natural initialisation for the subsequent calibration step, allowing the controllers to efficiently track gradual physiological changes and follow patient-specific trends over the course of an ICU stay.

## 6. A Appendix

### 6.1. A.1 Nomenclature

See Table 7.

**Table 7:** Nomenclature used in the cardiovascular model and calibration framework.

Symbol	Compartment
Hl	Left ventricle
Hr	Right ventricle
As	Systemic arteries
Ap	Pulmonary arteries
Cs	Systemic capillaries
Cp	Pulmonary capillaries
Vs	Systemic veins
Vp	Pulmonary veins
Vt	Thoracic veins
**Symbol**	**Primary Variable / Parameter**
$P_{*}$	Pressure in compartment *
$Q_{*†}$	Blood flow rate from compartment * to †
$V_{*}$	Blood volume in compartment *
$C_{*}$	Compliance of compartment *
$R_{*†}$	Resistance between compartments * and †
$V_{0*}$	Unstressed volume of compartment *
$E_{H*}$	Max (systolic) elastance of heart H*
$e_{H*}$	Min (diastolic) elastance of heart H*
${ℰ}_{H*}(\tau )$	Time-varying elastance of heart H*
$R^{in},R^{out}$	Generic inlet and outlet resistances
$P_{Bias}$	Pressure offset in capacitor equation
$P_{Atm}$	Atmospheric reference pressure
**Symbol**	**Clinical / Derived Quantity**
$SysP_{*}$	Systemic arterial systolic blood pressure
*PP*	Pulse pressure
*SV*	Stroke volume
*CO*	Cardiac output
*HR*	Heart rate
*TBV*	Total blood volume
*CVP*	Central venous pressure
**Symbol**	**Auxiliary / Operator Variable**
*Y*(*t*)	Generic scalar physiological/aux. variable
*min*(*Y*)	Min of *Y* over the current cardiac cycle
*max*(*Y*)	Max of *Y* over the current cardiac cycle
*avg*(*Y*)	Moving or cycle-average of *Y*
*int*(*Y*)	Integral of *Y* over the current cardiac cycle
*keep*(*Y*)	Value of *Y* stored from the previous cycle
${𝒪}_{1}({𝒪}_{2}(Y))$	Composition of operators acting on *Y*
**Symbol**	**Time Variable**
*t*	Absolute simulation time
τ	Intra-cardiac-cycle time (phase variable)
*HC*	Duration of a cardiac cycle
$T_{trig}$	Trigger time of the next cardiac cycle
$T_{avg}$	Averaging window duration
ε	Infinitesimal reset time of the cycle operators ($\varepsilon \to 0^{+}$)
Δt	Numerical integration time step
**Symbol**	**Calibration / Controller Variable**
$Y_{\min},Y_{\max}$	Lower and upper bounds of parameter *Y*
$Y_{target}$	Target value for variable *Y*
$Y_{current}$	Current model value of *Y*
$\sigma_{rel}$	Relative error $(Y_{target}-Y_{current})/Y_{target}$
*a*	Controller gain (scaling factor)

### 6.2. A.2 Model equations

**Capacitor Equations** The pressures for all capacitive elements listed in Table 3 follow the generic compliance relationship of Eq. 1. For each compartment *x*, the pressure is given by


$$\begin{array}{c} P_{x}=\frac{V_{x}-V_{0,x}}{C_{x}}+P_{\text{Bias},x}. \end{array}$$

Using the symbols from the table, the corresponding expressions are:


$$\begin{array}{c} P_{As}=\frac{V_{As}-V_{0,As}}{C_{As}}+P_{Atm},\;P_{Cs}=\frac{V_{Cs}-V_{0,Cs}}{C_{Cs}}+P_{Atm}, \end{array}$$

$$\begin{array}{c} P_{Vs}=\frac{V_{Vs}-V_{0,Vs}}{C_{Vs}}+P_{Atm},\;P_{Vt}=\frac{V_{Vt}-V_{0,Vt}}{C_{Vt}}+P_{Thx}, \end{array}$$

$$\begin{array}{c} P_{Ap}=\frac{V_{Ap}-V_{0,Ap}}{C_{Ap}}+P_{Thx},\;P_{Cp}=\frac{V_{Cp}-V_{0,Cp}}{C_{Cp}}+P_{La}, \end{array}$$

$$\begin{array}{c} P_{Vp}=\frac{V_{Vp}-V_{0,Vp}}{C_{Vp}}+P_{Thx}. \end{array}$$

The ventricles use time-varying elastance (Eq. 7):


$$\begin{array}{c} P_{Hr}(t)={ℰ}_{Hr}(t,(V_{Hr}-V_{0,Hr})+P_{Thx}, \end{array}$$

$$\begin{array}{c} P_{Hl}(t)={ℰ}_{Hl}(t,(V_{Hl}-V_{0,Hl})+P_{Thx}. \end{array}$$

**Elastance Definitions** The time-varying elastance functions for the left and right heart chambers follow the generic formulation of Eq. 7, with chamber-specific parameters.

For the left heart (Hl), the elastance is defined as


$$\begin{array}{cc} E_{\text{rise},Hl}(t) & =\frac{E_{Hl}-e_{Hl}}{2}\cdot \left[1-\cos\;\left(\frac{\pi \cdot t}{T_{\text{rise}}}\right)\right], \end{array}$$ (25)

$$\begin{array}{cc} E_{\text{fall},Hl}(t) & =\frac{E_{Hl}-e_{Hl}}{2}\cdot \left[1+\cos\;\left(\frac{\pi \cdot (t-T_{\text{rise}})}{T_{\text{fall}}}\right)\right], \end{array}$$ (26)

$$\begin{array}{cc} {ℰ}_{Hl}(t) & =\left\{ \begin{array}{cc} e_{Hl}+E_{\text{rise},Hl}(t), & 0<t<T_{\text{rise}},\\ e_{Hl}+E_{\text{fall},Hl}(t), & T_{\text{rise}}\leq t\leq T_{\text{rise}}+T_{\text{fall}},\\ e_{Hl}, & T_{\text{rise}}+T_{\text{fall}}<t, \end{array}\right. \end{array}$$ (27)

Similarly, for the right heart (Hr), the elastance is given by


$$\begin{array}{cc} E_{\text{rise},Hr}(t) & =\frac{E_{Hr}-e_{Hr}}{2}\cdot \left[1-\cos\;\left(\frac{\pi \cdot t}{T_{\text{rise}}}\right)\right], \end{array}$$ (28)

$$\begin{array}{cc} E_{\text{fall},Hr}(t) & =\frac{E_{Hr}-e_{Hr}}{2}\cdot \left[1+\cos\;\left(\frac{\pi \cdot (t-T_{\text{rise}})}{T_{\text{fall}}}\right)\right], \end{array}$$ (29)

$$\begin{array}{cc} {ℰ}_{Hr}(t) & =\left\{ \begin{array}{cc} e_{Hr}+E_{\text{rise},Hr}(t), & 0<t<T_{\text{rise}},\\ e_{Hr}+E_{\text{fall},Hr}(t), & T_{\text{rise}}\leq t\leq T_{\text{rise}}+T_{\text{fall}},\\ e_{Hr}, & T_{\text{rise}}+T_{\text{fall}}<t, \end{array}\right. \end{array}$$ (30)

**Resistor and Diode Flow Equations** The resistive elements in Table 3 follow either the linear resistor law (Eq. 2)


$$\begin{array}{c} Q_{x}=\frac{P_{\text{in},x}-P_{\text{out},x}}{R_{x}}, \end{array}$$

or the diode law (Eq. 4)


$$\begin{array}{c} Q_{x}=\left\{ \begin{array}{cc} \frac{P_{\text{in},x}-P_{\text{out},x}}{R_{x}}, & P_{\text{in},x}>P_{\text{out},x},\\ 0, & \text{otherwise}. \end{array}\right. \end{array}$$

The symbolic flow expressions are:


$$\begin{array}{c} Q_{Vt\_Hr}=\left\{ \begin{array}{cc} \frac{P_{Vt}-P_{Hr}}{R_{Vt\_Hr}}, & P_{Vt}>P_{Hr},\\ 0, \end{array}\right. \end{array}$$

$$\begin{array}{c} Q_{Hr\_Ap}=\left\{ \begin{array}{cc} \frac{P_{Hr}-P_{Ap}}{R_{Hr\_Ap}}, & P_{Hr}>P_{Ap},\\ 0, \end{array}\right. \end{array}$$

$$\begin{array}{c} Q_{Vp\_Hl}=\left\{ \begin{array}{cc} \frac{P_{Vp}-P_{Hl}}{R_{Vp\_Hl}}, & P_{Vp}>P_{Hl},\\ 0, \end{array}\right. \end{array}$$

$$\begin{array}{c} Q_{Hl\_As}=\left\{ \begin{array}{cc} \frac{P_{Hl}-P_{As}}{R_{Hl\_As}}, & P_{Hl}>P_{As},\\ 0. \end{array}\right. \end{array}$$

Linear resistors:


$$\begin{array}{c} Q_{Ap\_Cp}=\frac{P_{Ap}-P_{Cp}}{R_{Ap\_Cp}},\;Q_{Cp\_Vp}=\frac{P_{Cp}-P_{Vp}}{R_{Cp\_Vp}}, \end{array}$$

$$\begin{array}{c} Q_{As\_Cs}=\frac{P_{As}-P_{Cs}}{R_{As\_Cs}},\;Q_{Cs\_Vs}=\frac{P_{Cs}-P_{Vs}}{R_{Cs\_Vs}}, \end{array}$$

$$\begin{array}{c} Q_{Vs\_Vt}=\frac{P_{Vs}-P_{Vt}}{R_{Vs\_Vt}}. \end{array}$$

**Volume Balance Equations** All compartment volumes in Table 3 evolve according to the conservation law of Eq. 3:


$$\begin{array}{c} \frac{dV_{x}}{dt}=\sum Q_{x}^{\text{in}}-\sum Q_{x}^{\text{out}}. \end{array}$$

The symbolic volume dynamics are therefore:


$$\begin{array}{c} \frac{dV_{As}}{dt}=Q_{Hl\_As}-Q_{As\_Cs},\;\frac{dV_{Cs}}{dt}=Q_{As\_Cs}-Q_{Cs\_Vs}, \end{array}$$

$$\begin{array}{c} \frac{dV_{Vs}}{dt}=Q_{Cs\_Vs}-Q_{Vs\_Vt},\;\frac{dV_{Vt}}{dt}=Q_{Vs\_Vt}-Q_{Vt\_Hr}, \end{array}$$

$$\begin{array}{c} \frac{dV_{Hr}}{dt}=Q_{Vt\_Hr}-Q_{Hr\_Ap},\;\frac{dV_{Ap}}{dt}=Q_{Hr\_Ap}-Q_{Ap\_Cp}, \end{array}$$

$$\begin{array}{c} \frac{dV_{Cp}}{dt}=Q_{Ap\_Cp}-Q_{Cp\_Vp},\;\frac{dV_{Vp}}{dt}=Q_{Cp\_Vp}-Q_{Vp\_Hl}, \end{array}$$

$$\begin{array}{c} \frac{dV_{Hl}}{dt}=Q_{Vp\_Hl}-Q_{Hl\_As}. \end{array}$$

**Calibration variables based on cycle averages and integrals** The calibration targets in Table 2 are computed by instantiating the generic max/min, integral, keeper, and moving-average equations (12)–(15) with specific model variables. Below we write the explicit forms for the quantities used in the calibration procedure.

**Cycle-averaged compartment volumes** The cycle-averaged volumes are obtained using the moving-average equation 15, with $Y(T)=V_{x}(T)$ and $Avg_{Y}(T)=avg\_V\_x(T)$:


$$\begin{array}{cc} \frac{d\;{\overset{―}{V}}_{Hl}}{dT} & =\frac{V_{Hl}(T)-{\overset{―}{V}}_{Hl}(T)}{\tau_{V}}, \end{array}$$ (31)

$$\begin{array}{cc} \frac{d\;{\overset{―}{V}}_{Hr}}{dT} & =\frac{V_{Hr}(T)-{\overset{―}{V}}_{Hr}(T)}{\tau_{V}}, \end{array}$$ (32)

$$\begin{array}{cc} \frac{d\;{\overset{―}{V}}_{As}}{dT} & =\frac{V_{As}(T)-{\overset{―}{V}}_{As}(T)}{\tau_{V}}, \end{array}$$ (33)

$$\begin{array}{cc} \frac{d\;{\overset{―}{V}}_{Cs}}{dT} & =\frac{V_{Cs}(T)-{\overset{―}{V}}_{Cs}(T)}{\tau_{V}}, \end{array}$$ (34)

$$\begin{array}{cc} \frac{d\;{\overset{―}{V}}_{Vt}}{dT} & =\frac{V_{Vt}(T)-{\overset{―}{V}}_{Vt}(T)}{\tau_{V}}, \end{array}$$ (35)

$$\begin{array}{cc} \frac{d\;{\overset{―}{V}}_{Ap}}{dT} & =\frac{V_{Ap}(T)-{\overset{―}{V}}_{Ap}(T)}{\tau_{V}}, \end{array}$$ (36)

$$\begin{array}{cc} \frac{d\;{\overset{―}{V}}_{Cp}}{dT} & =\frac{V_{Cp}(T)-{\overset{―}{V}}_{Cp}(T)}{\tau_{V}}, \end{array}$$ (37)

$$\begin{array}{cc} \frac{d\;{\overset{―}{V}}_{Vp}}{dT} & =\frac{V_{Vp}(T)-{\overset{―}{V}}_{Vp}(T)}{\tau_{V}}. \end{array}$$ (38)

where $\tau_{V}$ is the averaging window (e.g., several seconds or beats). The systemic venous volume $V_{Vs}$ is used directly as a state variable and not averaged.

**Stroke volume of the left ventricle** Stroke volume is computed from the flow $Q_{Hl\_As}$ using the integral-reset equation 13, with $Y(T)=Q_{Hl\_As}(T)$:


$$\begin{array}{c} \frac{d\;SV_{int,Hl}}{dT}=\left\{ \begin{array}{cc} -\frac{Q_{Hl\_As}(T)}{\varepsilon }, & T-T_{trigger}(T)\geq 0,\\ Q_{Hl\_As}(T), & T-T_{trigger}(T)<0, \end{array}\right. \end{array}$$ (39)

where $SV_{int,Hl}$ accumulates the outflow over a single cardiac cycle. The cycle-wise stroke volume used for calibration, keep_SV_Hl, is obtained via the keeper equation 14 with $Y(T)=SV_{int,Hl}(T)$:


$$\begin{array}{c} SV_{\text{update},Hl}(T)=\frac{SV_{\text{int},Hl}(T)-keep\_SV\_Hl(T)}{\varepsilon } \end{array}$$ (40)

$$\begin{array}{c} \frac{d\;keep\_SV\_Hl}{dT}=\left\{ \begin{array}{cc} SV_{\text{update},Hl}(T), & T-T_{\text{trigger}}(T)\geq 0,\\ 0, & T-T_{\text{trigger}}(T)<0. \end{array}\right. \end{array}$$ (41)

**Systolic, diastolic, and pulse pressures in the systemic arteries** For the systemic arterial pressure $P_{As}$, we define within-cycle maxima and minima using the max/min equations (12–11) with $Y(T)=P_{As}(T)$:


$$\begin{array}{c} \Delta_{As}^{\max}(T)=\frac{P_{As}(T)-max\_P\_As(T)}{\varepsilon } \end{array}$$ (42)

$$\begin{array}{c} \Delta_{As}^{\min}(T)=\frac{P_{As}(T)-min\_P\_As(T)}{\varepsilon } \end{array}$$ (43)

$$\begin{array}{cc} \frac{d\;max\_P\_As}{dT} & =\left\{ \begin{array}{cc} \Delta_{As}^{\max}(T), & \begin{array}{c} T\geq T_{trigger}(Tnonumber\\ \vee P_{As}(T)>max\_P\_As(T) \end{array}\\ {}[0.2em]0, & P_{As}(T)\leq max\_P\_As(T), \end{array}\right. \end{array}$$ (44)

$$\begin{array}{cc} \frac{d\;min\_P\_As}{dT} & =\left\{ \begin{array}{cc} \Delta_{As}^{\min}(T), & \begin{array}{c} T\geq T_{trigger}(Tnonumber\\ \vee P_{As}(T)<min\_P\_As(T) \end{array}\\ {}[0.2em]0, & P_{As}(T)\geq min\_P\_As(T). \end{array}\right. \end{array}$$ (45)

The cycle-wise systolic and diastolic pressures used in calibration are kept using Eq. 14:


$$\begin{array}{c} \Delta_{As}^{keep,max}(T)=\frac{max\_P\_As(T)-keep\_max\_P\_As(T)}{\varepsilon } \end{array}$$ (46)

$$\begin{array}{c} \Delta_{As}^{keep,min}(T)=\frac{min\_P\_As(T)-keep\_min\_P\_As(T)}{\varepsilon } \end{array}$$ (47)

$$\begin{array}{cc} \frac{d\;keep\_max\_P\_As}{dT} & =\left\{ \begin{array}{cc} \Delta_{As}^{keep,max}(T), & T-T_{trigger}(T)\geq 0,\\ 0, & T-T_{trigger}(T)<0, \end{array}\right. \end{array}$$ (48)

$$\begin{array}{cc} \frac{d\;keep\_min\_P\_As}{dT} & =\left\{ \begin{array}{cc} \Delta_{As}^{keep,min}(T), & T-T_{trigger}(T)\geq 0,\\ 0, & T-T_{trigger}(T)<0. \end{array}\right. \end{array}$$ (49)

The systemic arterial pulse pressure used in calibration is the difference between the two kept pressures,


$$\begin{array}{c} amp\_P\_As(T)=keep\_max\_P\_As(T)-keep\_min\_P\_As(T), \end{array}$$ (50)

and the pulmonary arterial pulse pressure amp_P_Ap is obtained in the same way from keep_max_P_Ap and keep_min_P_Ap.

**Calibration controller equations** The calibration targets in Table 2 are enforced by embedding the model parameters as additional state variables and driving them with the controller law of Eq. 16. For each controller *j*, the relative error $\sigma_{j}$ between the model quantity $X_{j}(T)$ and its target value $X_{j}^{⋆}$ is


$$\begin{array}{c} \sigma_{j}=\frac{X_{j}^{⋆}-X_{j}(T)}{\left|X_{j}(T)\right|}, \end{array}$$

and the associated parameter $\theta_{j}(T)$ is driven by


$$\begin{array}{c} \frac{d\theta_{j}}{dT}=\pm \;\Phi (\sigma_{j},\theta_{j}^{\max},\;\Phi (\sigma )=a_{C}\;\sigma^{3}+a_{L}\;\sigma , \end{array}$$ (51)

where $\theta_{j}^{\max}$ is the upper bound of the parameter and the sign in front of Φ is the correlation in Table 4 (‘+’ for positive, ‘-’ for negative). A target above the current value of the model quantity therefore moves $\theta_{j}$ in the direction that raises it.

Below we list the explicit error definitions $\sigma_{j}$ and parameter dynamics $\theta_{j}(T)$ for each controller used in Table 2. Superscripts ${\;}^{⋆}$ denote the corresponding target values (e.g., midpoints of the reported physiological ranges) for the selected phenotype (warm shock, cold shock, or normal).

**TBV controllers** Errors:


$$\begin{array}{cc} \sigma_{{\overset{―}{V}}_{Hl}} & =\frac{{\overset{―}{V}}_{Hl}^{⋆}-{\overset{―}{V}}_{Hl}(T)}{\left|{\overset{―}{V}}_{Hl}(T)\right|}, \end{array}$$ (52)

$$\begin{array}{cc} \sigma_{{\overset{―}{V}}_{Hr}} & =\frac{{\overset{―}{V}}_{Hr}^{⋆}-{\overset{―}{V}}_{Hr}(T)}{\left|{\overset{―}{V}}_{Hr}(T)\right|}, \end{array}$$ (53)

$$\begin{array}{cc} \sigma_{{\overset{―}{V}}_{As}} & =\frac{{\overset{―}{V}}_{As}^{⋆}-{\overset{―}{V}}_{As}(T)}{\left|{\overset{―}{V}}_{As}(T)\right|}, \end{array}$$ (54)

$$\begin{array}{cc} \sigma_{{\overset{―}{V}}_{Cs}} & =\frac{{\overset{―}{V}}_{Cs}^{⋆}-{\overset{―}{V}}_{Cs}(T)}{\left|{\overset{―}{V}}_{Cs}(T)\right|}, \end{array}$$ (55)

$$\begin{array}{cc} \sigma_{{\overset{―}{V}}_{Vt}} & =\frac{{\overset{―}{V}}_{Vt}^{⋆}-{\overset{―}{V}}_{Vt}(T)}{\left|{\overset{―}{V}}_{Vt}(T)\right|}, \end{array}$$ (56)

$$\begin{array}{cc} \sigma_{{\overset{―}{V}}_{Ap}} & =\frac{{\overset{―}{V}}_{Ap}^{⋆}-{\overset{―}{V}}_{Ap}(T)}{\left|{\overset{―}{V}}_{Ap}(T)\right|}, \end{array}$$ (57)

$$\begin{array}{cc} \sigma_{{\overset{―}{V}}_{Cp}} & =\frac{{\overset{―}{V}}_{Cp}^{⋆}-{\overset{―}{V}}_{Cp}(T)}{\left|{\overset{―}{V}}_{Cp}(T)\right|}, \end{array}$$ (58)

$$\begin{array}{cc} \sigma_{{\overset{―}{V}}_{Vp}} & =\frac{{\overset{―}{V}}_{Vp}^{⋆}-{\overset{―}{V}}_{Vp}(T)}{\left|{\overset{―}{V}}_{Vp}(T)\right|}. \end{array}$$ (59)

Parameter dynamics (correlation sign taken from Table 4):


$$\begin{array}{cc} \frac{d\;e_{Hl}}{dT} & =-\;\Phi (\sigma_{{\overset{―}{V}}_{Hl}},e_{Hl}^{\max}, \end{array}$$ (60)

$$\begin{array}{cc} \frac{d\;e_{Hr}}{dT} & =-\;\Phi (\sigma_{{\overset{―}{V}}_{Hr}},e_{Hr}^{\max}, \end{array}$$ (61)

$$\begin{array}{cc} \frac{d\;V_{0,As}}{dT} & =\;\;\Phi (\sigma_{{\overset{―}{V}}_{As}},V_{0,As}^{\max}, \end{array}$$ (62)

$$\begin{array}{cc} \frac{d\;C_{Cs}}{dT} & =\;\;\Phi (\sigma_{{\overset{―}{V}}_{Cs}},C_{Cs}^{\max}, \end{array}$$ (63)

$$\begin{array}{cc} \frac{d\;C_{Vt}}{dT} & =\;\;\Phi (\sigma_{{\overset{―}{V}}_{Vt}},C_{Vt}^{\max}, \end{array}$$ (64)

$$\begin{array}{cc} \frac{d\;V_{0,Ap}}{dT} & =\;\;\Phi (\sigma_{{\overset{―}{V}}_{Ap}},V_{0,Ap}^{\max}, \end{array}$$ (65)

$$\begin{array}{cc} \frac{d\;C_{Cp}}{dT} & =\;\;\Phi (\sigma_{{\overset{―}{V}}_{Cp}},C_{Cp}^{\max}, \end{array}$$ (66)

$$\begin{array}{cc} \frac{d\;C_{Vp}}{dT} & =\;\;\Phi (\sigma_{{\overset{―}{V}}_{Vp}},C_{Vp}^{\max}. \end{array}$$ (67)

**Cardiac output and heart rate controller** Stroke volume of the left ventricle is obtained by cycle integration of Q_Hl_As(T) and stored as keep_SV_Hl. The corresponding error is


$$\begin{array}{c} \sigma_{SVHl}=\frac{keep\_SV\_{Hl}^{⋆}-keep\_SV\_Hl(T)}{\left|keep\_SV\_Hl(T)\right|}, \end{array}$$ (68)

and the controller on the maximal left-ventricular elastance $E_{Hl}$ is


$$\begin{array}{c} \frac{dE_{Hl}}{dT}=\;\;\Phi (\sigma_{SVHl},E_{Hl}^{\max}. \end{array}$$ (69)

**Systemic arterial pressure and pulse pressure controllers** Let keep_max_P_As and keep_min_P_As denote the cycle-wise systolic and diastolic systemic arterial pressures. Their targets are $keep\_max\_P\_{As}^{⋆}$ and $amp\_P\_{As}^{⋆}$(pulse pressure), with


$$\begin{array}{c} {PP}_{As}(T)={keep}_{\max P_{As}}(T)-{keep}_{\min P_{As}}(T). \end{array}$$ (70)

We define


$$\begin{array}{cc} \sigma_{sys,As} & =\frac{{keep}_{\max P_{As}}^{⋆}-{keep}_{\max P_{As}}(T)}{\left|{keep}_{\max P_{As}}(T)\right|}, \end{array}$$ (71)

$$\begin{array}{cc} \sigma_{PP_{As}} & =\frac{{PP}_{As}^{⋆}-{PP}_{As}(T)}{\left|{PP}_{As}(T)\right|}, \end{array}$$ (72)

and drive the parameters


$$\begin{array}{cc} \frac{d\;R_{AsCs}}{dT} & =\;\;\Phi (\sigma_{sys,As},R_{AsCs}^{\max}, \end{array}$$ (73)

$$\begin{array}{cc} \frac{d\;C_{As}}{dT} & =-\;\Phi (\sigma_{PP_{As}},C_{As}^{\max}. \end{array}$$ (74)

**Central venous pressure controller** For the systemic venous pressure $P_{Vs}$, the cycle-averaged value ${\overset{―}{P}}_{Vs}(T)$ is compared with its target ${\overset{―}{P}}_{Vs}^{⋆}$:


$$\begin{array}{c} \sigma_{CVP}=\frac{{\overset{―}{P}}_{Vs}^{⋆}-{\overset{―}{P}}_{Vs}(T)}{\left|{\overset{―}{P}}_{Vs}(T)\right|}. \end{array}$$ (75)

and the venous compliance controller is


$$\begin{array}{c} \frac{dC_{Vs}}{dT}=-\;\Phi (\sigma_{CVP},C_{Vs}^{\max}. \end{array}$$ (76)

**Systemic capillary pressure controller** For the systemic capillary pressure $P_{Cs}$, the cycle-averaged value ${\overset{―}{P}}_{Cs}(T)$ is used:


$$\begin{array}{c} \sigma_{P_{Cs}}=\frac{{\overset{―}{P}}_{Cs}^{⋆}-{\overset{―}{P}}_{Cs}(T)}{\left|{\overset{―}{P}}_{Cs}(T)\right|}, \end{array}$$ (77)

and the venous resistance is adjusted as


$$\begin{array}{c} \frac{d\;R_{CsVs}}{dT}=\;\;\Phi (\sigma_{P_{Cs}},R_{CsVs}^{\max}. \end{array}$$ (78)

**Pulmonary arterial pressure controllers** For the pulmonary arterial pressure $P_{Ap}$, we define cycle-wise systolic and diastolic values $keep\_max\_P_{Ap}(T)$ and $keep\_min\_P_{Ap}(T)$ with targets $keep\_max\_P_{Ap}^{⋆}$ and $keep\_min\_P_{Ap}^{⋆}$:


$$\begin{array}{cc} \sigma_{sysAp} & =\frac{keep\_max\_P_{Ap}^{⋆}-keep\_max\_P_{Ap}(T)}{\left|keep\_max\_P_{Ap}(T)\right|}, \end{array}$$ (79)

$$\begin{array}{cc} \sigma_{diasAp} & =\frac{keep\_min\_P_{Ap}^{⋆}-keep\_min\_P_{Ap}(T)}{\left|keep\_min\_P_{Ap}(T)\right|}. \end{array}$$ (80)

The corresponding controllers are


$$\begin{array}{cc} \frac{dE_{Hr}}{dT} & =\;\;\Phi (\sigma_{sysAp},E_{Hr}^{\max}, \end{array}$$ (81)

$$\begin{array}{cc} \frac{dC_{Ap}}{dT} & =-\;\Phi (\sigma_{diasAp},C_{Ap}^{\max}. \end{array}$$ (82)

**Pulmonary capillary pressure controller** Finally, for the pulmonary capillary pressure $P_{Cp}$, the cycle-averaged value ${\overset{―}{P}}_{Cp}(T)$ is compared with its target ${\overset{―}{P}}_{Cp}^{⋆}$:


$$\begin{array}{c} \sigma_{P_{Cp}}=\frac{{\overset{―}{P}}_{Cp}^{⋆}-{\overset{―}{P}}_{Cp}(T)}{\left|{\overset{―}{P}}_{Cp}(T)\right|}, \end{array}$$ (83)

and the corresponding resistance controller is


$$\begin{array}{c} \frac{d\;R_{ApCp}}{dT}=-\;\Phi (\sigma_{P_{Cp}},R_{ApCp}^{\max}. \end{array}$$ (84)

These equations instantiate the controller law (16) for all calibration variables used in Table 2, linking each clinical target to a specific model parameter through the correlation structure indicated in the table.

### 6.3. A.3 Timing and integrator comparison

To confirm that the explicit Euler scheme does not compromise the accuracy of the calibrated solution, the same staged calibration was repeated under three solvers, the explicit Euler, a fixed-step fourth-order Runge-Kutta method (RK4), and the adaptive Dopri5 (an RK4(5) pair with adaptive time stepping). Starting from identical initial conditions and driving the same physiological twin targets. Table 8 reports, for each integrator, the residual accuracy at convergence (the maximum and mean absolute relative error across the calibration targets), the agreement of the calibrated parameters across integrators, and the wall-clock cost of the calibration. Fig 15 shows the per-target relative error and the per-integrator wall-clock time.

**Table 8:** Integrator comparison for one staged calibration driven to the same twin targets. $\left|\varepsilon_{r}\right|$ is the relative error against the targets, Δ p the run-end deviation of each calibrated parameter from the Euler reference. One calibration integrates 3700 s of simulated time (370 runs of 10 s), so *s / 10 s solve* is the cost of a single forward solve. All three agree to within 2.7%, but Dopri5 is ~87 × slower.

	euler	rk4	dopri5
Max $\left|\varepsilon_{r}\right|$%	2.73	2.74	2.74
Mean $\left|\varepsilon_{r}\right|$%	0.784	0.788	0.79
Max Δ p vs Euler %	0	1.24	1.28
s / 10 s solve	0.134	0.582	11.6
s / calibration	49.5	215.5	4284.2
Slowdown ×	1.0	4.4	86.5

**Fig 15:**
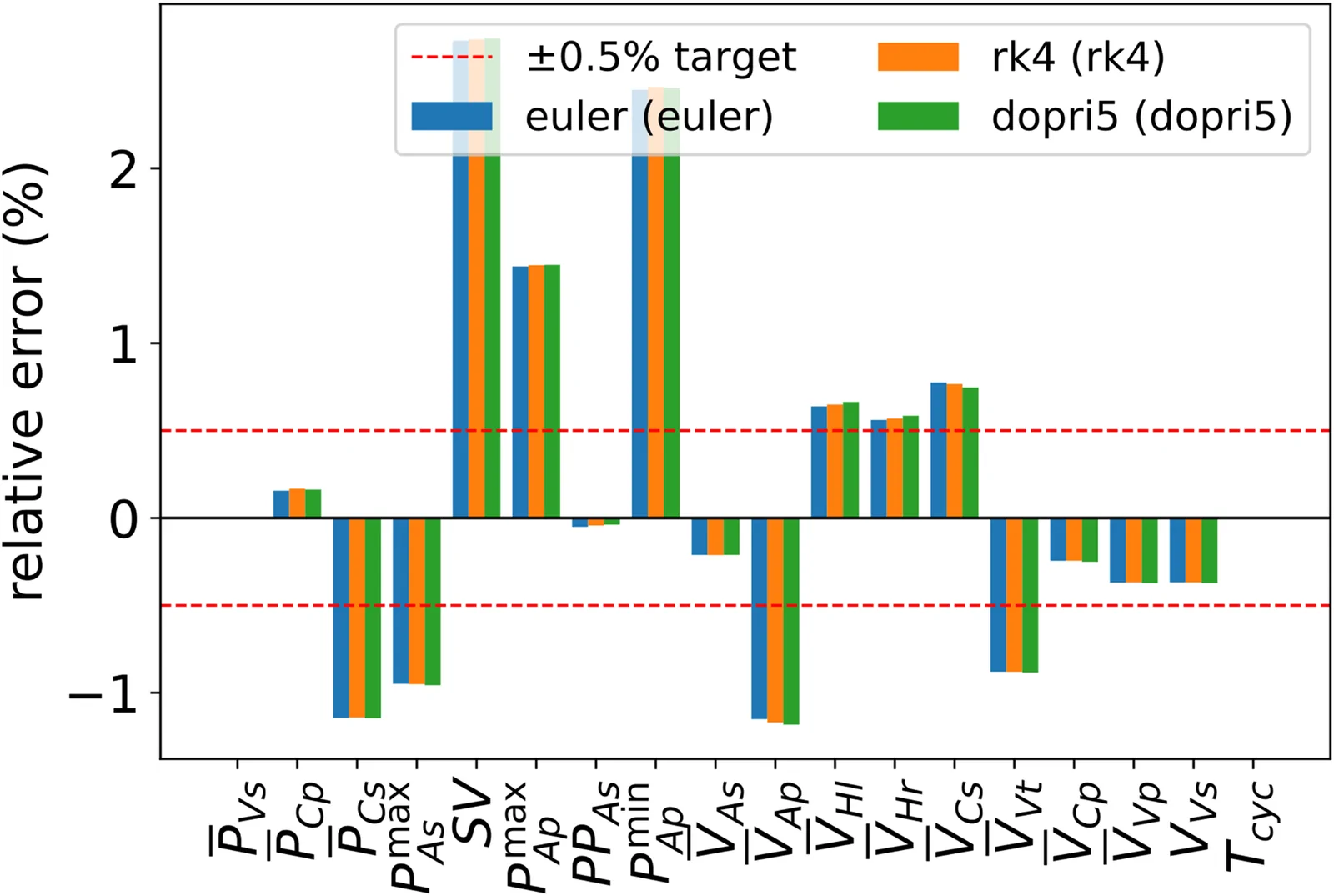
Signed relative error of each calibration target at convergence under explicit Euler, fixed-step RK4, and adaptive Dopri5. The three integrators overlie one another, reaching the same calibrated solution; their wall-clock costs are given in Table 8.

The three integrators converge to the same calibrated solution: the residual errors and the calibrated parameter values agree to within a small tolerance, confirming that the explicit Euler discretisation at Δt=0.0005s is still accurate for this system. On a single workstation CPU (Intel Core Ultra 7 155H, double precision), one 10s forward solve takes 0.134s under Euler, with a full calibration taking 49.5s, against 4.4× that under RK4 and 86.5× under Dopri5 (Fig 15).

**Fig 16:**
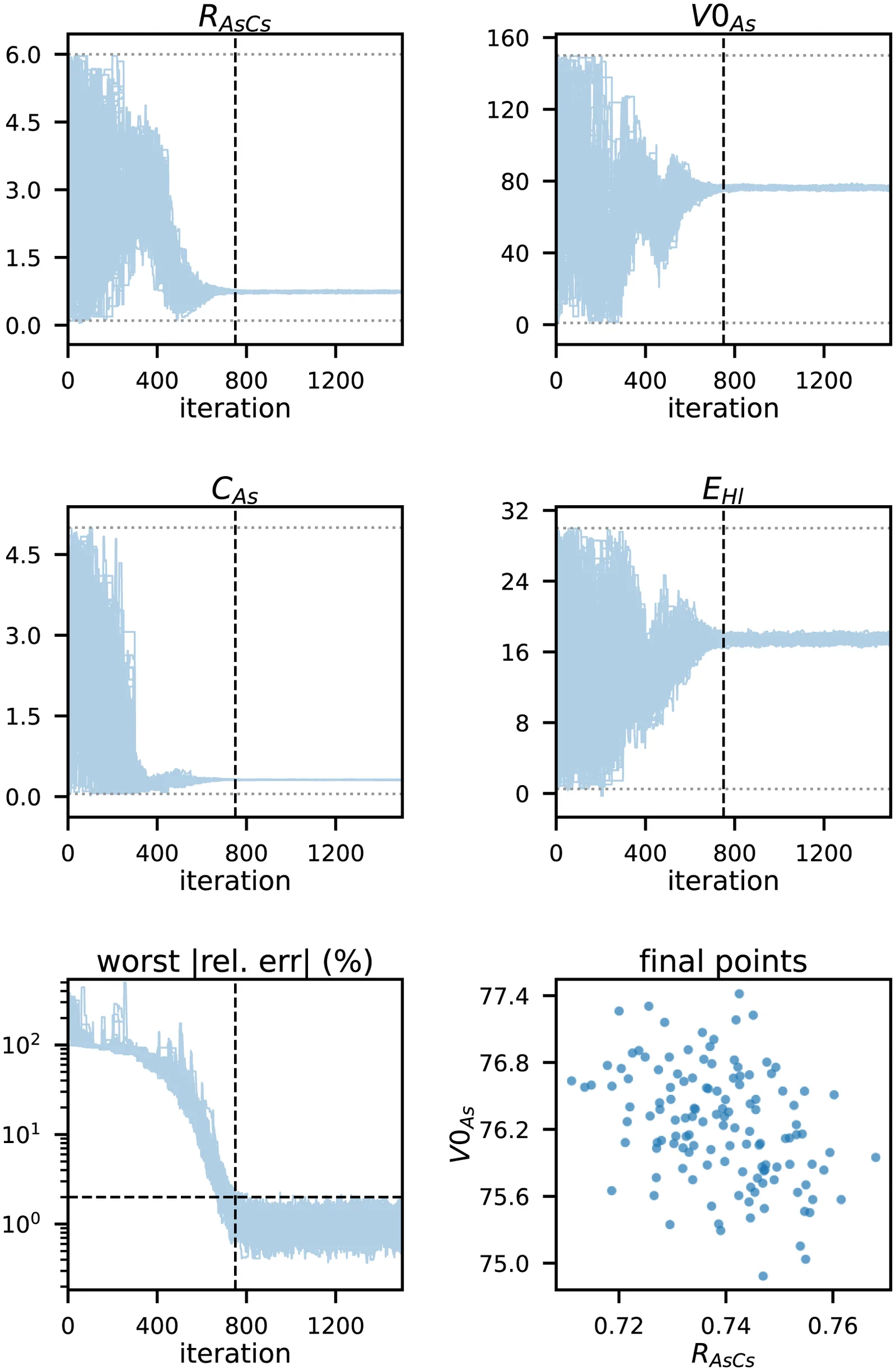
Walker histories of the ensemble MCMC baseline. Panels 1-4 show every walker’s trajectory for the four parameters on which the gradient-descent baseline of Fig 17 splits into two basins, with the prior bounds dotted and the end of burn-in marked, panel 5 each walker’s worst relative error over the 16 targets, panel 6 the walkers’ final positions. The ensemble collapses onto one connected region, and no walker visits the spurious basin at $R_{AsCs}\approx 0.375$.

The extra cost of Dopri5 is caused by the valves. Each perfect unidirectional valve makes the right-hand side discontinuous, and the step-size controller must shrink its step at every valve event to keep its error estimate inside tolerance. The adaptive machinery spends its effort resolving these switching events and gains no accuracy in return.

### 6.4. Ensemble MCMC baseline

The sampler is the affine-invariant ensemble sampler of Goodman and Weare [51], in the emcee implementation [52], run with 128 walkers for 1500 iterations of which the first 750 are discarded as burn-in, leaving 96 000 posterior samples. Walkers move in the physical parameters through a whitened proposal, each proposal is scored on three settling runs of the model, and the residual scale of Eq. 21 is $\sigma_{i}=0.005\;|t_{i}|$. The run was gated to stop on a split- $\hat{R}$ below 1.05 with at least 400 effective samples, whichever came later. Proposals are a mixture of differential-evolution and snooker moves, which mix better than the stretch move in 16 dimensions. Two measures were needed to make the run usable and both are worth recording, since neither is specific to this model. First, the sampler works in coordinates whitened by the covariance of a 512-point Latin-hypercube warm-up, restricted to its best-scoring fifth: the raw prior box is elongated by more than two orders of magnitude along some directions, and an unwhitened ensemble spends the entire run migrating in from the corners. Second, a walker whose log-posterior falls far below the ensemble median during burn-in is reseeded from a healthy walker, because the differential-evolution proposal scale is set by the spread of the *other* walkers and a walker stranded far from them can never random-walk back. The rescue is confined to burn-in; the sampling phase is left as detailed-balance Markov chain Monte Carlo.

The run converged on its own convergence criterion, with a maximum split- $\hat{R}$ of 1.048 across the 16 parameters, at least 500 effective samples for the least well-mixed of them, and a mean acceptance fraction of 0.267. It cost 192 000 forward solves, or 9077 s of wall-clock time on a single workstation CPU, for the ensemble as a whole. Table 9 reports the result in the same form as Table 6. The posterior mean reproduces every target to within 0.02 %, and every walker individually lands within 1.7 % of every target. 15 of the 16 parameters are identified to better than 2 %; the exception is the unstressed pulmonary arterial volume $V0_{Ap}$, whose posterior is 9 % wide and which is the single sloppy direction of the problem.

**Table 9:** Ensemble-MCMC baseline calibration, in the form of Table 6: for each of the sixteen targets, the prescribed value and the signed relative error over the full post-burn posterior (750 steps ×128 walkers = 96 000 samples), with the paired parameter’s posterior mean and standard deviation. Walker histories are shown in Fig 16.

Vars.	Targets	Relative Error %	std	Param.	Value	std
${\overset{―}{P}}_{Vs}$	8	-0.017	0.474	$C_{Vs}$	59.851	0.292
${\overset{―}{P}}_{Cp}$	11.5	-0.015	0.484	$R_{ApCp}$	0.164	0.002
${\overset{―}{P}}_{Cs}$	37.5	-0.012	0.481	$R_{CsVs}$	0.710	0.006
$P_{As}^{\max}$	85	-0.007	0.481	$R_{AsCs}$	0.739	0.012
*SV*	19.23	0.000	0.476	$E_{Hl}$	17.417	0.274
$P_{Ap}^{\max}$	22.5	0.009	0.484	$E_{Hr}$	4.020	0.058
$PP_{As}$	35	-0.002	0.488	$C_{As}$	0.312	0.003
$P_{Ap}^{\min}$	13.5	-0.024	0.481	$C_{Ap}$	1.181	0.022
${\overset{―}{V}}_{As}$	97.5	-0.006	0.474	$V0_{As}$	76.278	0.558
${\overset{―}{V}}_{Ap}$	26.25	0.012	0.476	$V0_{Ap}$	4.659	0.417
${\overset{―}{V}}_{Hl}$	15	0.010	0.482	$e_{Hl}$	0.444	0.003
${\overset{―}{V}}_{Hr}$	15	-0.022	0.483	$e_{Hr}$	0.258	0.002
${\overset{―}{V}}_{Cs}$	37.5	-0.020	0.478	$C_{Cs}$	1.020	0.007
${\overset{―}{V}}_{Vt}$	30	0.001	0.484	$C_{Vt}$	4.452	0.033
${\overset{―}{V}}_{Cp}$	22.5	-0.015	0.483	$C_{Cp}$	1.957	0.013
${\overset{―}{V}}_{Vp}$	26.25	0.014	0.475	$C_{Vp}$	2.366	0.016

### 6.5. A.5 Multi-start gradient-descent baseline

The optimiser is Adam [53] applied to J(θ) on the unit cube, run from 64 independent restarts of 200 steps each, with the residual scale of Eq. 21 set to $\sigma_{i}=0.02\;|t_{i}|$, one settling run per objective evaluation and a gradient tolerance of 10^–4^. Restarts are seeded from the 64 lowest-*J* points of a 1024-point Latin-hypercube pool: a raw sample over the full safeguard box places many starts in the region where the explicit integration diverges, from which no gradient information is available, and one cheap screening pass avoids spending a descent on them. The cube is mapped logarithmically, so a fixed step is a fixed fractional change in each parameter; under the linear map the optimum sits at very different relative positions within each parameter’s own range, and the compliances with small optima start one step away from the lower wall. Each restart carries its own learning rate, halved whenever *J* stops improving over a 50-step window, and a restart may only be retired once its worst relative error is actually small, and a restart still above tolerance at the learning-rate floor is frozen and reported as stalled rather than silently counted as converged. None were, in this run.

Gradients are central finite differences rather than automatic differentiation. This is a property of the model, not of the tooling: the valve and systole switches are expressed as conditionals, and the inactive branch of such a conditional poisons the reverse-mode gradient with non-finite values at parameter values far from the solution. All 64 descents are advanced in lockstep so that the whole finite-difference stencil, 64×(1+32) points per step, is evaluated in a single vectorised solve; a per-restart optimiser would serialise the same work. The run cost 424 297 forward solves, or 9594 s of wall-clock time, for all 64 restarts together.

The outcome is reported in Table 10 and is the reason this appendix exists. The best restart is excellent, reaching every target to within 0.04 %, better than any of the other three. Only 29 of the 64 restarts reach the targets at all. The remaining 35 come to rest in a distinct spurious basin, at roughly half the systemic arterial resistance and with a correspondingly inflated unstressed arterial volume, where the worst target is missed by up to 18 %. This is what inflates the standard deviations in Table 10: they measure disagreement between restarts, not the precision of any one of them. Fig 17 shows the two groups separating, and shows that they are separated in the parameters, not merely in the residual. Nothing in the optimiser’s own output distinguishes a restart in one basin from a restart in the other; the residual does, but only because the true targets are known here, which is precisely the information a real calibration does not have.

**Table 10:** Multi-start gradient-descent baseline calibration, in the form of Table 6: for each of the sixteen targets, the prescribed value and the signed relative error over all 64 restart optima, with the paired parameter’s mean and standard deviation. The standard deviations measure disagreement between the 64 restarts, 29 of which reach every target to within 2 % and 35 of which stall in a spurious basin at $R_{AsCs}\approx 0.375$ (Fig 17).

Vars.	Targets	Relative Error %	std	Param.	Value	std
${\overset{―}{P}}_{Vs}$	8	0.058	0.493	$C_{Vs}$	59.498	0.669
${\overset{―}{P}}_{Cp}$	11.5	1.829	4.478	$R_{ApCp}$	0.150	0.037
${\overset{―}{P}}_{Cs}$	37.5	1.829	2.288	$R_{CsVs}$	0.731	0.027
$P_{As}^{\max}$	85	-5.264	6.504	$R_{AsCs}$	0.576	0.217
*SV*	19.23	-1.105	1.858	$E_{Hl}$	15.827	2.493
$P_{Ap}^{\max}$	22.5	-0.060	0.905	$E_{Hr}$	3.927	0.289
$PP_{As}$	35	2.370	2.895	$C_{As}$	0.249	0.082
$P_{Ap}^{\min}$	13.5	-2.516	6.332	$C_{Ap}$	1.035	0.379
${\overset{―}{V}}_{As}$	97.5	3.337	4.611	$V0_{As}$	96.624	25.340
${\overset{―}{V}}_{Ap}$	26.25	0.457	1.308	$V0_{Ap}$	7.994	8.573
${\overset{―}{V}}_{Hl}$	15	0.115	0.653	$e_{Hl}$	0.454	0.017
${\overset{―}{V}}_{Hr}$	15	0.203	0.517	$e_{Hr}$	0.262	0.005
${\overset{―}{V}}_{Cs}$	37.5	0.100	0.403	$C_{Cs}$	0.985	0.013
${\overset{―}{V}}_{Vt}$	30	0.091	0.767	$C_{Vt}$	4.477	0.022
${\overset{―}{V}}_{Cp}$	22.5	0.078	0.775	$C_{Cp}$	1.926	0.073
${\overset{―}{V}}_{Vp}$	26.25	0.121	0.529	$C_{Vp}$	2.333	0.093

**Fig 17:**
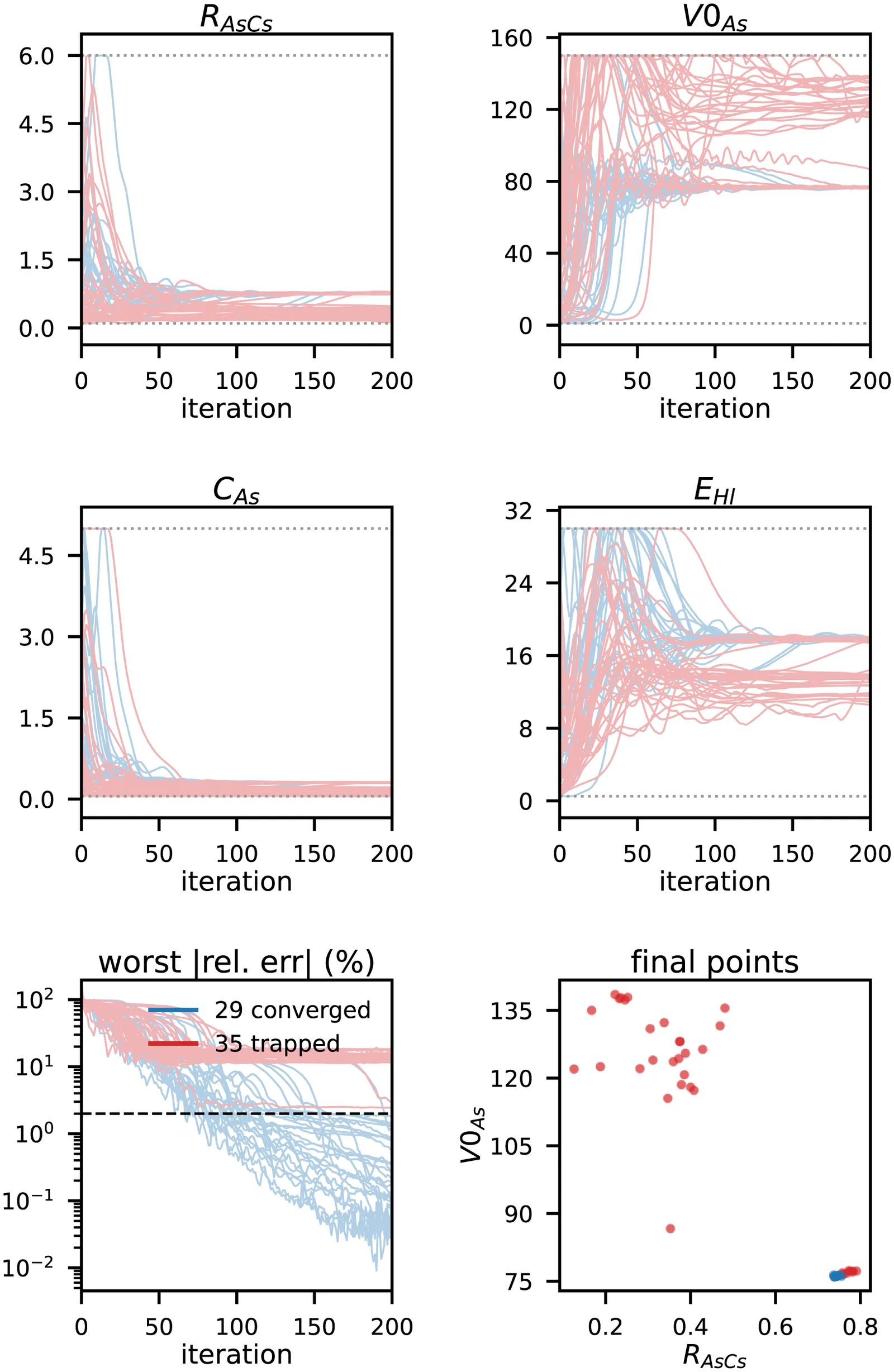
Restart histories of the multi-start gradient-descent baseline, coloured by outcome: blue for the 29 restarts that reach every target to within 2 %, red for the 35 that do not. Panels 1-4 show the four parameters on which the two groups separate, with the prior bounds dotted, panel 5 each restart’s worst relative error against the 2 % threshold, panel 6 the final positions, in which the groups appear as disjoint clusters.

### 6.6. A.6 Calibration stages of the two control laws

The linear law raises the gain once with the controllers active, from a multiplier of 0.1 to 0.25. The cubic law escalates four times, from 0.01 to 10 (Table 11). Under the linear law the population holds its sampled spread through the first multiplier and converges during the second. Under the cubic law it holds that spread through the first two escalations, converges at the third and moves no further at the fourth (Fig 18).

**Table 11:** Calibration stages of the two control laws, in order. The gain multiplier scales the gain of the active law, $a_{L}$ for the linear law and $a_{C}$ for the cubic; *off* is a settling stage. Each run is one 10 s forward solve, so the run column is the cost of the stage. Stages that integrate no runs are omitted.

	Gain multiplier	Runs	Simulated time (s)
Linear 1	off	10	100
Linear 2	0.1	50	500
Linear 3	0.25	300	3000
Linear 4	off	10	100
Cubic 1	off	10	100
Cubic 2	0.01	25	250
Cubic 3	0.1	25	250
Cubic 4	1	400	4000
Cubic 5	10	200	2000
Cubic 6	off	10	100

**Fig 18:**
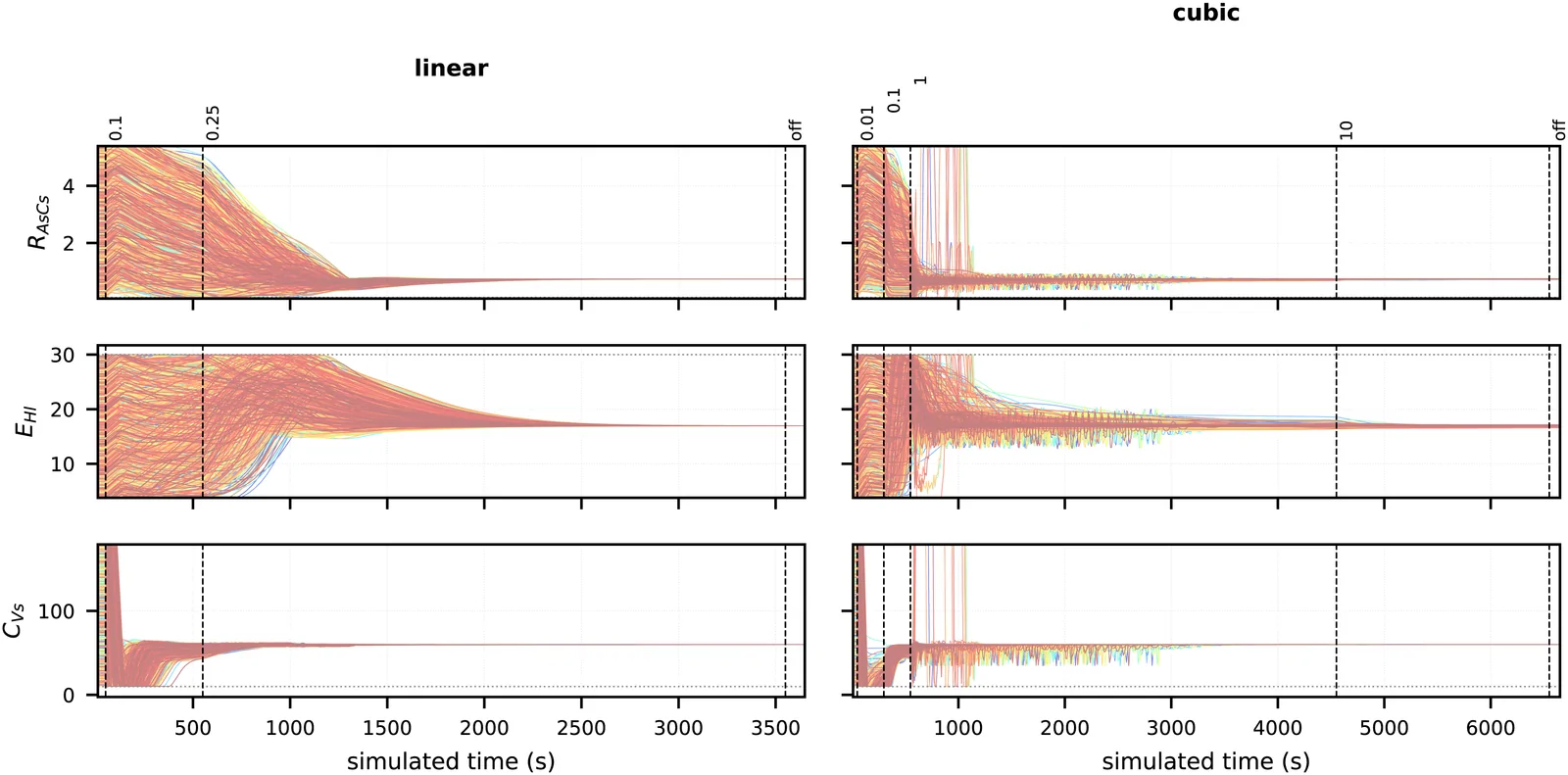
Trajectories of three calibrated parameters over the whole calibration, one line per run, under each control law. Dashed lines mark the stage changes of Table 11, labelled with the gain multiplier each stage opens; *off* is the closing settling stage. Dotted lines are the prior box. The axis is the simulated time each trace covers, 3650 s under the linear law and 6650 s under the cubic.

### 6.7. A.7 Calibration example
