# Supplementary material for: A physiologically constrained calibration framework for cardiovascular models applied to a paediatric synthetic sepsis population
Source: PLoS Comput Biol. 2026 Oct 7;22(10):e1014847. doi: 10.1371/journal.pcbi.1014847 (PMC13705617; doi:10.1371/journal.pcbi.1014847)
Supplement: Supplementary file 1 [file pcbi.1014847.s001.pdf]

Manuscript: *A Physiologically Constrained Calibration Framework for Cardiovascular Models Applied to a Paediatric Synthetic Sepsis Population*
Authors: M. T. Cabeleira, S. Ray, N. C. Ovenden, V. Diaz-Zuccarini
Journal: PLOS Computational Biology
Decision: Major revision

Dear Editor,

We thank you and the two reviewers for the time and care taken over our manuscript, and we are grateful for the encouraging assessment of the work. Reviewer 1 found the paper 'interesting and well written', and Reviewer 2 considered the direction of the work 'interesting for tuning patient-specific solutions and clinical digital-twin applications' and the methodology 'ambitious', recommending publication in PLOS Computational Biology once the comments have been addressed. Both reviewers engaged closely with the substance of the method, and their constructive criticism have made the paper substantially better, and we have taken all of them seriously.

Below we reproduce every comment verbatim and answer it in turn. Changes in the manuscript are marked in the accompanying tracked-changes PDF.

Yours sincerely,
M. T. Cabeleira, on behalf of all authors

Reviewer #1

R1-C1

The validation is entirely performed using synthetic measurements. There is no comparison against real pediatric ICU recordings, nor against an independent calibration method (e.g. MCMC or multi-start gradient descent) applied to the same targets. Without such an external reference, claims of 'low residual error' and 'physiological consistency' are difficult to interpret as evidence of correctness. The authors should add at least one comparison against an established optimization method on an identical calibration task, and discuss the challenges they anticipate when moving to real patient data.

Response:

We agree with the reviewer's comments. On the absence of real patient data we have now highlighted further (in the Title and abstract) that the whole study used synthetic, in silico, data. Further, in the Limitations we state that the cohorts were built from literature-derived target ranges and were not compared against patient recordings. Here we also explore the key challenges, for example, when real patient data is used, namely uncertainty on each value, targets recorded at different times and sampling intervals and mutually incompatible target sets.

On the external method validation we have extended the convergence section to include a comparison of the calibration method in the linear and cubic variants with the more conventional MCMC (emcee implementation) and Gradient Descent (Adam steps) approaches. The shared setup is given in Section 2.5 and the two implementations in Appendices A.4 and A.5. The Gradient Descent methodology is the least reliable on this calibration task as 35 of its 64 restarts stall in local minima, leaving a median restart error of 11.54%.

The three methods converge to the same region of parameter space, and the embedded controllers reach it at the lowest computational cost: 370 solves and 49.6 s of wall-clock for the linear law, 670 solves and 89.8 s for the cubic, against the 192 000 solves of the MCMC population (128 walkers over 1500 steps). This is reported in Section 3.1, Table 5 and Figure 8.

Lines affected: Revised manuscript, lines 772-807 (Section 2.5), 820-920 (Section 3.1, with Table 5), 1043-1079 (Discussion, *Convergence analysis*), 1300-1302 and 1349-1358 (Limitations), 1578-1619 (Appendix A.4) and 1620-1671 (Appendix A.5). Figures 7 and 8 are new.

R1-C2

The central claim that EGD 'converges to a single physiological solution' needs a more careful statement. The Introduction motivates the work precisely by the fact that multiple parameter combinations reproduce the same hemodynamic targets (i.e. the presence of non-identifiable combination of parameters). EGD does not resolve this non-identifiability intrinsically; it removes it by design, through the fixed one-to-one target/parameter assignment of Figure 6, and by leaving selected compartments uncontrolled. The uniqueness is therefore conditional on these modelling choices. The authors should make this explicit and show how sensitive the calibrated solution and the cohort-level conclusions are to alternative controller assignments.

Response:

We agree that the solution uniqueness is imposed by the design of the calibration problem. By extending the set of targets to 16 whilst using 16 parameters to attain this result, an over-determined system emerges where only one solution is possible. This is now explicit in the manuscript. The Abstract and the Conclusion state the uniqueness as the single solution admitted by an over-determined target set, the Solution uniqueness paragraph of the Discussion has been rewritten around that set, Convergence analysis now reports the same solution from any admissible starting point, and Section 2.3.2 lists the parameters left outside the calibrated set.

The assignment of the parameter/observation pairs in Figure 6 was however done according to the influence each parameter has in the observations and the need to find 16 pairs that would all individually work. For this, we made a sensitivity study, given in Figure R1. Each column is a ladder of 16 calibrations in which that target alone is stepped across a multiple of its reference value (0.75-1.25), every other target and the initial state are held, and the model is recalibrated for the new target set. Each entry is the elasticity $\varepsilon = \partial \ln p / \partial \ln y^*$ of one parameter under that ladder. The diagonal of the figure holds the sixteen pairs of Figure 6: each observation was given the parameter of highest elasticity available, with the signs along that diagonal matching the signs of Table 4.

In the figure, we can see that volumes are mostly affected by the compliances and the unstressed volumes, and by little else, so those parameters go to the volume targets first. With them committed, the resistances carry the strongest remaining influence on the pressures. As we now still have systolic compliances of the heart (as elastances) available and these greatly affect the SV, the left ventricle was used to control it. Leaving the other compliances available to modulate the systolic and diastolic pressures. Any parameter with a high elasticity for an observation could be used to target it.

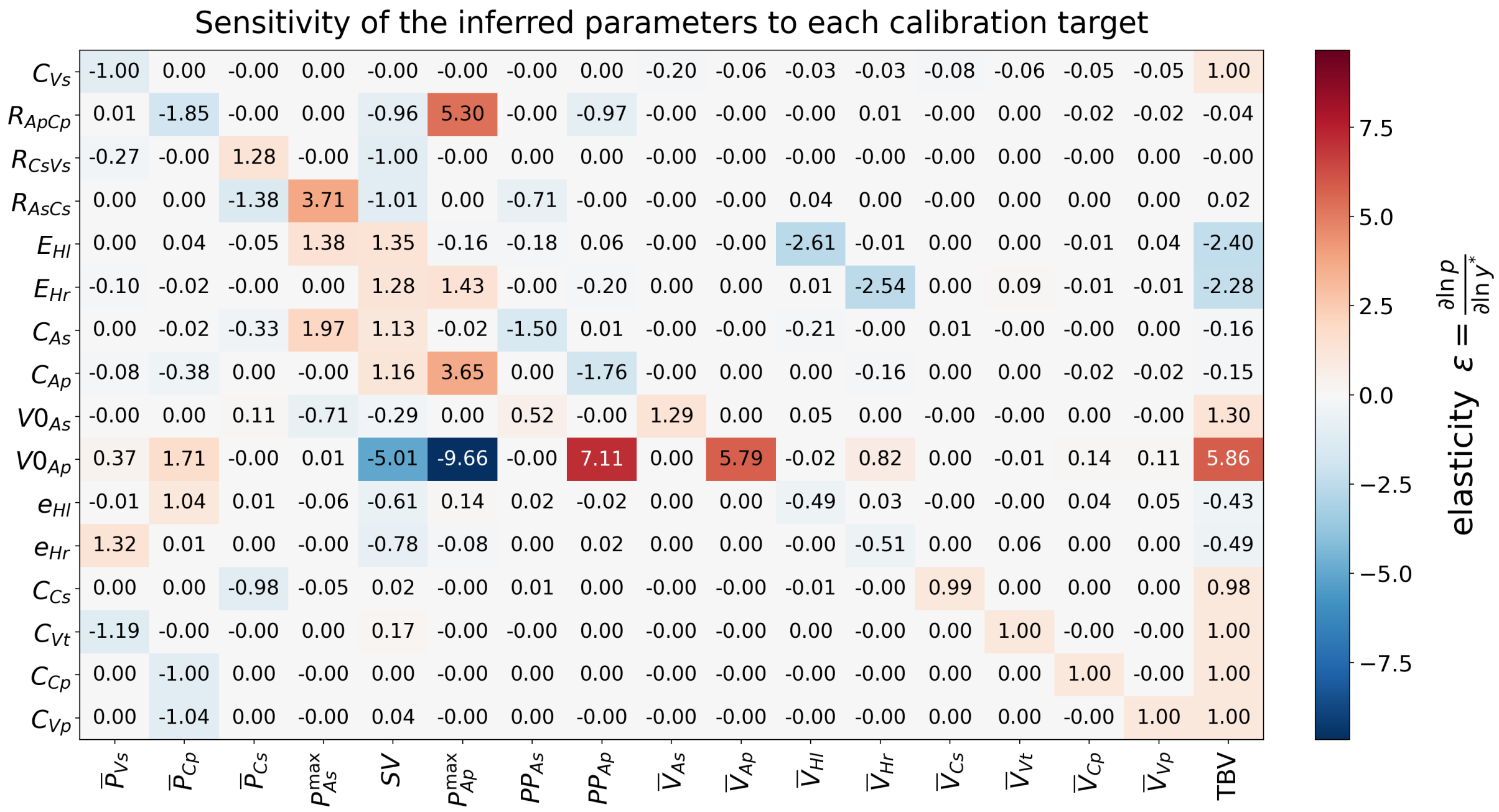

Figure R1. Sensitivity of the sixteen calibrated parameters (rows) to each calibration target (columns), as the elasticity $\varepsilon = \partial \ln p / \partial \ln y^*$. Each column is one ladder of 16 values in which that target alone is stepped across a multiple of its reference value, every other target and the initial state are held, and the model is recalibrated by the unchanged method at every step. A column with more than one large entry is a target over which more than one parameter has authority. The last column steps the total blood volume and the eight compartment volume targets together, holding the reference partition.

Lines affected: Revised manuscript, lines 16 (Abstract), 684-693 (Section 2.3.2), 1075-1077 (Discussion, *Convergence analysis*), 1080-1113 (Discussion, *Solution uniqueness*) and 1368-1370 (Conclusion).

R1-C3

The model discuss by the authors is composed of resistors and capacitors, but there are no inductance elements to simulate effects due to blood inertia (typically inductors are assigned to the aortic and pulmonary arterial compartments). The authors should discuss if any changes in the proposed tuning approach would be need for RLC-based lumped parameter

models.

Response:

To address this comment we have repeated the calibration procedure with the aortic valve changed to an inertial element (the flow leaving the left ventricle has equation, $L\,\mathrm{d}Q/\mathrm{d}t = P_{Hl} - P_{As} - QR$) and the aortic inductance L was swept over 64 geometrically spaced values from 0.0005 to 0.1 $\mathrm{mmHg\,s^2/mL}$. Each value was calibrated to the same physiological targets as in 3.1, with the parameter/target assignment of Figure 6. No change to the calibration procedure was needed and the calibration procedure is compatible with models with inductance. All 64 calibrations also converge with very low error (see Figure R2).

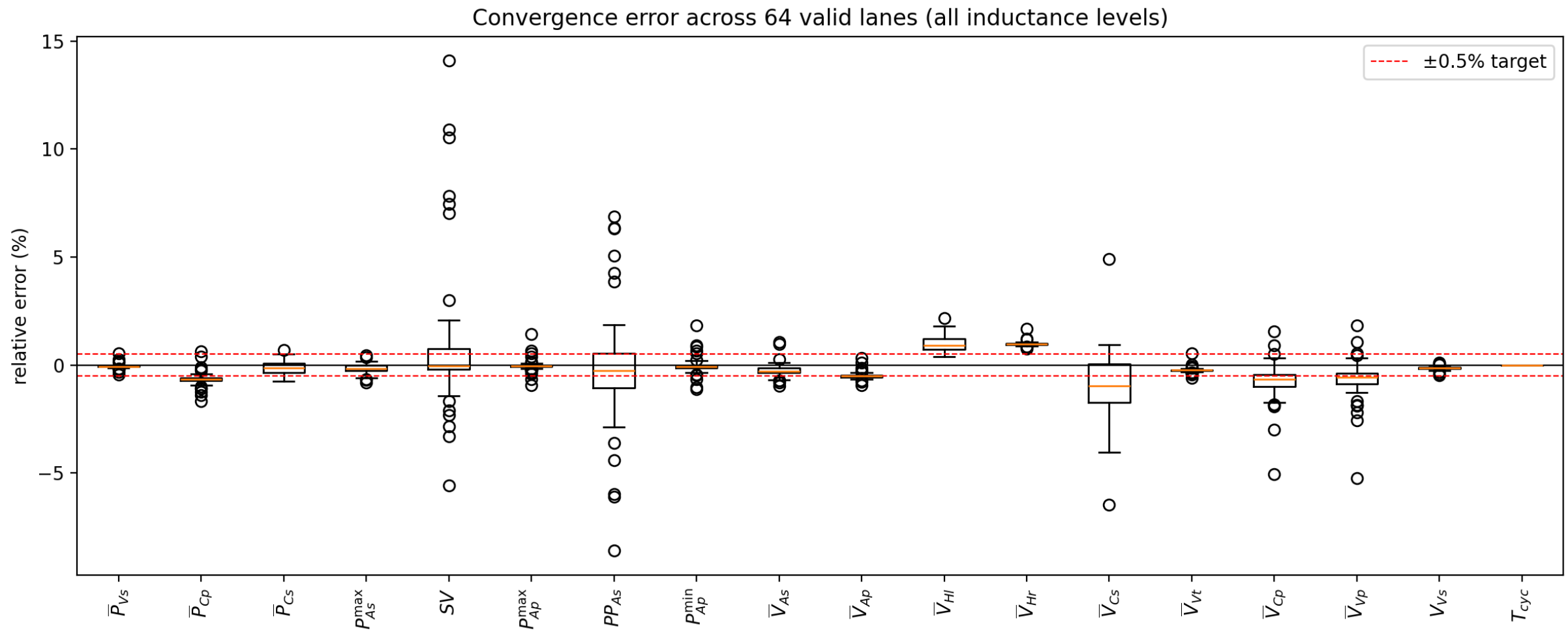

Fig. R2. Relative error of every calibration target at the end of calibration, over the 64 calibrations of the aortic-inductance sweep (L_{HlAs} from 0.0005 to 0.1 $\mathrm{mmHg\,s^2/mL}$). Each box is the interquartile range over the calibrations, the whiskers reach 1.5 times that range and the calibrations beyond them are drawn individually. The dashed lines mark ±0.5%. The largest error over the targets has a median of 1.58% across the sweep, against 1.79% for the unmodified model, and reaches 14.1% for SV.

In Table R1 the effect of the inductance sweep in the parameter space is shown. As can be seen the inductance sweep shows that for different inductance values the same target space generates different parametrization of the vessel that has the inductance (C_{As} 0.200 to 0.549, $V0_{As}$ 62.8 to 84.2 and R_{AsCs} 0.635 to 0.721, spanning 113%, 28.0% and 11.7% of their published values) and at the ventricle (E_{Hl} 8.62 to 17.0 and e_{Hl} 0.423 to 0.603, spanning 49.2% and 40.9%).

A single solution is needed to find out whether a calibration algorithm reaches it and does not stall in a local minimum, and it is what lets the results of the calibrated populations be compared with one another. A model carrying an inductance can therefore still be calibrated but at a fixed value of L.

Parameter	Paper value	Min	Max	Max − Min	Range %
C_{Vs}	60.0	59.8	60.1	0.272	0.454
R_{ApCp}	0.165	0.163	0.166	0.00351	2.12
R_{CsVs}	0.708	0.701	0.708	0.00729	1.03
$\boldsymbol{R_{AsCs}}$	**0.740**	**0.635**	**0.721**	**0.0868**	**11.7**
$\boldsymbol{E_{Hl}}$	**17.0**	**8.62**	**17.0**	**8.34**	**49.2**
E_{Hr}	3.92	3.91	3.98	0.0684	1.75
$\boldsymbol{C_{As}}$	**0.311**	**0.200**	**0.549**	**0.350**	**113**
C_{Ap}	1.18	1.18	1.20	0.0251	2.13
$\boldsymbol{V0_{As}}$	**76.2**	**62.8**	**84.2**	**21.4**	**28.0**
$V0_{Ap}$	4.53	4.24	4.62	0.387	8.54
$\boldsymbol{e_{Hl}}$	**0.439**	**0.423**	**0.603**	**0.180**	**40.9**
e_{Hr}	0.257	0.254	0.257	0.00317	1.23
C_{Cs}	1.01	0.950	1.05	0.0966	9.60
C_{Vt}	4.44	4.44	4.46	0.0161	0.363
C_{Cp}	1.96	1.88	2.00	0.121	6.18
C_{Vp}	2.37	2.26	2.42	0.157	6.62

Table R1. Range of each calibrated parameter over the aortic-inductance sweep (64 converged lanes, L_{HlAs} from 0.0005 to 0.1 $\text{mmHg}\,\text{s}^2/\text{mL}$), against the value the same calibration gives for the unmodified model. Each value is read at the end of its calibration; the minimum and the maximum are taken over the sweep and need not fall at its ends. The last column, Range %, is the range as a percentage of the paper value, and the rows above 10.0% of it are in bold.

Lines affected: Revised manuscript, lines 1332-1338 (Limitations), where the sentence on inertial effects now states that the simplification carries no impact on steady-state calibration other than reparameterisation. No other text was written for this comment.

R1-C4

> *No formal analysis of the controller dynamics is provided. Additionally, the selected cubic law is introduced heuristically, but it is not clear why a different law would not be appropriate. Also, there is no stability or convergence argument for the sixteen coupled nonlinear controllers acting simultaneously and the statement that the scheme prevents 'convergence to partial or local minima' is asserted but not demonstrated.*

Response:

On the dynamics of a single controller, Section 2.3.1 now derives two properties from Eq. 16. Its equilibria are exactly the calibrated states: $dY/dt = 0$ requires $\sigma_{\text{rel}}(a_C\sigma_{\text{rel}}^2 + a_L) = 0$, and the bracket cannot vanish when the two gains carry the same sign, leaving $\sigma_{\text{rel}} = 0$ as the only root. The Jacobian is non-zero under the linear law and the residual decays exponentially; under the cubic law it vanishes at the target and the residual decays as $t^{-1/2}$.

In the revised Section 3.1 we have now also run the convergence analysis with the linear version of the controller law and found that the system converges faster to the solution with an even lower spread than the cubic law. The cubic law is also more unstable than the linear law with the all linear runs converging to the same solution whereas in the cubic test, 27 runs did not converge. We have added Appendix A.6 where we show the effect of the controller gain parameters on the parameter variation. The higher the gain values, the faster the parameters change. As the cubic law derivative vanishes when close to error = 0, we have increased the gain value in steps but if this is done when the error is still high, the system can diverge due to a high derivative value.

On the local minima claim, we now show that the GD does indeed stall in regions that are still off target. The MCMC also suffers from the same issue with the walkers having to be 'rescued' and resampled 778 times so that no local minima get propagated to the end of the simulation.

So despite the problem only having one solution by construction, only the EFC methodology managed to avoid getting stuck in local minima.

Lines affected: Revised manuscript, lines 597-608 (Section 2.3.1), 772-807 (Section 2.5), 820-920 (Section 3.1), 1080-1113 (Discussion, *Solution uniqueness*) and 1672-1683 (Appendix A.6). No text was written for this comment alone.

R1-C5

> *The model contains no autonomic/baroreflex regulation, despite autonomic dysfunction being a defining feature of septic shock and despite the 'ANS-Symp.' regulatory loop being included in Figure 1. The authors should comment on the validity of their modeling approach in light of this approximation.*

Response:

We agree that this is an important aspect of the work and have now added a section in the discussion named 'Autonomic regulation'. The figure itself, its caption and the Introduction sentences that mention autonomic regulation are addressed in our response to comment 9.

The purpose of the EFC is to establish the operating point of the model, the parameter set and the initial conditions from which any subsequent simulation departs. That operating point is inferred from a steady-state clinical snapshot in which the autonomic contribution has already influenced the physiology being measured. The effect of the autonomic nervous system on heart rate is read from the monitor and enters the model as an input, since HR is prescribed for each virtual subject (our response to comment 7). Its effect on the peripheral resistances and on cardiac contractility is present in the pressures and flows we calibrate against, so it is absorbed into the calibrated resistances, compliances and ventricular elastances.

Autonomic dysfunction is therefore carried by the calibrated parameters. The low systemic resistances of the warm shock population and the reduced systolic elastance of the cold shock population are the haemodynamic signatures of the compensatory responses drawn in Figure 1, recovered as parameter values in Section 3.3. An intact reflex would not permit resistances that low.

Lines affected: Revised manuscript, lines 1270-1297 (Discussion, *Autonomic regulation*) and 165-168 (Introduction). The caption of Figure 1 is covered under comment 9.

R1-C6

> *Total blood volume and its distribution across compartments are fixed to a common reference for all cohorts (Section 2.5, Limitations). The authors acknowledge this, but since inflammatory volume loss and redistribution are central to the haemodynamics of sepsis, fixing TBV partition seems to remove one of the most clinically relevant degrees of freedom. Some quantification of how strongly this assumption biases the inferred shock parameter distributions would be valuable.*

Response:

We acknowledge the reviewer's concerns on the fixed distribution of blood volume throughout the population. On quantifying how far that assumption biases the inferred parameters we can look at the sensitivity study given with our response to comment 2. Here eight of its columns are ladders of the compartment volume targets, and the last steps the total blood volume with the reference partition held fixed. Moving volume between compartments is taken up locally, by the compliances. Each of the four venous and capillary volume targets are mostly affected by their own compliance (or unstressed volume for the main arteries), with every other parameter being

mostly unaffected other than the systemic venous compartment that compensates the volume shift.

Neither the total blood volume of a subject nor its distribution across the circulation can be measured, as we set out in Section 2.1, *Total Blood Volume (TBV)*. Whatever partition we impose is therefore an assumption, and holding the same one for every subject is what keeps the calibrated parameter sets directly comparable between individuals and across the three cohorts.

R1-C7

> *The treatment of heart rate in the calibration is unclear. Cardiac output is controlled only through stroke volume via the left-ventricular elastance E_{Hl} (group 4, Figure 6), and $CO = SV{\cdot}HR$. Table 2, however, prescribes HR target ranges, and Figure 6 lists no HR controller. Is HR a fixed input drawn from the target ranges, or is it calibrated? Please clarify where the HR target enters the procedure and how CO targets are met when HR is itself constrained.*

Response:

Heart rate is a prescribed input to the model, and no controller acts on it, so Figure 6 lists none. For each virtual subject HR is sampled once from the range given in Table 2 or, in a real-world setting, read directly from the patient monitor. It enters the equation system through the cycle duration $HC = 60/HR$ (Section 2.2, *Cycles: Heart rate modelling*).

Table 2 lists HR among the sampled quantities that define a subject. We have now marked that row with a ¶ symbol and the caption now states that HR is used as an input to the model.

Because HR is fixed, the prescribed CO target is calculated at initialisation as an equivalent stroke-volume target, $SV^{\star} = CO^{\star}/HR$. The single group-4 controller drives SV to $SV^{\star}$ through the left ventricular contraction elastance E_{Hl}, and reaching $SV^{\star}$ at the prescribed heart rate reproduces $CO^{\star}$ exactly.

Lines affected: Revised manuscript, lines 644-651 (Section 2.3.2, group 4). The HR row of Table 2 and its caption carry no line number.

R1-C8

> *The reported per-cohort simulation protocol (2200 s of simulated time, 200 s transient, $\Delta t = 0.0005$ s) implies a substantial computational cost per virtual subject. Since the stated long-term goal is patient-specific digital twins in acute care, the authors should report wall-clock cost per calibration and discuss whether the approach is fast enough to be clinically useful, and how it scales relative to the optimization methods it is meant to replace.*

Response:

We agree that these timings should be measured and discussed in the manuscript. The simulation protocol has also been updated: the single 2200 s run was replaced by a staged schedule of 10 s forward solves, given in Section 2.5, so that one calibration is 370 solves under the linear control law and 670 under the cubic law, 3700 s and 6700 s of simulated time. The cost is reported in the new Appendix A.3, *Timing and integrator comparison*, with a pointer in Section 2.4.

On a laptop CPU (Intel Core Ultra 7 155H, 16 cores, 32 GB RAM, double precision, CPU only), one 10 s forward solve costs 0.134 s of wall-clock time, so a full patient calibration takes 49.5 s under the linear law and 89.8 s under the cubic law used for the sepsis cohorts. The explicit Euler scheme is what brings the cost down to these times. Appendix A.3 shows that RK4 and Dopri5 reach the same calibrated solution to within 1.3 % on every parameter, at 215.5 s and 4284.2 s for the same calibration, so the accuracy of the higher-order schemes is available here at 4.4 and 86.5 times the cost.

On the same machine the MCMC sampler takes 9077 s and the gradient descent methodology 9594 s, making the embedded controllers faster by factors of 183 and 194 against the linear law, and 101 and 107 against the cubic.

Lines affected: Revised manuscript, lines 753-761 (Section 2.4) and 1547-1577 (Appendix A.3, with Table 8).

R1-C9

> *Figure 1 mixes mechanisms that are in the model (resistance, compliance, cardiac output, stroke volume) with mechanisms that are not (autonomic vasoconstriction/vasodilation). I would revise this schematic so it distinguishes clearly between physiological background and the components actually implemented, and add explicit references to the corresponding Sections/Equations.*

Response:

On the separation between physiological background and implemented components, we have now rewritten the caption of Figure 1 and the sentence that cites it. Figure 1 sits in the Introduction and explains how the warm and cold shock phenotypes arise. The caption now states that the autonomic responses drawn there explain the observed heart rate, pressures and flows, that no baroreflex loop is implemented in the model, and that the model schematic is Figure 2. We have also dropped autonomic regulation from the list of quantities the modelling framework represents in the closing sentence of that part of the Introduction.

On the Section and Equation references, the nodes of the figure, blood pressure, resistance, cardiac output, heart rate, stroke volume, end-diastolic and end-systolic volume, are model variables defined in Sections 2.2 and 2.3. The autonomic pathways have no corresponding Section or Equation, and their omission is justified in the Autonomic regulation paragraph of the Discussion. The artwork itself is unchanged.

Lines affected: Revised manuscript, lines 139-141 and 165-168 (Introduction). The caption of Figure 1 carries no line number.

R1-C10

> *The leaving-uncontrolled of the thoracic veins (Pvt) and pulmonary veins (Pvp) is justified only qualitatively ('negligible pressure drops'). Given that nine compartments but only sixteen of the available parameters are controlled, a brief sensitivity check on whether this choice affects the calibrated venous/capillary distribution would help substantiate the claim.*

Response:

The thoracic and pulmonary venous compartments are controlled in volume. Their cycle-averaged volumes $\overline{V}_{Vt}$ and $\overline{V}_{Vp}$ are two of the 16 calibration targets, reached through the compliances C_{Vt} and C_{Vp} (group 3 of Section 2.3.2), so the calibrated venous and capillary distribution is the blood distribution we prescribe. What is left uncontrolled is R_{Vs_Vt}, R_{Cp_Vp} and the four valve resistances at the heart inlets.

A resistance sets only the pressure drop between the two compartments it connects, $\Delta P = QR$. Figure 12 shows this for the systemic capillary bed, where the ratio $R_{AsCs}/(R_{AsCs}+R_{CsVs})$ fixes the fraction of the drop taken upstream and downstream of the capillaries, and the 'Population Parameters' paragraph of the Discussion interprets that ratio across the three calibrated cohorts. A controller on R_{Vs_Vt} or R_{Cp_Vp} would give the same handle on the venous drop. Targeting it needs a measurement we do not have: central venous pressure is already assigned to P_{Vs} (group 8), and the ICU data carry no thoracic or pulmonary venous pressure from which a drop could be prescribed.

Lines affected: Revised manuscript, lines 684-693 (Section 2.3.2), which name the six uncontrolled resistances, state that every compliance, elastance and unstressed volume is calibrated, and give the two additions that would bring either venous compartment into the calibration.

R1-C11

> *Section 2.4. Use of the explicit Euler scheme is quite unusual for lumped parameter models which may exhibit stiffness due to perfect unidirectional valves and due to changes in overall RC constants for compartments, following opening and closing of valves. The authors should report a comparison with, e.g., higher order numerical integrators with adaptive time stepping (e.g. RK4-5).*

Response:

We repeated the same staged calibration under three integrators, the explicit Euler scheme, a fixed-step fourth-order Runge-Kutta method (RK4), and the adaptive Dopri5 (an RK4(5) pair with adaptive time stepping), from identical initial conditions and the same physiological targets. All three reach the same calibrated solution. The maximum relative error against the targets is 2.7% for every integrator and the calibrated parameters agree to within 1.3%. The explicit Euler discretisation at $\Delta t = 0.0005$ s is therefore accurate, and it reaches that solution 4.4 times faster than RK4 and 86.5 times faster than Dopri5. The comparison is reported in a new Appendix subsection, 'Timing and integrator comparison', with Table 8 (residual error, cross-integrator parameter agreement, wall-clock cost) and Figure 15 (per-target relative error), pointed at from Section 2.4.

Each perfect unidirectional valve makes the right-hand side discontinuous. The adaptive step-size controller shrinks its step at every valve event to keep its error estimate inside tolerance, so Dopri5 spends its cost resolving valve switching and gains no accuracy.

Lines affected: Revised manuscript, lines 753-761 (Section 2.4) and 1547-1577 (Appendix A.3, with Table 8). Figure 15 is new

R1-C12

> *Figure 8: please state explicitly what the colors correspond to.*

Response:

We thank the reviewer for spotting this. The three violin pairs in each panel are the three cohorts, normal physiology in green, warm shock in red and cold shock in blue, and the same colour code is used for the cohorts in every population figure of the paper. The caption of Figure 8 now states this.

Lines affected: The figure is Figure 9 of the revised manuscript, on page 15

Syntax and references

R1-M1

> *Introduction: please review the sentence 'Some notable examples in literature that do provide solutions to the parametrization of ODE models usually also very much simplified [22, 23]'.*

Response:

Corrected.

Lines affected: Revised manuscript, lines 66-70.

R1-M2

Introduction: 'for the the model'.

Response:

Corrected.

Lines affected: Revised manuscript, line 100.

R1-M3

Section 2.3.1: 'replaced with any another monotonic'.

Response:

Corrected. The phrase now reads 'replaced with another monotonic function'.

Lines affected: Revised manuscript, lines 576-577.

R1-M4

Page 21: Appendix A.2: 'The systemic arterial pulse pressure variable appear', the sentence appears to be incomplete.

Response:

Corrected; the incomplete sentence has been removed and the systemic arterial pulse pressure variable is now defined explicitly.

Lines affected: Revised manuscript, lines 1491-1495 (Appendix A.2). The retention update terms sit inside display equations, which carry no line number.

R1-M5

Page 1 (Introduction): the sentence 'In Djoumessi et al. The addition of autonomic control to these models allows the study of the response to hypercapnia [14, 15, 16]. . . ' is a broken fragment, and the citation does not appear to match the named author.

Response:

Corrected. The fragment 'In Djoumessi et al.' was a leftover from an earlier draft and has been deleted; the sentence now reads 'The addition of autonomic control to these models allows the study of the response to hypercapnia'. The citations attached to the sentence are correct as they stand: Albanese et al., Albanese et al. and Fernandes et al. support the hypercapnia response, and the citation of Djoumessi et al. in the following paragraph is also correct, where it supports the observation that published parameter sets are commonly reused without further examination of the model behaviour under different parameter regimes.

Lines affected: Revised manuscript, lines 52-53.

R1-M6

Reference [40] (Khonsary, Guyton and Hall): the DOI is rendered with unescaped LaTeX, `10.4103/sni.sni{\_}327{\_}17` *— should be* `10.4103/sni.sni_327_17`.

Response:

Corrected.

Lines affected: Revised manuscript, line 1825; the entry is reference [41] of the revision.

R1-M7

> *Reference [30] (Shankar-Hari et al.): 'Developing a newdefinition and assessing newclinical criteria for Septic shock' — missing spaces: 'new definition' and 'new clinical'.*

Response:

Corrected.

Lines affected: Revised manuscript, lines 1785-1786; the entry is reference [31] of the revision.

R1-M8

> *Reference [29]: author names are run together — 'Christopherwarren Seymour' and 'Tomvan Der Poll' should be 'Christopher Warren Seymour' and 'Tom van der Poll'.*

Response:

Corrected.

Lines affected: Revised manuscript, lines 1780 and 1782; the entry is reference [30] of the revision.

Title, abstract, references

R1-M9

> *TITLE: maybe use 'applied to Pediatric Sepsis' instead of 'applied in Pediatric Sepsis'? just a suggestion.*
> *ABSTRACT: appropriate.*
> *REFERENCES: Appropriate in scope and coverage.*

Response:

We thank the reviewer for this suggestion, which we have adopted. The title now reads 'A Physiologically Constrained Calibration Framework for Cardiovascular Models Applied to a Paediatric Synthetic Sepsis Population', which also carries the 'synthetic' qualifier requested by Reviewer 2.

Lines affected: Revised manuscript, lines 1-3.

Reviewer #2

R2-C1

The work is based on a numerical model using a randomly sampled virtual population as an internal validation but is not validated on an external sepsis real dataset. Many sampled internal variables are unobserved, which may result in numerous non-physiological combinations and missed joint dependencies. Therefore this is not a pediatric sepsis application in the clinical sense, so I would suggest the authors to provide a disclaimer about this in the text, or provide an external clinical validation to justify the virtual cohort sampling. I believe that the title is also a bit misleading, as the reader expects to see some clinical results on pediatric sepsis. I would recommend the 'application' part to not be in the title, or to be explicit about its in-silico nature.

Response:

We acknowledge that the study is entirely computational and we offer no external clinical validation. We have now made the in-silico nature of the work explicit in the manuscript.

- The title now reads 'A Physiologically Constrained Calibration Framework for Cardiovascular Models Applied to a Paediatric Synthetic Sepsis Population'. The paediatric sepsis setting is what fixes the structure of the target set and the two shock phenotypes, so dropping the application from the title would leave the reader without the reason the calibration problem takes this form.
- The Abstract now states that the framework is demonstrated in silico and that the cohorts are generated from literature-derived target ranges (validated by a Paediatric ICU doctor and second author of this paper).
- The Introduction now states that the populations span the haemodynamic scenarios reported for paediatric sepsis, with admissible target ranges taken from the clinical literature.
- Limitations now opens with the absence of clinical validation: the cohorts were built from literature-derived target ranges and never compared against patient recordings. The ranges come from separate clinical studies, each reporting its own cohort, so a virtual subject may combine values that are plausible in isolation yet rarely occur together in one child.

The calibration screens the structurally impossible cases, since target combinations that violate the conservation and closure relations of the closed-loop system surface as persistent residuals. Mechanical feasibility is not the same as clinical co-occurrence though, and establishing the latter would need a cohort in which the sixteen quantities are measured concurrently, which no published paediatric study currently reports.

Lines affected: Revised manuscript, lines 1-3 (title), 17-19 (Abstract), 181-184 (Introduction) and 1300-1311 (Limitations).

R2-C2

The authors argue that EGD converges to a unique steady solution, claiming that for any admissible target set, there is a unique physiological solution. This claim relies on qualitative monotonicity of parameter-variable relationship and on the fact that multiple controllers impose simultaneous constraints. However, this does not prove global uniqueness in a coupled nonlinear ODE system. The authors report an empirical convergence experiment with 100 randomized initializations for a single normal target set, but this is not enough to guarantee global behavior. I recommend the general uniqueness claim to be weakened to 'empirical testing cases results' and some additional tests to be included regarding broader target ranges, controller gains and target sets.

Response:

We agree with the reviewer that the unique solution was overclaimed. Uniqueness is imposed by the way the calibration problem is set up: sixteen parameters are calibrated against a set of sixteen targets that over-determines the model, and it is that over-determined target set which admits a single solution. We make no claim of global uniqueness for the coupled nonlinear ODE system. The claim now reads in these terms in the Abstract, in the 'Solution uniqueness' paragraph of the Discussion and in the Conclusion. The convergence experiment has also grown from the 100 randomised initialisations to 1024, drawn by Latin hypercube over the whole prior box and run once under each control law (Section 3.1).

The same target set was given to MCMC and to gradient descent (Section 3.1 and Figure 8). Neither method carries controllers, a parameter/target assignment or the qualitative correlations of Table 4, so neither relies on the structure the reviewer questions. Both recover the solution the controllers reach: MCMC concentrates 98.2% of its walkers within 1.1 times its own posterior median on all sixteen parameters, and every one of the 1024 controller calibrations lies inside that region. Both methods can stall short of the targets. Gradient descent comes to rest away from them on 35 of its 64 restarts, and the MCMC walkers had to be resampled 778 times to stop the stalled regions propagating to the end of the run. A state that leaves targets unmet does not satisfy the calibration, so it is not a second solution.

Lines affected: Revised manuscript, lines 16 (Abstract), 1085 and 1114-1122 (Discussion, *Solution uniqueness*) and 1368-1370 (Conclusion).

R2-C3

> *The authors say that the parameter bounds are introduced 'solely as numerical safeguards' and are chosen wide enough to let the solution space unconstrained. But later on, saturation is used precisely as the condition for infeasibility. Moreover, the authors say that all incompatible target combinations are associated with persistent residuals. Such a diagnostic could be valuable but the threshold that distinguishes infeasibility from slow convergence, poor gain selection, or numerical instabilities should be justified. Adding an experiment of custom cases verifying that feasible targets always have low residuals while infeasible target have high residuals (for fixed model settings) would strengthen the claim.*

Response:

We agree with the reviewer's comment and have now removed from Section 2.4 the sentence claiming that physiological plausibility emerges from the governing equations and their coupling structure alone. The paragraph now states that a calibration whose targets the model can meet comes to rest with every parameter inside its interval, and that a parameter held at a bound has been driven well outside the physiologically plausible range, so the target combination that drove it there is deemed physiologically infeasible. The same correction was also made in the abstract.

Slow convergence, gain selection and numerical error are each bounded in the revised manuscript. Every calibration runs past the point at which its error stops falling, reaching 1% at step 193 of its 370 under the linear law and at step 512 of 670 under the cubic (Section 3.1, Figure 7; Appendix A.6). The two control laws and their stage schedules leave the final error between 0.90% and 1.09% on the same target set, and Euler, RK4 and Dopri5 return the same calibrated parameters and the same residual (Appendix A.3). None of the three holds a parameter at a bound.

Experiments verifying that feasible targets have low residuals while infeasible targets have high residuals are already presented in Figures 9 and 10, on the sepsis population. Figure 9 gives the error distribution of every target across the three cohorts, and the two largest belong to $\overline{V}_{Ap}$

and SV. Figure 10 traces both errors to controller saturation, the positive errors of $\overline{V}_{Ap}$ to $V_{0,Ap}$ held at its lower bound and the negative errors of SV to E_{Hl} held at its upper bound. The third pair, $\overline{V}_{Hl}$ and e_{Hl}, has no controller at a bound, and its errors stay small and fall on both sides of the target.

Lines affected: Revised manuscript, lines 762-771 (Section 2.4) and 22-23 (Abstract).

R2-C4

> *The authors should be more specific on which parameter patterns emerge from the coupled model and which are just expected algebraic results from the prescribed target ranges. Phrases like 'providing physiological insight' should not apply to internal consistency and numerical sanity checks. In the Abstract the authors state that the resulting parameter distributions are consistent with known pediatric sepsis adaptations, including vascular resistance, compliance etc, but this is to be expected by construction of the parameter ranges and acts more as a cyclic confirmation, not a clinical insight.*

Response:

We would like to emphasise that all parameter patterns both emerge from the model and are expected algebraic results of the targets. This is because no parameter set can be fully calculated from Table 2 and need to go through the calibration procedure to be attained. Looking at figure R1 we can see that many parameters influence many variables and every change to one target implies changes on others, even if very small. The exceptions that can be calculated with small errors are the 2 capillary compliances as we can compute the needed pressures and volumes from Table 2 and the volume distribution from Table 3 and calculate $C_x = (\overline{V}_x - V_{0,x})/(\overline{P}_x - P_{\text{Bias},x})$ but no other parameter can be calculated directly.

We searched carefully for the term 'providing physiological insight' (and similar) but we did not find it. The word 'insight' appears twice in the Introduction, about the literature: once that parameters are reused from previous publications without providing much insight into model behaviour, and once that sensitivity analysis provides insights into the parameter space. The third is in the 'Sepsis Population' paragraph of the Discussion, where it names how the calibration responds to incompatible targets, which is the feasibility diagnostic of Figure 10. The paper does mention that 'the calibrated populations broadly preserve the qualitative correlation structure assumed during model formulation (Table 4), which provides an internal consistency check on the generated parameter space' using the exact terminology the reviewer used.

We agree with the reviewer regarding the abstract and it now states that the parameter distributions are consistent with the theoretical haemodynamic adaptations by construction.

Lines affected: Revised manuscript, line 20 (Abstract).

Minor comments

R2-M1

> *The objective function which is minimized by gradient descent should be defined. If no gradient field is used for the optimization, the term embedded feedback controller might be more appropriate.*

Response:

We agree that the method is not a traditional gradient descent, and thank the reviewer for the suggestion. In fact, we have adopted the name the reviewer suggests, Embedded Feedback Controller (EFC), and it is no longer described as gradient descent anywhere.

Each calibrated parameter is promoted from a constant to a model state with its own error-driven evolution equation, whose sign is fixed by the qualitative correlations of Table 4 and Figure R1. The law is evaluated on the relative error alone; the sensitivity of a model output to a parameter is never formed, and the sixteen relative errors are never combined into a scalar, so there is no objective function to define. The calibrated state is the equilibrium of the coupled system, where all sixteen relative errors vanish together.

Lines affected: The rename runs through the revised manuscript at lines 12 and 32 (Abstract and keywords), 178-179, 522-552, 733, 815-816, 1043-1084 and 1359-1409, and in the captions of Figs. 5 and 6, which carry no line number. The objective function is Eq. 21, at lines 801-807.

R2-M2

> *Indicative timings should be reported for EGD for comparison to standard calibration approaches on similar models of the literature (e.g. our model provides roughly 10x speedup). Also it should be more clear how many parameters are calibrated and how many targets are imposed.*

Response:

Indicative wall-clock costs are now reported in the new Appendix subsection 'Timing and integrator comparison'. Because EFC embeds the corrections in the integration, one calibration is one simulation: calibrating one virtual subject costs 370 forward solves and 49.5 s on a single workstation CPU (Intel Core Ultra 7 155H, double precision, no GPU); the MCMC sampler costs 192 000 solves and 9077 s, and the 64-restart gradient descent 424 297 solves and 9594 s, on the same model and target set. The appendix table also gives the fixed-step RK4 and adaptive Dopri5 costs.

The parameter and target counts are now stated where the calibration is set up. The caption of Figure 6, the section 'Simulation Setup' and, for the four-method comparison, 'Convergence and method benchmarking' all give sixteen calibrated parameters against sixteen imposed physiological targets, one controller per pair.

Lines affected: Revised manuscript, lines 753-761 (Section 2.4) and 1547-1577 (Appendix A.3, with Table 8) for the timings; lines 673-686 and 703-707 (Section 2.3.2) and 795-796 (Section 2.5) for the counts. The caption of Figure 6 carries no line number.

R2-M3

> *The limitations of steady-state calibration for patient-specific clinical tracking should be mentioned, as opposed to continuous time calibration.*

Response:

On the limitations of calibrating to steady-state snapshots, the Limitations paragraph of the Discussion now states that each controller is driven by a single number per cardiac cycle, a cycle-averaged or a systolic pressure, so the calibration matches those values and cannot match the shape of the pressure or flow curve.

Lines affected: Revised manuscript, lines 1312-1317 (Discussion, *Limitations*)

R2-M4

> *The cubic correction of the controller choice should be justified against alternatives like linear, square etc.*

Response:

We thank the reviewers for pointing this out. We have rerun all calibrations with a linear version of the model and compared the results of both approaches. Table 5 reports the two laws side by side with the MCMC and gradient-descent baselines. All 1024 linear-law starts reach every target inside 1 %, at 370 forward solves per calibration, with a best error of 0.92 % and a median of 0.95 %. The cubic law costs 670 solves, returns 0.90 % at its best and 1.09 % at its median, brings 44 % of its starts inside 1 % and loses 27 of the 1024 to divergence. It also arrives later, falling below 1 % at step 512 of its 670 against step 193 of 370 under the linear law (Figure 7). The staged gain schedule each law needs is given in Appendix A.6.

Because of this we have changed the manuscript to use the linear EFC as the most efficient one, and Section 2.3.1 now says so where the text previously stated the law was purely cubic. The same section explains: the Jacobian of the pure cubic law is $3a_C\sigma_{\mathrm{rel}}^2$ and vanishes at the target, so its correction weakens as the solution is approached and the residual decays as $t^{-1/2}$, while a non-zero linear gain converges exponentially.

A square law needs no experiment to be ruled out. An even correction takes the same sign on either side of the target, so it is not monotonic in the error; the parameter would be driven through the target and keep going.

Lines affected: Revised manuscript, lines 586-596 and 601-608 (Section 2.3.1), 782-786 (Section 2.5), 820-920 (Section 3.1), 1055-1064 (Discussion), 1496-1505 (Appendix A.2) and 1672-1683 (Appendix A.6, with Table 11).

Data and code availability

R-D1

> *Have the authors made all data and (if applicable) computational code underlying the findings in their manuscript fully available?*
>
> *Reviewer #1: Yes.*
> *Reviewer #2: No: No github or code provided.*

Response:

The code is available at https://github.com/mtc8608/My-ICU-Twin, as stated in the 'Code availability' section of the submitted manuscript.
