# Supplementary material for: A physiologically constrained calibration framework for cardiovascular models applied to a paediatric synthetic sepsis population
Source: PLoS Comput Biol. 2026 Oct 7;22(10):e1014847. doi: 10.1371/journal.pcbi.1014847 (PMC13705617; doi:10.1371/journal.pcbi.1014847)
Supplement: Peer review history

# Peer review: Decision Letter 0

**DOI:** 10.1371/journal.pcbi.1014847.r001

**Authors:** Boyce Griffith, Daniele Enrico Schiavazzi

11 Jun 2026

A Physiologically Constrained Calibration Framework for Cardiovascular Models applied in Paediatric Sepsis

PLOS Computational Biology

Dear Dr. Diaz,

Thank you for submitting your manuscript to PLOS Computational Biology. After careful consideration, we feel that it has merit but does not fully meet PLOS Computational Biology's publication criteria as it currently stands. Therefore, we invite you to submit a revised version of the manuscript that addresses the points raised during the review process.

Please submit your revised manuscript by Aug 11 2026 11:59PM. If you will need more time than this to complete your revisions, please reply to this message or contact the journal office at ploscompbiol@plos.org. When you're ready to submit your revision, log on to https://www.editorialmanager.com/pcompbiol/ and select the 'Submissions Needing Revision' folder to locate your manuscript file.

Please include the following items when submitting your revised manuscript:

* A letter that responds to each point raised by the editor and reviewer(s). You should upload this letter as a separate file labeled 'Response to Reviewers'. This file does not need to include responses to formatting updates and technical items listed in the 'Journal Requirements' section below.

* A marked-up copy of your manuscript that highlights changes made to the original version. You should upload this as a separate file labeled 'Revised Manuscript with Track Changes'.

* An unmarked version of your revised paper without tracked changes. You should upload this as a separate file labeled 'Manuscript'.

We look forward to receiving your revised manuscript.

Kind regards,

Daniele Enrico Schiavazzi, Ph.D.

Academic Editor

PLOS Computational Biology

Boyce Griffith

Section Editor

PLOS Computational Biology

**Additional Editor Comments (if provided):**

**Journal Requirements:**

If the reviewer comments include a recommendation to cite specific previously published works, please review and evaluate these publications to determine whether they are relevant and should be cited. There is no requirement to cite these works unless the editor has indicated otherwise.

[Note: HTML markup is below. Please do not edit.]

**Reviewers' comments:**

Reviewer's Responses to Questions

**Comments to the Authors:**

**Please note here if the review is uploaded as an attachment.**

Reviewer #1: TITLE

A Physiologically Constrained Calibration Framework for Cardiovascular Models applied in Paediatric Sepsis

AUTHORS

Cabeleira M.T., Ray S., Ovenden N.C., Diaz-Zuccarini V.

SUMMARY

The paper introduces Embedded Gradient Descent (EGD), a calibration strategy for ODE-based lumped-parameter cardiovascular models. Rather than treating physiological parameters as fixed constants to be tuned by an external optimizer, the authors promote a selected subset of parameters to dynamical states and append to the governing ODEs a set of cubic "controller" equations that drive model outputs toward prescribed clinical targets. The sign of each controller is fixed by a qualitative compartment-level correlation matrix. The framework is demonstrated on a closed-loop nine-compartment cardiopulmonary model with 27 state variables, 29 parameters, time-varying elastance heart chambers and an event-triggered heart-rate mechanism.

The authors first show, over 100 runs with randomised initial parameters and blood-volume distributions, that calibration converges to the same target values with relative errors below 1% regardless of initialisation. They then generate three virtual pediatric cohorts (normal physiology, warm shock, and cold shock) by Latin-hypercube sampling 200 admissible target sets per cohort and calibrating one virtual subject per sample. The resulting parameter distributions are analysed for physiological consistency: total systemic resistance correlates negatively with cardiac output, compliance increases with total blood volume, and the warm/cold shock cohorts occupy resistance/compliance/elastance regimes consistent with vasodilation and vasoconstriction respectively.

The authors further argue that persistent residual errors arise only when target sets are structurally incompatible with the model (parameter saturation at bounds), and therefore serve as an interpretable diagnostic of feasibility limits rather than an optimization failure. They position EGD as a scalable foundation for virtual-population generation and future patient-specific digital-twin applications in critical care.

COMMENTS

- The validation is entirely performed using synthetic measurements. There is no comparison against real pediatric ICU recordings, nor against an independent calibration method (e.g. MCMC or multi-start gradient descent) applied to the same targets. Without such an external reference, claims of "low residual error" and "physiological consistency" are difficult to interpret as evidence of correctness. The authors should add at least one comparison against an established optimization method on an identical calibration task, and discuss the challenges they anticipate when moving to real patient data.

- The central claim that EGD "converges to a single physiological solution" needs a more careful statement. The Introduction motivates the work precisely by the fact that multiple parameter combinations reproduce the same hemodynamic targets (i.e. the presence of non-identifiable combination of parameters). EGD does not resolve this non-identifiability intrinsically; it removes it by design, through the fixed one-to-one target/parameter assignment of Fig. 6, and by leaving selected compartments uncontrolled. The uniqueness is therefore conditional on these modelling choices. The authors should make this explicit and show how sensitive the calibrated solution and the cohort-level conclusions are to alternative controller assignments.

- The model discuss by the authors is composed of resistors and capacitors, but there are no inductance elements to simulate effects due to blood inertia (typically inductors are assigned to the aortic and pulmonary arterial compartments). The authors should discuss if any changes in the proposed tuning approach would be need for RLC-based lumped parameter models.

- No formal analysis of the controller dynamics is provided. Additionally, the selected cubic law is introduced heuristically, but it is not clear why a different law would not be appropriate. Also, there is no stability or convergence argument for the sixteen coupled nonlinear controllers acting simultaneously and the statement that the scheme prevents "convergence to partial or local minima" is asserted but not demonstrated.

- The model contains no autonomic/baroreflex regulation, despite autonomic dysfunction being a defining feature of septic shock and despite the "ANS-Symp." regulatory loop being included in Fig. 1. The authors should comment on the validity of their modeling approach in light of this approximation.

- Total blood volume and its distribution across compartments are fixed to a common reference for all cohorts (Section 2.5, Limitations). The authors acknowledge this, but since inflammatory volume loss and redistribution are central to the haemodynamics of sepsis, fixing TBV partition seems to remove one of the most clinically relevant degrees of freedom. Some quantification of how strongly this assumption biases the inferred shock parameter distributions would be valuable.

- The treatment of heart rate in the calibration is unclear. Cardiac output is controlled only through stroke volume via the left-ventricular elastance E_Hl (group 4, Fig. 6), and CO = SV·HR. Table 2, however, prescribes HR target ranges, and Fig. 6 lists no HR controller. Is HR a fixed input drawn from the target ranges, or is it calibrated? Please clarify where the HR target enters the procedure and how CO targets are met when HR is itself constrained. CHECK!!!!

- The reported per-cohort simulation protocol (2200 s of simulated time, 200 s transient, Δt = 0.0005 s) implies a substantial computational cost per virtual subject. Since the stated long-term goal is patient-specific digital twins in acute care, the authors should report wall-clock cost per calibration and discuss whether the approach is fast enough to be clinically useful, and how it scales relative to the optimization methods it is meant to replace.

- Figure 1 mixes mechanisms that are in the model (resistance, compliance, cardiac output, stroke volume) with mechanisms that are not (autonomic vasoconstriction/vasodilation). I would revise this schematic so it distinguishes clearly between physiological background and the components actually implemented, and add explicit references to the corresponding Sections/Equations.

- The leaving-uncontrolled of the thoracic veins (Pvt) and pulmonary veins (Pvp) is justified only qualitatively ("negligible pressure drops"). Given that nine compartments but only sixteen of the available parameters are controlled, a brief sensitivity check on whether this choice affects the calibrated venous/capillary distribution would help substantiate the claim.

- Section 2.4. Use of the explicit Euler scheme is quite unusual for lumped parameter models which may exhibit stiffness due to perfect unidirectional valves and due to changes in overall RC constants for compartments, following opening and closing of valves. The authors should report a comparison with, e.g., higher order numerical integrators with adaptive time stepping (e.g. RK4-5).

- Fig. 8: please state explicitly what the colors correspond to.

SYNTAX

- Introduction: please review the sentence "Some notable examples in literature that do provide solutions to the parametrization of ODE models usually also very much simplified [22, 23]".

- Introduction: "for the the model".

- Section 2.3.1: "replaced with any another monotonic".

- Page 21: Appendix A.2: "The systemic arterial pulse pressure variable appear", the sentence appears to be incomplete.

- Page 1 (Introduction): the sentence "In Djoumessi et al. The addition of autonomic control to these models allows the study of the response to hypercapnia [14, 15, 16]..." is a broken fragment, and the citation does not appear to match the named author.

- Reference [40] (Khonsary, Guyton and Hall): the DOI is rendered with unescaped LaTeX, "10.4103/sni.sni{\_}327{\_}17" — should be "10.4103/sni.sni_327_17".

- Reference [30] (Shankar-Hari et al.): "Developing a newdefinition and assessing newclinical criteria for Septic shock" — missing spaces: "new definition" and "new clinical".

- Reference [29]: author names are run together — "Christopherwarren Seymour" and "Tomvan Der Poll" should be "Christopher Warren Seymour" and "Tom van der Poll".

TITLE, ABSTRACT, REFERENCES

- TITLE: maybe use "applied to Pediatric Sepsis" instead of "applied in Pediatric Sepsis"? just a suggestion.

- ABSTRACT: appropriate.

- REFERENCES: Appropriate in scope and coverage.

RECOMMENDATION

The paper is interesting and well written. However, it contains substantial gaps that should be addressed before it can be accepted. The most important are: (1) the validation is entirely synthetic and self-referential, with no real-data confirmation and no comparison against an independent calibration method; (2) The method appears to be "ad hoc" for a specific model formulation and optimization process. Identifiability and convergence are not handled directly, e.g., by characterizing a multiplicity of physiological parameter combinations leading to the same observations. The formulation of the model plus the additional controller equations act as a regularizer to promote only a subset of such combinations. Thus, the use of synthetic measurements generated by the same model formulation does not seem appropriate to validate the approach. I therefore recommend major revision, and encourage the authors to address the comments and questions above.

Reviewer #2: The paper presents an EGD approach for calibrating patient parameters of a lumped cardiovascular model. The model is applied on a virtual pediatric sepsis population, generated using parameter ranges of the literature. The direction of the work is interesting for tuning patient-specific solutions and clinical digital-twin applications. However, there are currently several claims which would need stronger validation or softening in order to refine the work. My main recommendations are the following:

1. The work is based on a numerical model using a randomly sampled virtual population as an internal validation but is not validated on an external sepsis real dataset. Many sampled internal variables are unobserved, which may result in numerous non-physiological combinations and missed joint dependencies. Therefore this is not a pediatric sepsis application in the clinical sense, so I would suggest the authors to provide a disclaimer about this in the text, or provide an external clinical validation to justify the virtual cohort sampling. I believe that the title is also a bit misleading, as the reader expects to see some clinical results on pediatric sepsis. I would recommend the "application" part to not be in the title, or to be explicit about its in-silico nature.

2. The authors argue that EGD converges to a unique steady solution, claiming that for any admissible target set, there is a unique physiological solution. This claim relies on qualitative monotonicity of parameter-variable relationship and on the fact that multiple controllers impose simultaneous constraints. However, this does not prove global uniqueness in a coupled nonlinear ODE system. The authors report an empirical convergence experiment with 100 randomized initializations for a single normal target set, but this is not enough to guarantee global behavior. I recommend the general uniqueness claim to be weakened to "empirical testing cases results" and some additional tests to be included regarding broader target ranges, controller gains and target sets.

3. The authors say that the parameter bounds are introduced "solely as numerical safeguards" and are chosen wide enough to let the solution space unconstrained. But later on, saturation is used precisely as the condition for infeasibility. Moreover, the authors say that all incompatible target combinations are associated with persistent residuals. Such a diagnostic could be valuable but the threshold that distinguishes infeasibility from slow convergence, poor gain selection, or numerical instabilities should be justified. Adding an experiment of custom cases verifying that feasible targets always have low residuals while infeasible target have high residuals (for fixed model settings) would strengthen the claim.

4. The authors should be more specific on which parameter patterns emerge from the coupled model and which are just expected algebraic results from the prescribed target ranges. Phrases like "providing physiological insight" should not apply to internal consistency and numerical sanity checks. In the Abstract the authors state that the resulting parameter distributions are consistent with known pediatric sepsis adaptations, including vascular resistance, compliance etc, but this is to be expected by construction of the parameter ranges and acts more as a cyclic confirmation, not a clinical insight.

Minor comments:

- The objective function which is minimized by gradient descent should be defined. If no gradient field is used for the optimization, the term embedded feedback controller might be more appropriate.

- Indicative timings should be reported for EGD for comparison to standard calibration approaches on similar models of the literature (e.g. our model provides roughly 10x speedup). Also it should be more clear how many parameters are calibrated and how many targets are imposed.

- The limitations of steady-state calibration for patient-specific clinical tracking should be mentioned, as opposed to continuous time calibration.

- The cubic correction of the controller choice should be justified against alternatives like linear, square etc.

In general this is an ambitious methodology which would benefit from simplifying/focusing the objectives on the modeling aspect, and not overclaiming direct clinical application. I believe that the main weaknesses are certain core claims which appear strong but unjustified, like the uniqueness, infeasibility criteria and the physiological grounding of the virtual cohort. The paper should make a more careful distinction between successful calibration of internal variables, physiological plausibility and clinical applicability. Thus, I recommend this paper to be published on the PLOS Computational Biology journal after the above comments have been addressed.

**********

**Have the authors made all data and (if applicable) computational code underlying the findings in their manuscript fully available?**

The PLOS Data policy requires authors to make all data and code underlying the findings described in their manuscript fully available without restriction, with rare exception (please refer to the Data Availability Statement in the manuscript PDF file). The data and code should be provided as part of the manuscript or its supporting information, or deposited to a public repository. For example, in addition to summary statistics, the data points behind means, medians and variance measures should be available. If there are restrictions on publicly sharing data or code —e.g. participant privacy or use of data from a third party—those must be specified.

Reviewer #1: Yes

Reviewer #2: **No:**  No github or code provided.

**********

PLOS authors have the option to publish the peer review history of their article (what does this mean?). If published, this will include your full peer review and any attached files.

If you choose “no”, your identity will remain anonymous but your review may still be made public.

**Do you want your identity to be public for this peer review?**  For information about this choice, including consent withdrawal, please see our Privacy Policy.

Reviewer #1: No

Reviewer #2: No

[NOTE: If reviewer comments were submitted as an attachment file, they will be attached to this email and accessible via the submission site. Please log into your account, locate the manuscript record, and check for the action link "View Attachments". If this link does not appear, there are no attachment files.]

**Figure resubmission:**

**Reproducibility:**

?>

# Author comment: Author response to Decision Letter 1

**DOI:** 10.1371/journal.pcbi.1014847.r002

1 Sep 2026

Attachment Submitted filename: responseLetter.pdf

# Peer review: Decision Letter 1

**DOI:** 10.1371/journal.pcbi.1014847.r003

**Authors:** Boyce Griffith, Daniele Enrico Schiavazzi

23 Sep 2026

Dear Dr Diaz,

We are pleased to inform you that your manuscript 'A Physiologically Constrained Calibration Framework for Cardiovascular Models Applied to a Paediatric Synthetic Sepsis population' has been provisionally accepted for publication in PLOS Computational Biology.

Before your manuscript can be formally accepted you will need to complete some formatting changes, which you will receive in a follow up email. A member of our team will be in touch with a set of requests.

Please note that your manuscript will not be scheduled for publication until you have made the required changes, so a swift response is appreciated.

IMPORTANT: The editorial review process is now complete. PLOS will only permit corrections to spelling, formatting or significant scientific errors from this point onwards. Requests for major changes, or any which affect the scientific understanding of your work, will cause delays to the publication date of your manuscript.

Should you, your institution's press office or the journal office choose to press release your paper, you will automatically be opted out of early publication. We ask that you notify us now if you or your institution is planning to press release the article. All press must be co-ordinated with PLOS.

Thank you again for supporting Open Access publishing; we are looking forward to publishing your work in PLOS Computational Biology.

Best regards,

Daniele Enrico Schiavazzi, Ph.D.

Academic Editor

PLOS Computational Biology

Boyce Griffith

Section Editor

PLOS Computational Biology

***********************************************************

Reviewer's Responses to Questions

**Comments to the Authors:**

**Please note here if the review is uploaded as an attachment.**

Reviewer #1: The authors have address all my questions and comments. The paper can be published in its revised form.

Reviewer #2: The authors have addressed the necessary comments.

**********

**Have the authors made all data and (if applicable) computational code underlying the findings in their manuscript fully available?**

The PLOS Data policy requires authors to make all data and code underlying the findings described in their manuscript fully available without restriction, with rare exception (please refer to the Data Availability Statement in the manuscript PDF file). The data and code should be provided as part of the manuscript or its supporting information, or deposited to a public repository. For example, in addition to summary statistics, the data points behind means, medians and variance measures should be available. If there are restrictions on publicly sharing data or code —e.g. participant privacy or use of data from a third party—those must be specified.

Reviewer #1: None

Reviewer #2: None

**********

PLOS authors have the option to publish the peer review history of their article (what does this mean?). If published, this will include your full peer review and any attached files.

If you choose “no”, your identity will remain anonymous but your review may still be made public.

**Do you want your identity to be public for this peer review?**  For information about this choice, including consent withdrawal, please see our Privacy Policy.

Reviewer #1: No

Reviewer #2: No

# Editor report: Acceptance letter

**DOI:** 10.1371/journal.pcbi.1014847.r004

**Authors:** Boyce Griffith, Daniele Enrico Schiavazzi

PCOMPBIOL-D-26-00500R1

A Physiologically Constrained Calibration Framework for Cardiovascular Models Applied to a Paediatric Synthetic Sepsis population

Dear Dr Diaz,

I am pleased to inform you that your manuscript has been formally accepted for publication in PLOS Computational Biology. Your manuscript is now with our production department and you will be notified of the publication date in due course.

The corresponding author will soon be receiving a typeset proof for review, to ensure errors have not been introduced during production. Please review the PDF proof of your manuscript carefully, as this is the last chance to correct any errors. Please note that major changes, or those which affect the scientific understanding of the work, will likely cause delays to the publication date of your manuscript.

Soon after your final files are uploaded, unless you have opted out, the early version of your manuscript will be published online. The date of the early version will be your article's publication date. The final article will be published to the same URL, and all versions of the paper will be accessible to readers.

For Research, Software, and Methods articles, you will receive an invoice from PLOS for your publication fee after your manuscript has reached the completed accept phase. If you receive an email requesting payment before acceptance or for any other service, this may be a phishing scheme. Learn how to identify phishing emails and protect your accounts at https://explore.plos.org/phishing.

Thank you again for supporting PLOS Computational Biology and open-access publishing. We are looking forward to publishing your work!

With kind regards,

Sharmila Kamatchi

PLOS Computational Biology | Carlyle House, Carlyle Road, Cambridge CB4 3DN | United Kingdom ploscompbiol@plos.org | Phone +44 (0) 1223-442824 | ploscompbiol.org | @PLOSCompBiol
